# SLC44A2 regulates vascular smooth muscle cell phenotypic switching and aortic aneurysm

**DOI:** 10.1172/JCI173690

**Published:** 2024-06-25

**Authors:** Tianyu Song, Shuang Zhao, Shanshan Luo, Chuansheng Chen, Xingeng Liu, Xiaoqi Wu, Zhongxu Sun, Jiawei Cao, Ziyu Wang, Yineng Wang, Bo Yu, Zhiren Zhang, Xiaolong Du, Xiaoqiang Li, Zhijian Han, Hongshan Chen, Feng Chen, Liansheng Wang, Hong Wang, Kangyun Sun, Yi Han, Liping Xie, Yong Ji

**Affiliations:** 1 Gusu School, Nanjing Medical University, Suzhou, China.; 2 Key Laboratory of Cardiovascular and Cerebrovascular Medicine, Key Laboratory of Targeted Intervention of Cardiovascular Disease, Collaborative Innovation Center for Cardiovascular Disease Translational Medicine, Nanjing Medical University, Nanjing, Jiangsu, China.; 3 State Key Laboratory of Frigid Zone Cardiovascular Diseases (SKLFZCD), and; 4 Department of Cardiology, Central Laboratory, The First Affiliated Hospital of Harbin Medical University, NHC Key Laboratory of Cell Transplantation, Harbin Medical University, China.; 5 Department of Vascular Surgery, The Affiliated Nanjing Drum Tower Hospital, Nanjing University Medical School, Nanjing, China.; 6 Department of Urology, First Affiliated Hospital of Nanjing Medical University, Nanjing, China.; 7 Department of Forensic Medicine, and; 8 Department of Cardiology, the First Affiliated Hospital of Nanjing Medical University, Nanjing Medical University, Nanjing, China.; 9 Center for Metabolic Disease Research, Department of Microbiology and Immunology, Temple University Lewis Katz School of Medicine, Philadelphia, Pennsylvania, USA.; 10 Department of Cardiology, The Affiliated Suzhou Hospital of Nanjing Medical University, Suzhou Municipal Hospital, Gusu School, Nanjing Medical University, Suzhou, China.; 11 Critical Care Department, The Second Affiliated Hospital of Harbin Medical University, Harbin, Heilongjiang, China.

**Keywords:** Vascular biology, Cardiovascular disease, Signal transduction

## Abstract

Aortic aneurysm is a life-threatening disease with limited interventions that is closely related to vascular smooth muscle cell (VSMC) phenotypic switching. SLC44A2, a member of the solute carrier series 44 (SLC44) family, remains undercharacterized in the context of cardiovascular diseases. Venn diagram analysis based on microarray and single-cell RNA sequencing identified SLC44A2 as a major regulator of VSMC phenotypic switching in aortic aneurysm. Screening for *Slc44a2* among aortic cell lineages demonstrated its predominant location in VSMCs. Elevated levels of SLC44A2 were evident in the aorta of both patients with abdominal aortic aneurysm and angiotensin II–infused (Ang II–infused) *Apoe^–/–^* mice. In vitro, SLC44A2 silencing promoted VSMCs toward a synthetic phenotype, while SLC44A2 overexpression attenuated VSMC phenotypic switching. VSMC-specific SLC44A2-knockout mice were more susceptible to aortic aneurysm under Ang II infusion, while SLC44A2 overexpression showed protective effects. Mechanistically, SLC44A2’s interaction with NRP1 and ITGB3 activates TGF-β/SMAD signaling, thereby promoting contractile gene expression. Elevated SLC44A2 in aortic aneurysm is associated with upregulated runt-related transcription factor 1 (RUNX1). Furthermore, low-dose lenalidomide (LEN; 20 mg/kg/day) suppressed aortic aneurysm progression by enhancing SLC44A2 expression. These findings reveal that the SLC44A2-NRP1-ITGB3 complex is a major regulator of VSMC phenotypic switching and provide a potential therapeutic approach (LEN) for aortic aneurysm treatment.

## Abstract 2

The SLC44A2/NRP1/ITGB3 complex is a major regulator of vascular smooth muscle cell phenotypic switching and a target of lenalidomide in aortic aneurysm treatment.

## Graphical abstract

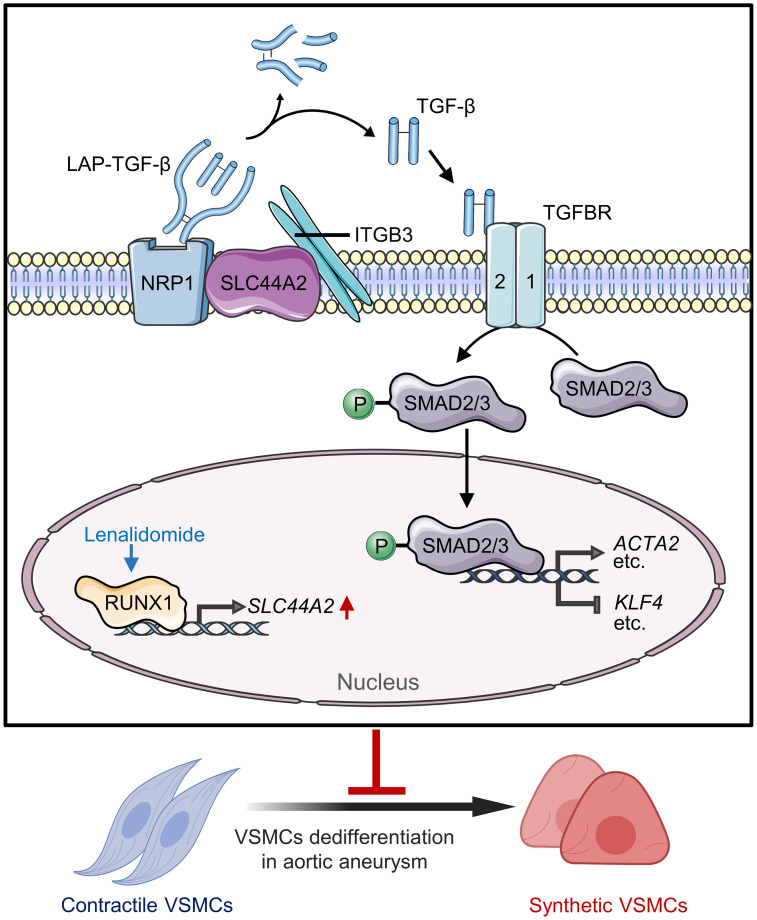

## Introduction

Aortic aneurysm is defined as a localized enlargement of the aorta by at least 50% compared with the expected diameter in age-matched and sex-matched healthy individuals (1). Patients with aortic aneurysm typically remain asymptomatic until catastrophic complications like aortic rupture or dissection occur (2). Currently, therapeutic options for aortic aneurysm are limited to surgical interventions, with a lack of established pharmacological treatments to inhibit the aneurysm’s progressive growth or prevent rupture (3). Thus, elucidating the regulatory mechanisms driving aortic aneurysm formation is essential for developing novel medical therapies.

Vascular smooth muscle cells (VSMCs) are critical to maintaining the vasoconstriction and vasodilatation of healthy vessels, exhibiting remarkable plasticity in response to environmental cues (4). Under pathological conditions, contractile VSMCs can dedifferentiate into a synthetic phenotype to produce elastolytic and proinflammatory factors, a process termed phenotypic switching (5). This switching contributes to extracellular matrix degradation, vascular inflammation, cell migration, and apoptosis, which are responsible for the occurrence and aggravation of aortic aneurysm (6). Therefore, seeking the key nodes in VSMC phenotypic switching may provide potential targets for aortic aneurysm management.

SLC44A2 belongs to the solute carrier series 44 (SLC44) family and was discovered as a supporting-cell antigen in the inner ear (7). *Slc44a2^–/–^* mice show hair cell death and hearing loss (8). Recent studies have linked *SLC44A2* single-nucleotide polymorphism (SNP) loci with venous thrombosis and Ménière disease (9, 10). However, the role of SLC44A2 in the cardiovascular system remains undefined. Here, we report SLC44A2 as a key regulator of VSMC phenotypic switching, participating in the progression of aortic aneurysm. SLC44A2 interacts with neuropilin-1 (NRP1) to activate transforming growth factor β (TGF-β) signaling in an integrin β3–dependent (ITGB3-dependent) manner, which maintains VSMCs’ contractile phenotype and alleviates aortic aneurysm. Additionally, lenalidomide (LEN) administration enhances SLC44A2 expression primarily through runt-related transcription factor 1 (RUNX1), leading to improvements in aortic aneurysm conditions, which provides a potential new therapeutic strategy for aortic aneurysm.

## Results

### Increased SLC44A2 in VSMCs is associated with aortic aneurysm.

**Figure 1:**
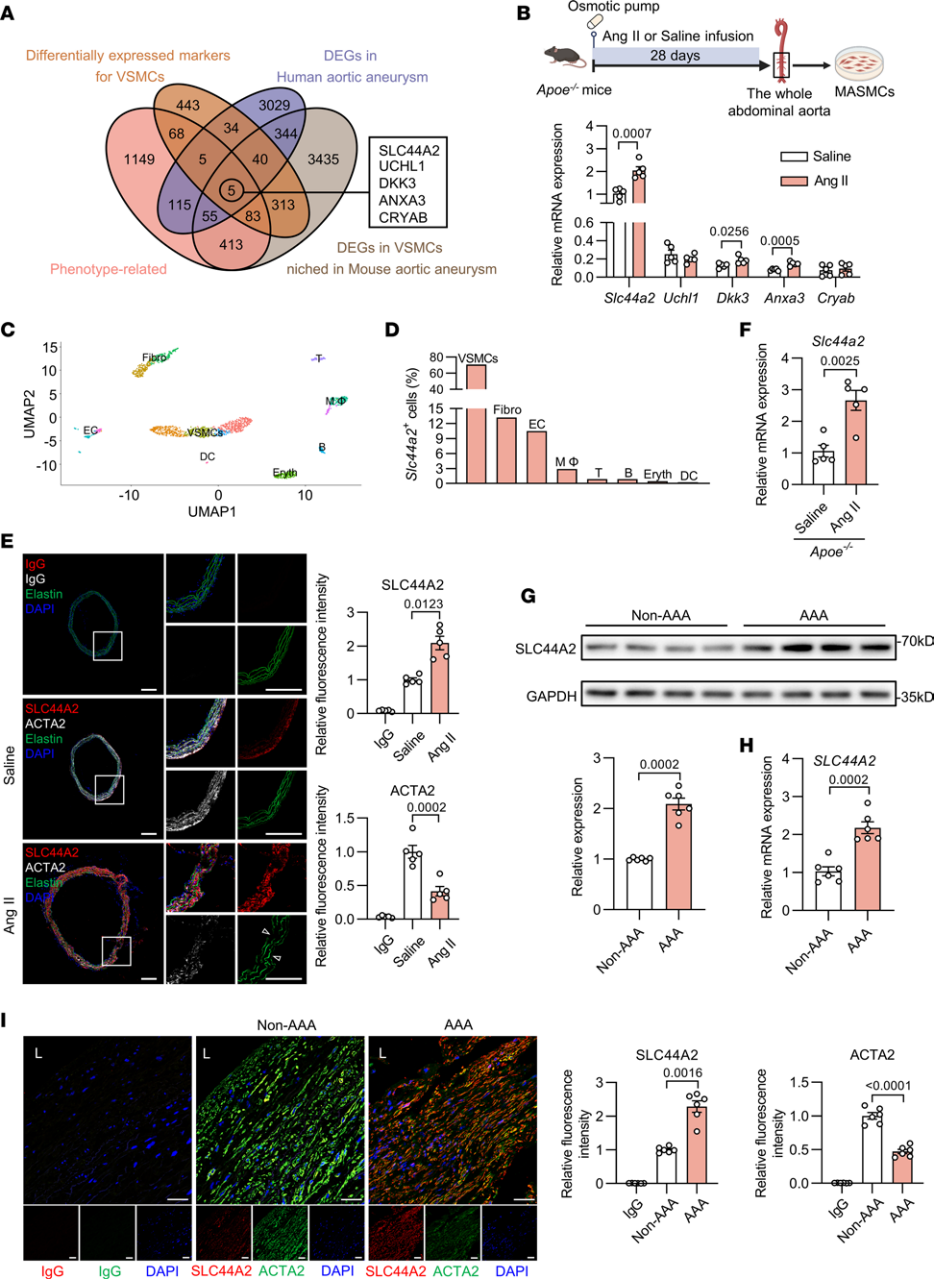
Aortic SLC44A2 expression is elevated in aortic aneurysm. (**A**) Venn diagram showing the overlap between VSMC phenotype–related genes, differentially expressed markers for VSMCs, DEGs in human aortic aneurysm (GSE47472), and DEGs in VSMCs niched in mouse aortic aneurysm (GSE186865). (**B**) MASMCs were isolated from the whole abdominal aortas of saline- or Ang II–infused mice. The mRNA levels of *Slc44a2*, *Uchl1*, *Dkk3*, *Anxa3*, and *Cryab* were detected by qRT-PCR. *n* = 5. (**C**) Uniform manifold approximation and projection (UMAP) visualization of single cells from abdominal aortic tissue of mice (GSE152583). Cells were partitioned into 8 major lineages: vascular smooth muscle cells (VSMCs), fibroblasts (Fibro), endothelial cells (EC), macrophages (MΦ), T cells (T), B cells (B), erythrocytes (Eryth), and dendritic cells (DC). (**D**) *Slc44a2* expression among distinct cellular populations. (**E** and **F**) *Apoe^–/–^* mice were infused with saline or Ang II for 28 days. (**E**) Immunofluorescent staining for SLC44A2 (red), ACTA2 (white), and staining with DAPI (blue) in the suprarenal abdominal aorta. Elastic fibers are green (autofluorescence). Arrowheads indicate elastin breaks. IgG was used as the isotype control. Scale bars: 200 μm. *n* = 5. (**F**) *Slc44a2* mRNA level in aorta. *n* = 5. (**G**) Western blot analysis of SLC44A2 expression in the aorta from non-AAA groups and AAA patients. *n* = 6. (**H**) *SLC44A2* mRNA level in the aorta from non-AAA groups and AAA patients. *n* = 6. (**I**) Immunofluorescent staining for SLC44A2 (red), ACTA2 (green), and staining with DAPI (blue) in the aortic media of non-AAA groups and AAA patients. IgG was used as the isotype control. L, lumen. Scale bars: 40 μm. *n* = 6. Differences were analyzed by unpaired, 2-tailed Student’s *t* test (**B**, **F**, and **H**), Welch’s ANOVA followed by Tamhane’s T2 multiple-comparison test or 1-way ANOVA followed by Tukey’s multiple-comparison test (**E**), Welch’s *t* test (**G**), or Welch’s ANOVA followed by Tamhane’s T2 multiple-comparison test (**I**).

To identify the candidate genes potentially related to VSMC phenotypic switching in aortic aneurysm, we initially analyzed 2 data sets, VSMC phenotype–related genes (11) and differentially expressed markers for VSMCs (12), to screen for critical genes that are both involved in VSMC phenotypic switching and highly expressed in VSMCs. Next, using the NCBI Gene Expression Omnibus (GEO) database, we gathered differentially expressed genes (DEGs) in human aortic aneurysm (GSE47472) and DEGs in VSMCs niched in mouse aortic aneurysm (GSE186865) to uncover aortic aneurysm–relevant transcriptional signatures. As shown by Venn diagram, *SLC44A2*, *UCHL1*, *DKK3*, *ANXA3*, and *CRYAB* were overlapped from the aforementioned 4 data sets and emerged as main candidate genes linking VSMC phenotypic switching to aortic aneurysm (Figure 1A). We then detected these 5 genes in primary mouse aortic smooth muscle cells (MASMCs) from the whole aortas of saline-infused mice or angiotensin II–infused (Ang II–infused) aortic aneurysm mouse models via quantitative real-time polymerase chain reaction (qRT-PCR), and found that SLC44A2 was the most abundant and increased with the highest fold change in MASMCs from Ang II–infused apolipoprotein E–knockout (*Apoe^–/–^*) mice (Supplemental Figure 1A; supplemental material available online with this article; https://doi.org/10.1172/JCI173690DS1). Consistent results were also observed in MASMCs isolated from the whole abdominal aortas (Figure 1B and Supplemental Figure 1B). Taken together, these results directed our focus toward the functional role of SLC44A2 in aortic aneurysm.

To generate a vista of SLC44A2 abundance among cell lineages niched within the vascular wall, we reanalyzed a single-cell RNA sequencing (scRNA-seq) data set of murine abdominal aortas (12). After applying integrative cell clustering analysis, results were visualized as a uniform manifold approximation and projection (UMAP) plot (Figure 1C). Screening across 8 major lineages demonstrated predominant SLC44A2 accumulation in VSMCs (Figure 1D). Meanwhile, immunostaining of suprarenal abdominal aortas showed an elevation of SLC44A2 expression in Ang II–infused mice, which coincided with reduced ACTA2 expression, an indicator of medial degeneration (Figure 1E). The SLC44A2 mRNA level was also elevated in aortas from Ang II–infused mice (Figure 1F). Importantly, we further verified that SLC44A2 protein and mRNA levels were significantly increased in patients with abdominal aortic aneurysm (AAA) (Figure 1, G and H), with immunostaining indicating higher SLC44A2 levels in the media layer of human AAA aortas compared with controls (Figure 1I). In vitro, SLC44A2 was increased after Ang II treatment in human aortic smooth muscle cells (HASMCs) at 6 hours and reached a peak at 24 hours (Supplemental Figure 1C). These results suggest that upregulation of SLC44A2 in VSMCs may be involved in the progression of aortic aneurysm.

### SLC44A2 modulates VSMC phenotypic switching.

**Figure 2:**
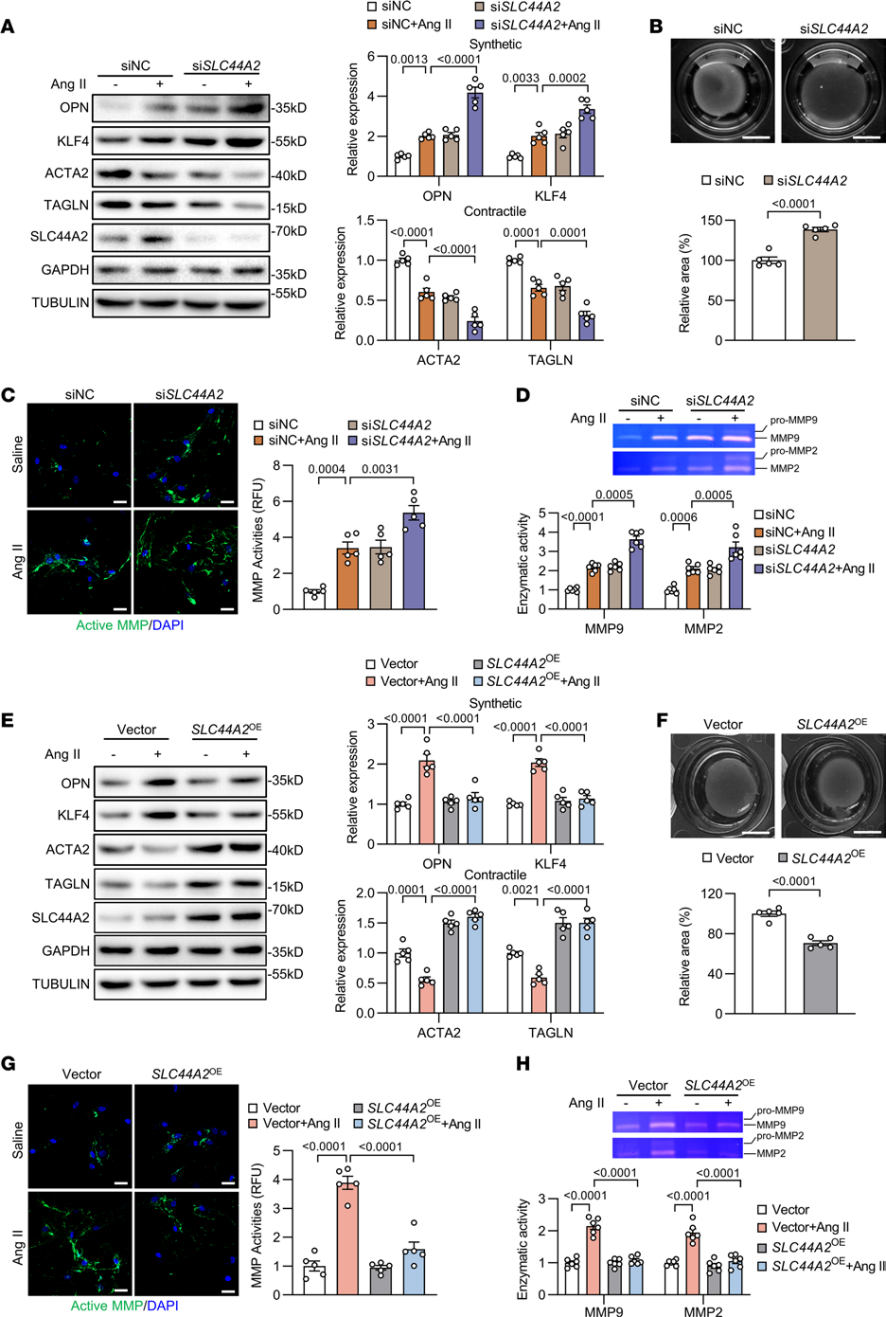
SLC44A2 maintains the contractile phenotype of VSMCs. (**A**–**D**) HASMCs were transfected with siRNA against SLC44A2 (si*SLC44A2*) or negative control (siNC), and then treated with Ang II (1 μM, 24 hours). (**A**) The synthetic and contractile markers were detected by Western blotting. *n* = 5. (**B**) Contraction of HASMCs grown in collagen discs was assessed and quantified by gel area. Scale bars: 5 mm. *n* = 5. (**C**) Immunofluorescence images of in situ zymography (DQ gelatin) in HASMCs. MMP activity was quantified by immunofluorescence intensity. RFU, relative fluorescence units. Scale bars: 40 μm. *n* = 5. (**D**) The activity of MMP2 and MMP9 in culture medium was measured by gel zymography. *n* = 6. (**E**–**H**) HASMCs were infected with lentivirus containing empty vector or SLC44A2-encoding plasmids to overexpress SLC44A2 (*SLC44A2*^OE^), and then treated with Ang II (1 μM, 24 hours). (**E**) The synthetic and contractile markers were detected by Western blotting. *n* = 5. (**F**) Contraction of HASMCs grown in collagen discs was assessed and quantified by gel area. Scale bars: 5 mm. *n* = 5. (**G**) Immunofluorescence images of in situ zymography (DQ gelatin) in HASMCs. MMP activity was quantified by immunofluorescence intensity. Scale bars: 40 μm. *n* = 5. (**H**) The activity of MMP2 and MMP9 in culture medium was measured by gel zymography. *n* = 6. Differences were analyzed by 1-way ANOVA followed by Tukey’s multiple-comparison test (**A**, **C**, **E**, **G**, and **H**), unpaired, 2-tailed Student’s *t* test (**B** and **F**), or Welch’s ANOVA followed by Tamhane’s T2 multiple-comparison test or 1-way ANOVA followed by Tukey’s multiple-comparison test (**D**).

VSMCs demonstrate marked phenotypic modulation, ranging from a contractile state in quiescent mature arteries to a proliferative and synthetic state in aortic aneurysm. To investigate the role of elevated SLC44A2 in VSMC phenotypic switching, we first tried to assess VSMC phenotype by SLC44A2 knockdown. However, SLC44A2 silencing led to a further increase in VSMC synthetic markers (OPN and KLF4) induced by Ang II and repressed contractile markers (ACTA2 and TAGLN) (Figure 2A). Cell-embedded collagen gel contraction assay showed that SLC44A2 knockdown reduced HASMC contractility (Figure 2B). Ang II–induced matrix metalloproteinase (MMP) activation was further enhanced by SLC44A2 knockdown, as shown by in situ zymography (Figure 2C) and gel zymography (Figure 2D). Conversely, SLC44A2 overexpression could ameliorate Ang II–induced VSMC phenotypic switching (Figure 2E). HASMCs with SLC44A2 overexpression exhibited higher contractile capacity (Figure 2F) and a reduction in MMP activities (Figure 2, G and H). These findings suggest that increased SLC44A2, as a compensatory mechanism, during aortic aneurysm progression may play a protective role in maintaining the contractile phenotype of VSMCs.

To further elucidate the role of SLC44A2 in VSMC phenotypic switching, we reintroduced SLC44A2 in SLC44A2-knockdown HASMCs. The result showed that SLC44A2 reexpression reversed the increase in synthetic markers and decreased contractile markers induced by SLC44A2 knockdown (Supplemental Figure 2A). Meanwhile, SLC44A2 overexpression mitigated the aggravated MMP activity caused by SLC44A2 knockdown (Supplemental Figure 2B). These results suggested that overexpression of SLC44A2 can counterbalance the effects of its knockdown, thereby rescuing VSMCs from phenotypic switching. Next, we isolated primary MASMCs from WT and *Slc44a2*^KO^ mice to evaluate the effect of SLC44A2 on phenotypic switching in mouse-derived cells. Compared with WT MASMCs, *Slc44a2*^KO^ mouse–derived MASMCs exhibited higher synthetic marker and lower contractile marker expression upon Ang II stimulation, which was reversed by SLC44A2 overexpression (Supplemental Figure 2C). Additionally, SLC44A2 overexpression in WT MASMCs could alleviate Ang II–induced VSMC phenotypic switching (Supplemental Figure 2D). These results demonstrate the vital function of SLC44A2 in preserving the contractile phenotype of VSMCs.

### VSMC-specific SLC44A2 overexpression ameliorates Ang II–induced aortic aneurysm.

**Figure 3:**
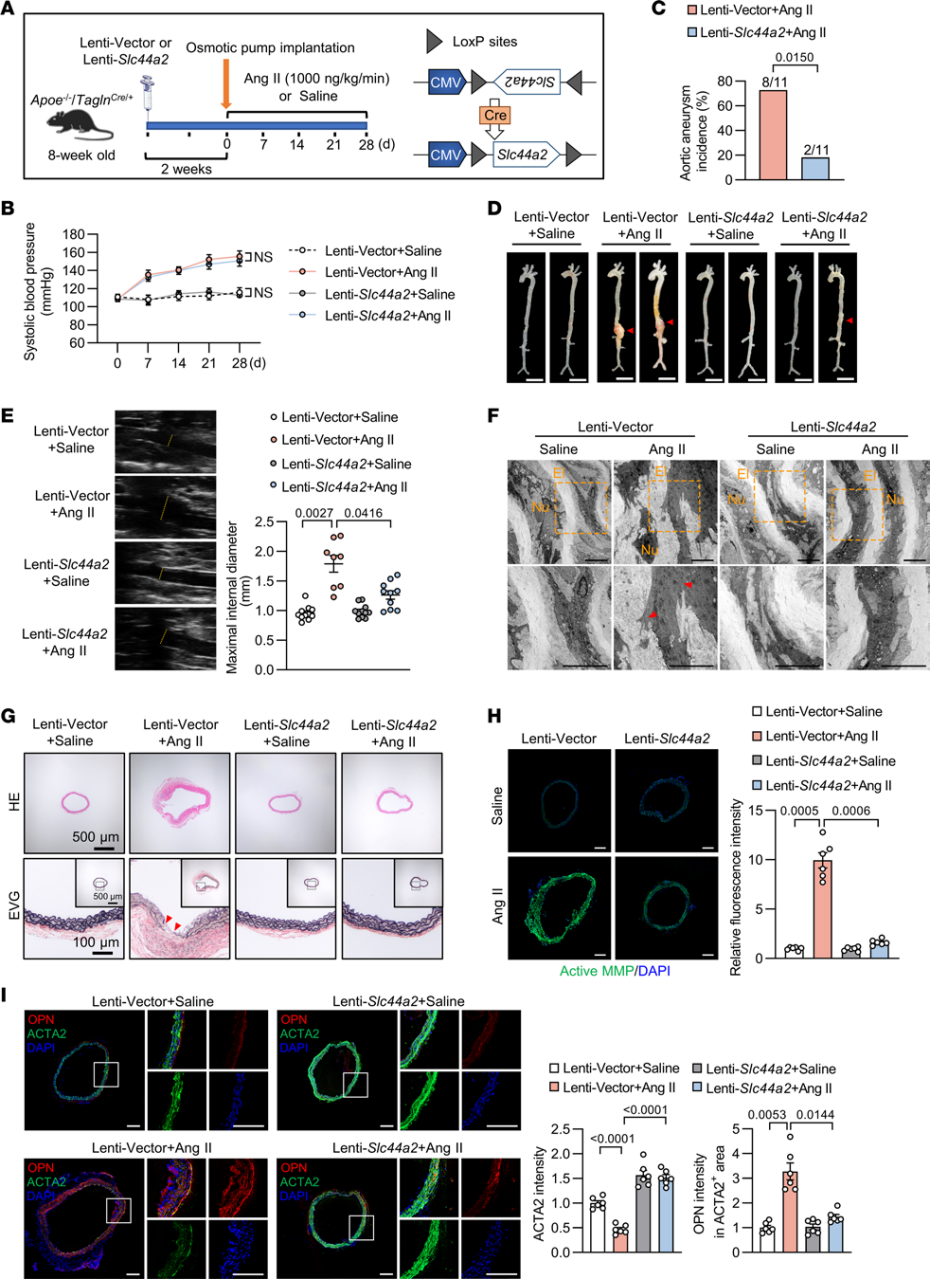
VSMC overexpression of SLC44A2 moderates aortic aneurysm in Ang II–infused *Apoe^–/–^* mice. (**A**) Eight-week-old male *Apoe^–/–^* *Tagln^Cre/+^* mice were intravenously injected with lentivirus containing control vector or the reverse *Slc44a2* sequence with 2 *loxP* sites. After 2 weeks, osmotic pumps were implanted subcutaneously to infuse saline or Ang II (1,000 ng/kg/min) for 28 days. (**B**) The systolic blood pressure at 0, 7, 14, 21, and 28 days after osmotic pump implantation. *n* = 8–11. NS, no significance. (**C**) The incidence of aortic aneurysm in Ang II–infused mice. *n* = 11. (**D**) Representative morphology of aortas from saline- or Ang II–infused mice. Scale bars: 5 mm. *n* = 11. (**E**) Ultrasound images and inner diameter quantification of the suprarenal abdominal aorta. *n* = 8–11. (**F**) Electron microscopic images of the suprarenal abdominal aorta. Red arrowheads indicate elastin breaks. El, elastin; Nu, nucleus. Scale bars: 5 μm. *n* = 3. (**G**) Hematoxylin and eosin (H&E) and elastic Verhoeff–Van Gieson (EVG) staining of the suprarenal abdominal aorta. Red arrowheads indicate elastin breaks. *n* = 6. (**H**) Immunofluorescence images of in situ zymography (DQ gelatin, green) in the suprarenal abdominal aorta. Scale bars: 200 μm. *n* = 6. (**I**) Immunofluorescent staining for OPN (red), ACTA2 (green), and staining with DAPI (blue) in the suprarenal abdominal aorta. Scale bars: 200 μm. *n* = 6. Differences were analyzed by 2-way ANOVA with mixed effects followed by Tukey’s multiple-comparison test (**B**), Fisher’s exact test (**C**), Welch’s ANOVA followed by Tamhane’s T2 multiple-comparison test (**E** and **H**), or 1-way ANOVA followed by Tukey’s multiple-comparison test or Welch’s ANOVA followed by Tamhane’s T2 multiple-comparison test (**I**).

To delineate the function of SLC44A2 in VSMCs during aortic aneurysm, *Apoe^–/–^* *Tagln^Cre/+^* mice were intravenously injected with lentivirus carrying empty vector (Lenti-Vector) or SLC44A2 overexpression plasmid (Lenti-*Slc44a2*) containing 2 *loxP* sites that can be recognized by Cre recombinase in VSMCs, facilitating SLC44A2 transcription (Figure 3A). SLC44A2 overexpression in the tunica media was verified by immunostaining (Supplemental Figure 3A). Body weight remained consistent across all groups (Supplemental Figure 3B), and blood pressure increased similarly upon Ang II infusion in both Lenti-Vector and Lenti-*Slc44a2* mice (Figure 3B). Strikingly, SLC44A2 overexpression in VSMCs blunted aortic aneurysm incidence induced by Ang II (Figure 3C). In the presence of Ang II, mice developed aortic dilations and aneurysms, which was mitigated in Lenti-*Slc44a2* mice (Figure 3D). The aortic rupture rate in Lenti-Vector mice was 27.27%, while it was 9.09% in Lenti-*Slc44a2* mice after Ang II infusion (Supplemental Figure 3C). To quantify the dilation of aortic aneurysm, we performed ultrasound imaging every 14 days to follow the changes in aortic diameter. Compared with the Lenti-Vector group, SLC44A2 overexpression inhibited aortic enlargement at 14 days and 28 days after infusion of Ang II (Supplemental Figure 3D and Figure 3E). Elastin damage is preferentially associated with progressive aortic dilatation. Notably, electron microscopic analysis revealed that the severe disruption of elastin fibers typically induced by Ang II was substantially mitigated in the aortas of Lenti-*Slc44a2* mice (Figure 3F). Hematoxylin and eosin (H&E) staining indicated alleviated aortic dilatation in Lenti-*Slc44a2* mice in response to Ang II. Concomitantly, elastic Verhoeff–Van Gieson (EVG) staining of aortic sections from Lenti-*Slc44a2* mice showed reduced media degeneration (Figure 3G). An association of increased MMP activity with aortic aneurysm is well documented, where MMP promotes matrix degradation and impairs the integrity of the arterial wall. MMP activities, assessed by in situ zymography, were decreased in Lenti-*Slc44a2* mice after Ang II treatment (Figure 3H). The suprarenal abdominal aorta from Ang II–infused mice displayed reduced expression of a contractile marker (ACTA2) and elevated expression of a synthetic marker (OPN) in VSMCs, indicating VSMC dedifferentiation. In contrast, this dedifferentiation was inhibited in Lenti-*Slc44a2* mice (Figure 3I). qRT-PCR assay also indicated that SLC44A2 overexpression restored contractile transcript levels and inhibited synthetic markers in aortas from Ang II–infused mice (Supplemental Figure 3E). Taken together, these findings demonstrate that SLC44A2 suppresses Ang II–induced medial degeneration and restores the integrity of the arterial wall, thus protecting against aortic aneurysm.

### VSMC-specific SLC44A2 deficiency aggravates the development of Ang II–induced aortic aneurysm.

**Figure 4:**
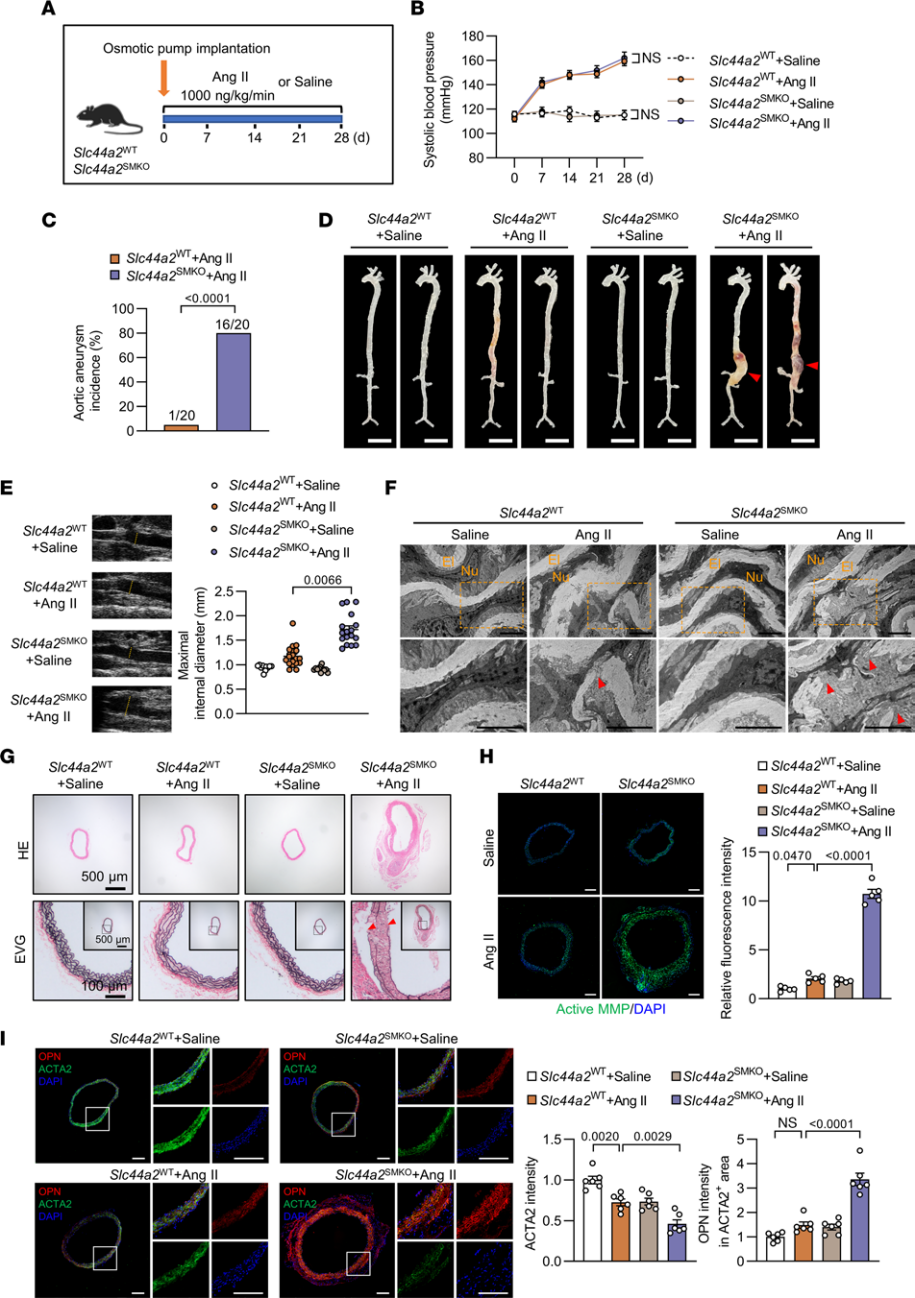
SLC44A2 knockout in VSMCs aggravates aortic aneurysm in Ang II–infused mice. (**A**) Eight- to 10-week-old male *Slc44a2*^WT^ and *Slc44a2*^SMKO^ mice were infused with saline or Ang II (1,000 ng/kg/min) for 28 days by osmotic pumps. (**B**) The systolic blood pressure of *Slc44a2*^WT^ and *Slc44a2*^SMKO^ mice at 0, 7, 14, 21, and 28 days after osmotic pump implantation. *n* = 11–20. NS, no significance. (**C**) The incidence of aortic aneurysm in Ang II–infused mice. *n* = 20. (**D**) Representative morphology of aortas from saline- or Ang II–infused mice. Scale bars: 5 mm. *n* = 11–20. (**E**) Ultrasound images and inner diameter quantification of the suprarenal abdominal aorta. *n* = 11–20. (**F**) Electron microscopic images of the suprarenal abdominal aorta. Red arrowheads indicate elastin breaks. El, elastin; Nu, nucleus. Scale bars: 5 μm. *n* = 3. (**G**) H&E and EVG staining of the suprarenal abdominal aorta. Red arrowheads indicate elastin breaks. *n* = 5. (**H**) Immunofluorescence images of in situ zymography (DQ gelatin, green) in the suprarenal abdominal aorta. Scale bars: 200 μm. *n* = 5. (**I**) Immunofluorescent staining for OPN (red), ACTA2 (green), and staining with DAPI (blue) in the suprarenal abdominal aorta. Scale bars: 200 μm. *n* = 6. Differences were analyzed by 2-way ANOVA with mixed effects followed by Tukey’s multiple-comparison test (**B**), Fisher’s exact test (**C**), Kruskal-Wallis test followed by Dunn’s multiple-comparison test (**E**), or 1-way ANOVA followed by Tukey’s multiple-comparison test (**H** and **I**).

Besides gain-of-function, a loss-of-function approach was undertaken using mice lacking SLC44A2 in VSMCs (*Slc44a2*^SMKO^) (Figure 4A). Successful ablation of SLC44A2 was demonstrated by SLC44A2 detection via Western blotting (Supplemental Figure 4A). Body weight and systolic blood pressure were comparable between the *Slc44a2*^WT^ and *Slc44a2*^SMKO^ groups (Supplemental Figure 4B and Figure 4B). As expected, aortic aneurysm incidence was much higher in Ang II–infused *Slc44a2*^SMKO^ mice (Figure 4C). SLC44A2-deficient mice were susceptible to aortic dilation and exhibited severe aneurysm (Figure 4D). The aortic rupture rate was 25% in Ang II–infused *Slc44a2*^SMKO^ mice, while no ruptures were recorded in *Slc44a2*^WT^ mice (Supplemental Figure 4C). In vivo ultrasound showed that *Slc44a2*^SMKO^ mice exhibited larger maximal internal diameters than WT in response to Ang II (Figure 4E). Additionally, the suprarenal abdominal aortas of *Slc44a2*^SMKO^ mice that received Ang II were characterized by more severe disruption of medial architecture, with prominent elastin degradation (Figure 4, F and G). Importantly, markedly enhanced MMP activities were seen in suprarenal abdominal aortas of *Slc44a2*^SMKO^ mice (Figure 4H). Immunofluorescence, qRT-PCR, and Western blot analysis of aortic tissue from *Slc44a2*^SMKO^ mice revealed a shift in VSMCs toward a synthetic phenotype (Figure 4I and Supplemental Figure 4, D and E). These data substantiate that VSMC-specific SLC44A2 deficiency accelerates the development and severity of aortic aneurysm.

Furthermore, we observed that the SLC44A2 deficiency induced VSMC phenotypic switching, evidenced by significantly reduced ACTA2 expression, increased OPN expression, and enhanced MMP activity, occurred 7 days after Ang II infusion, which is before the appearance of overt pathology (Supplemental Figure 5, A–D). This suggested the compensatory effect of SLC44A2 on maintaining the contractile phenotype of VSMCs during the development of aortic aneurysm.

### SLC44A2 preserves VSMC contractile phenotype through TGF-β/SMAD signaling via NRP1.

**Figure 5:**
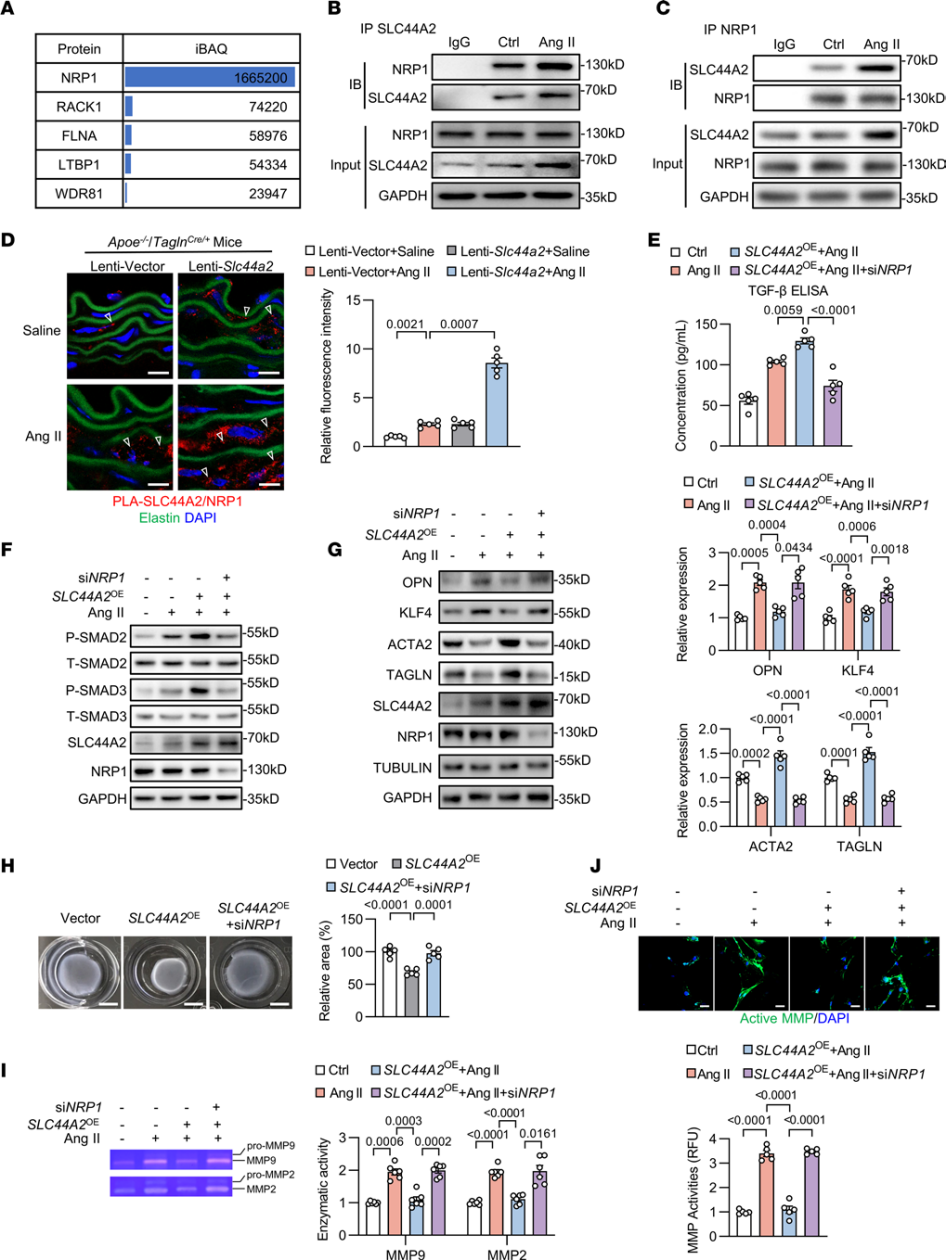
SLC44A2 activates TGF-β signaling to maintain the VSMC contractile phenotype via NRP1. (**A**) Lysates from HASMCs were immunoprecipitated with anti-SLC44A2 antibody followed by mass spectrometry analysis to identify the proteins that interact with SLC44A2. The graph shows the TGF-β signaling–related proteins. iBAQ, intensity-based absolute quantification. (**B**) HASMCs were treated with Ang II (1 μM). Lysates were immunoprecipitated with anti-SLC44A2 antibody, and blotted with anti-NRP1 and anti-SLC44A2 antibodies. *n* = 5. (**C**) HASMCs were treated with Ang II. Lysates were immunoprecipitated with anti-NRP1 antibody, and blotted with anti-SLC44A2 and anti-NRP1 antibodies. *n* = 4. (**D**) *Apoe^–/–^* *Tagln^Cre/+^* mice were intravenously injected with lentivirus containing control vector or *Slc44a2*. Osmotic pumps were then implanted subcutaneously to infuse saline or Ang II. The interaction of SLC44A2 with NRP1 (red dots marked by arrowheads) in suprarenal abdominal aorta was detected by proximity ligation assay (PLA). Scale bars: 10 μm. *n* = 5. (**E**–**J**) HASMCs were infected with lentivirus containing empty vector or SLC44A2-encoding plasmids with or without si*NRP1* transfection, and then treated with Ang II. (**E**) The TGF-β levels in culture medium was measured by ELISA. *n* = 5. (**F**) The levels of p-SMAD2 and p-SMAD3 were detected by Western blotting. *n* = 4. (**G**) The levels of VSMC synthetic and contractile markers were detected by Western blotting. *n* = 5. (**H**) Contraction of HASMCs grown in collagen discs was assessed and quantified by gel area. Scale bars: 5 mm. *n* = 5. (**I**) The activity of MMP2 and MMP9 in culture medium was measured by gel zymography. *n* = 6. (**J**) Immunofluorescence images of in situ zymography (DQ gelatin) in HASMCs. MMP activity was quantified by immunofluorescence intensity. Scale bars: 40 μm. *n* = 5. Differences were analyzed by Welch’s ANOVA followed by Tamhane’s T2 multiple-comparison test (**D** and **I**), 1-way ANOVA followed by Tukey’s multiple-comparison test (**E**, **H**, and **J**), or 1-way ANOVA followed by Tukey’s multiple-comparison test or Welch’s ANOVA followed by Tamhane’s T2 multiple-comparison test (**G**).

To investigate the potential mechanisms underlying SLC44A2-related VSMC phenotypic switching, we performed coimmunoprecipitation assay combined with mass spectrometry to scan potential downstream effectors. We showed that SLC44A2 could interact with several proteins related to TGF-β signaling and displayed the most abundant interaction with NRP1 (Figure 5A). TGF-β signaling plays a vital role in VSMC reprogramming, where VSMC-specific ablation of TGF-β signaling in *Apoe^–/–^* mice drives aneurysm formation (13). Although the enhanced TGF-β level was observed in both patients and mice with aortic aneurysm, direct evidence shows that blocking TGF-β by neutralizing antibody accelerates the development of aortic pathology in an Ang II– or elastase-induced aortic aneurysm mouse model (14–16). And consistently, TGF-β overexpression by endovascular gene therapy stabilized existing aortic aneurysms in a xenotransplantation model (17). Emerging scRNA-seq analysis further supports the notion that the adaptive activation of TGF-β signaling in VSMCs accounts for maintaining aortic homeostasis and preventing aortic aneurysm (18).

We next assessed the effect of SLC44A2-NRP1 interactions on TGF-β signaling. Coimmunoprecipitation assay demonstrated the interaction of SLC44A2 with NRP1 in HASMCs and Ang II stimulation enhanced their association (Figure 5, B and C). Proximity ligation assay (PLA) reveals close association of proteins (<40 nm), and foci of SLC44A2-NRP1 signals were increased within the vascular media of Ang II–treated *Apoe^–/–^* mice. The affinity of SLC44A2 for NRP1 was further increased in the Lenti-*Slc44a2* group (Figure 5D). Given NRP1’s role in the activation of the inactive latent form of TGF-β that is bound to latency-associated peptide (LAP-TGF-β) (19), we explored whether SLC44A2 participates in the regulation of TGF-β signaling by detecting the TGF-β level in the medium of HASMCs. Indeed, SLC44A2 overexpression could further increase the TGF-β concentration (Supplemental Figure 6A) and the level of p-SMAD2/3 upon Ang II treatment (Supplemental Figure 6B). The nuclear translocation of SMAD2 was increased by SLC44A2 overexpression in Ang II–treated HASMCs (Supplemental Figure 6, C and D). The elevated serum TGF-β concentration and p-SMAD2 were further confirmed in Ang II–infused *Apoe^–/–^* mice by Lenti-*Slc44A2* infection (Supplemental Figure 6, E and F). Inversely, SLC44A2 knockdown inhibited the elevated TGF-β level and p-SMAD2/3 triggered by Ang II (Supplemental Figure 6, G and H).

To further verify the notion that the positive effect of SLC44A2 on TGF-β activation is dependent on NRP1, we silenced NRP1 and assessed the consequences caused by SLC44A2 overexpression. The result showed that NRP1 knockdown inhibited SLC44A2-mediated increases in TGF-β and SMAD2/3 phosphorylation (Figure 5, E and F). Furthermore, SLC44A2 overexpression reduced the elevated synthetic markers and restored the reduced contractile markers induced by Ang II, the effect of which was abolished by NRP1 knockdown (Figure 5G). The enhanced contractility of VSMCs by SLC44A2 overexpression was also nullified after NRP1 silencing (Figure 5H). Gel zymography and in situ zymography assay showed that NRP1 knockdown eliminated the protective effect of SLC44A2 overexpression against increased MMP activities triggered by Ang II (Figure 5, I and J). Collectively, these results demonstrate that the prevention of VSMC phenotypic switching by SLC44A2 is NRP1 dependent.

### VSMC contractile phenotype depends on the SLC44A2-NRP1-ITGB3 complex.

**Figure 6:**
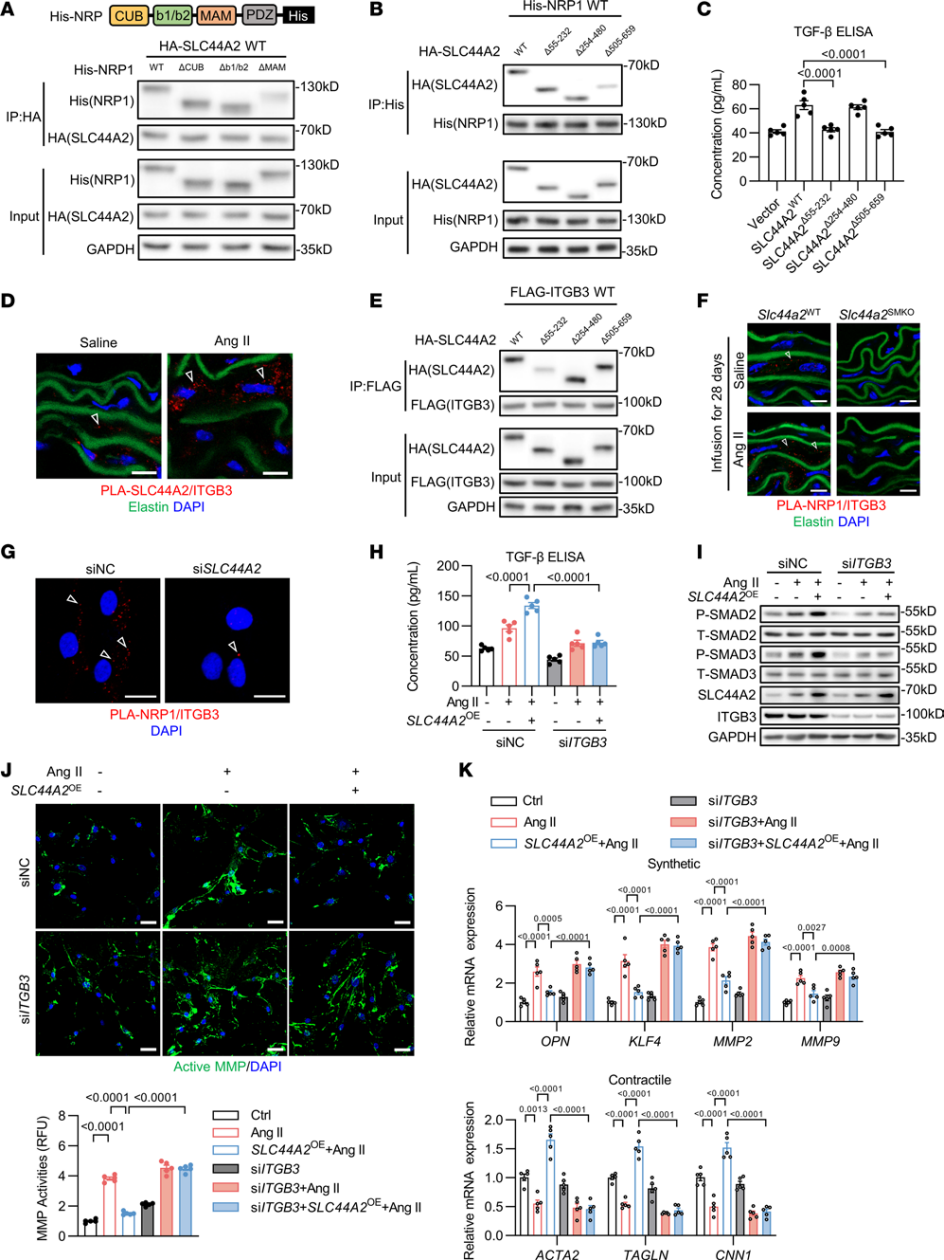
SLC44A2 mediates the activation of TGF-β by interacting with NRP1 and ITGB3. (**A**) HEK293 cells were transfected with SLC44A2^WT^ and plasmids encoding NRP1^WT^, NRP1^ΔCUB^, NRP1^Δb1/b2^, and NRP1^ΔMAM^. Lysates were immunoprecipitated with anti-HA antibody, and blotted with anti-His and anti-HA antibodies. *n* = 3. (**B**) HEK293 cells were transfected with NRP1^WT^ and plasmids encoding SLC44A2^WT^, SLC44A2^Δ55–232^, SLC44A2^Δ254–480^, or SLC44A2^Δ505–659^. Lysates were immunoprecipitated with anti-His antibody, and blotted with anti-His and anti-HA antibodies. *n* = 3. (**C**) MASMCs from *Slc44a2*^KO^ mice were infected with lentivirus containing empty vector or SLC44A2-encoding plasmids. The TGF-β level in culture medium was measured by ELISA. *n* = 5. (**D**) The interaction of SLC44A2 with ITGB3 in suprarenal abdominal aorta from *Apoe^–/–^* mice was detected by PLA. Scale bars: 10 μm. *n* = 3. (**E**) HEK293 cells were transfected with ITGB3^WT^ and plasmids encoding SLC44A2^WT^, SLC44A2^Δ55–232^, SLC44A2^Δ254–480^, or SLC44A2^Δ505–659^. Lysates were immunoprecipitated with anti-FLAG antibody, and blotted with anti-FLAG and anti-HA antibodies. *n* = 3. (**F**) The interaction of NRP1 with ITGB3 in suprarenal abdominal aortas from *Slc44a2*^WT^ and *Slc44a2*^SMKO^ mice was detected by PLA. Scale bars: 10 μm. *n* = 5. (**G**) The interaction of NRP1 with ITGB3 was detected in si*SLC44A2*-transfected HASMCs by PLA. Scale bars: 20 μm. *n* = 3. (**H**–**K**) HASMCs were infected with lentivirus containing empty vector or SLC44A2-encoding plasmids with or without si*ITGB3* transfection, and then treated with Ang II. (**H**) The TGF-β level in culture medium was measured by ELISA. *n* = 5. (**I**) p-SMAD2 and p-SMAD3 levels were detected by Western blotting. *n* = 3. (**J**) Immunofluorescence images and quantification of in situ zymography (DQ gelatin). Scale bars: 40 μm. *n* = 5. (**K**) VSMC synthetic and contractile markers were detected by qRT-PCR. *n* = 5. Differences were analyzed by 1-way ANOVA followed by Tukey’s multiple-comparison test (**C**, **H**, **J**, and **K**).

To better understand the underlying molecular mechanism, we constructed plasmids of His-tagged WT-NRP1 (NRP1^WT^) and CUB-, b1/b2-, or MAM-domain-deleted NRP1 (NRP1^ΔCUB^, NRP1^Δb1/b2^, or NRP1^ΔMAM^). These plasmids were cotransfected with plasmids of HA-tagged WT SLC44A2 in HEK293 cells, and coimmunoprecipitation assay showed that NRP1 MAM domain deletion inhibited the interaction of SLC44A2 with NRP1 (Figure 6A). Meanwhile, we found that deletion of residues 505–659 in SLC44A2 limited the interaction of SLC44A2 with NRP1 (Figure 6B). These results suggest that the MAM domain of NRP1 and residues 505–659 of SLC44A2 mediate their association. Then, we expressed WT SLC44A2 or 3 mutants of SLC44A2 with 3 different peptides deleted in *Slc44a2*^KO^ MASMCs to detect the TGF-β level. Strikingly, deletion of residues 55–232 or 505–659 decreased the TGF-β concentration (Figure 6C), suggesting a latent molecular mechanism for TGF-β activation. The arginine 154 (R154) in residues 55–232 of SLC44A2 is crucial for its binding to ITGB3 (20), a protein known to facilitate the cleavage of LAP, thereby activating latent TGF-β (21). In addition, ITGB3 is highly expressed in blood vessels (22). We speculated that residues 55–232 of SLC44A2 are essential for binding ITGB3 to mediate TGF-β activation. The binding of ITGB3 to SLC44A2 was confirmed by coimmunoprecipitation assay (Supplemental Figure 7A), and the high-affinity binding between SLC44A2 and ITGB3 was observed in the vascular media of Ang II–infused *Apoe^–/–^* mice by PLA (Figure 6D). Meanwhile, the deletion of residues 55–232 disrupted the association between SLC44A2 and ITGB3 (Figure 6E). SLC44A2 is able to recognize and bind to chaperones containing the VWF-A domain (7, 23). We showed that deletion of the VWF-A domain of ITGB3 inhibited the interaction between SLC44A2 and ITGB3 (Supplemental Figure 7B). Furthermore, SLC44A2 deficiency in VSMCs disrupted the interaction between NRP1 and ITGB3 in the suprarenal abdominal aorta infused with Ang II for 3, 7, and 28 days (Figure 6F and Supplemental Figure 7, C and D). In vitro experiments consistently showed that SLC44A2 knockdown inhibited the interaction between NRP1 and ITGB3 (Figure 6G and Supplemental Figure 7E), suggesting that SLC44A2 acts as a scaffold protein, binding both ITGB3 and NRP1. This was validated by GST pull-down assay using GST-tagged SLC44A2 and lysates from HASMCs and MASMCs, confirming SLC44A2 interacts with NRP1 and ITGB3 in vitro (Supplemental Figure 7, F and G).

To further substantiate our findings, we assessed the TGF-β/SMAD signaling after ITGB3 silencing. We found that ITGB3 knockdown abolished the effect of the SLC44A2 overexpression–induced increase in medium TGF-β concentration and p-SMAD2/3 level under Ang II treatment (Figure 6, H and I). Meanwhile, the suppression of MMP activities and the inhibition of phenotypic switching caused by SLC44A2 overexpression were nullified after ITGB3 knockdown (Figure 6, J and K). To confirm the dependence of SLC44A2’s effect on TGF-β, we employed a TGF-β–neutralizing antibody. It showed that blocking TGF-β reversed the protective effects of SLC44A2 on MMP activation and VSMC phenotypic switching upon Ang II treatment (Supplemental Figure 8, A and B). These data collectively demonstrate the crucial role of SLC44A2 in maintaining VSMCs’ contractile phenotype by interacting with NRP1 and ITGB3 to activate TGF-β signaling.

### RUNX1 regulates SLC44A2 transcription.

**Figure 7:**
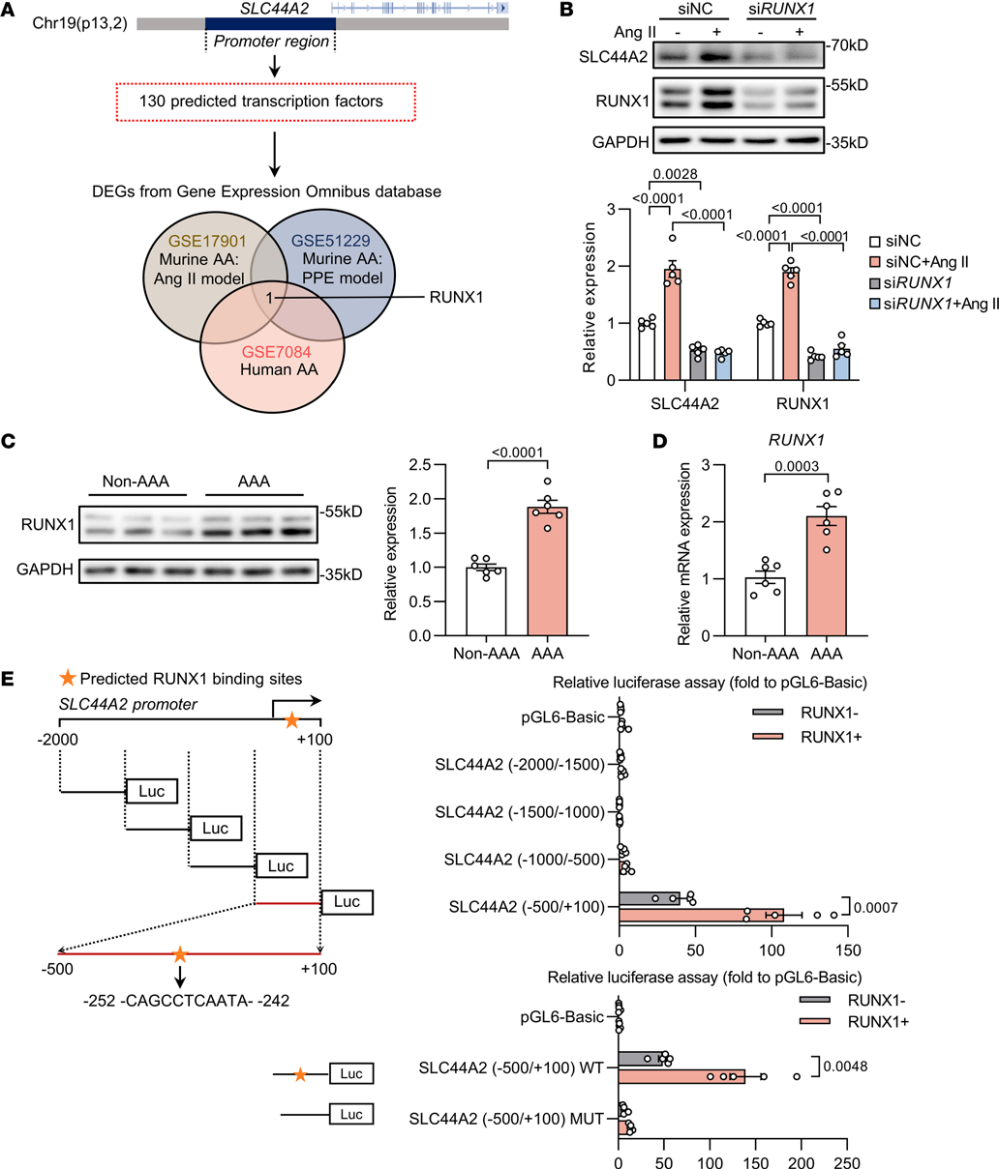
The transcription of SLC44A2 is regulated by RUNX1. (**A**) Prediction of *SLC44A2* promoter–binding transcription factors by JASPAR (https://jaspar.elixir.no/) and with the upstream 2,000 bp to downstream 100 bp region of *SLC44A2* gene transcription initiation site set as the promoter region. Venn diagram of DEGs in murine (GSE17901 and GSE51229) and human (GSE7084) aortic aneurysm samples relative to normal controls from the NCBI GEO database. AA, aortic aneurysm; PPE, porcine pancreatic elastase. (**B**) HASMCs were transfected with si*RUNX1* or siNC, and then treated with Ang II (1 μM, 24 hours). The levels of SLC44A2 and RUNX1 were detected by Western blotting. *n* = 5. (**C**) Western blot analysis of RUNX1 in the aortas of non-AAA and AAA individuals. *n* = 6. (**D**) *RUNX1* mRNA level in the aortas of non-AAA and AAA individuals was detected by qRT-PCR. *n* = 6. (**E**) Relative luciferase activity in HEK293 cells transfected with luciferase reporter constructs containing *SLC44A2* promoter truncations or its mutants along with pRL-TK (internal control plasmid) followed by transfection with RUNX1-encoding plasmid. *n* = 5. Differences were analyzed by 1-way ANOVA followed by Tukey’s multiple-comparison test (**B**), unpaired, 2-tailed Student’s *t* test (**C** and **D**), or unpaired, 2-tailed Student’s *t* test or Welch’s *t* test (**E**).

To unravel the molecular basis of SLC44A2 upregulation in aortic aneurysm, we integrated the prediction of *SLC44A2* promoter–binding transcription factors with digital gene expression (DGE) analysis of aortic RNAs from human and murine aortic aneurysm samples. RUNX1 was selected by inter-section analysis on recruited mRNA expression profiles (GSE17901, GSE51229, and GSE7084) and identified as a core regulator of SLC44A2 (Figure 7A). Treatment of HASMCs with RUNX1 siRNA resulted in a significant decrease in SLC44A2 levels (Figure 7B), Notably, the RUNX1 level was elevated in aortic samples from patients with AAA (Figure 7, C and D). Luciferase assays showed that RUNX1-dependent *SLC44A2* activation was maintained upon transfection with luciferase vector containing the –500 bp to +100 bp sequence of the *SLC44A2* promoter (Figure 7E). Mutations in the predicted binding sites (–252 bp to –242 bp) completely abrogated the effect of RUNX1 on *SLC44A2* promoter activation, indicating this region’s significance in transcription induction by RUNX1 (Figure 7E). Taken together, these results show that upregulation of RUNX1 in VSMCs accounts for the induction of SLC44A2 during aortic aneurysm.

To validate RUNX1’s binding to the *SLC44A2* promoter, we performed EMSA assays using nuclear extracts from HASMCs and synthesized biotin-labeled oligonucleotides encompassing RUNX1 binding sites on the *SLC44A2* promoter. The observed protein binding (lane 2) was competed out by unlabeled probe (lane 3), and the binding signal was blocked by RUNX1 antibody (lane 4), but not by control IgG (lane 5) (Supplemental Figure 9A). ChIP assay was performed to pull down RUNX1 and followed by qRT-PCR to amplify sequences containing the RUNX1 binding site from –252 to –242 (CAGCCTCAATA) in HASMCs. The result showed the binding of RUNX1 to the *SLC44A2* promoter sequence under physiological conditions, which was enhanced by Ang II treatment (Supplemental Figure 9B). Notably, RUNX1 overexpression further promoted the binding of RUNX1 to the *SLC44A2* promoter sequence (Supplemental Figure 9B). Furthermore, Western blot analysis showed that overexpressing RUNX1 significantly enhanced SLC44A2 expression in HASMCs (Supplemental Figure 9C).

Parallel results were obtained in MASMCs by EMSA, ChIP, and Western blot assay (Supplemental Figure 9, D–F). These results provide evidence that RUNX1 directly binds to the *SLC44A2* promoter to regulate its expression both in MASMCs and HASMCs.

### LEN may act as an effective activator of RUNX1 to enhance SLC44A2 expression, inhibiting VSMC phenotypic switching.

We next investigated the role of RUNX1 in regulating VSMC phenotypic switching. As shown in Supplemental Figure 10, A–D, RUNX1 knockdown exacerbated Ang II–induced VSMC phenotypic switching, but this was prevented in RUNX1-overexpressing VSMCs. LEN, which has demonstrated clinical efficacy in multiple myeloma and striking activity in myelodysplastic syndrome (MDS), can upregulate RUNX1 in hematopoietic stem and progenitor cells (24). Since SLC44A2 transcription is modulated by RUNX1, we next evaluated the effect of LEN on VSMC phenotypic switching. We observed that both RUNX1 and SLC44A2 levels began to increase modestly following stimulation with 5 μM LEN and reached sustainable higher levels at concentrations of 10–40 μM (Supplemental Figure 11A). Consistent with expectations, LEN upregulated SLC44A2 expression upon Ang II treatment (Supplemental Figure 11B). Notably, LEN-induced SLC44A2 expression was negated by RUNX1 knockdown (Supplemental Figure 11C), indicating that RUNX1 may be one of the major factors involved in LEN’s effect on SLC44A2 expression.

LEN treatment significantly alleviated Ang II–induced MMP activation and contractile phenotype loss, the effects of which were normalized by SLC44A2 knockdown (Supplemental Figure 11, D–F). Meanwhile, LEN-promoted TGF-β secretion was blunted by SLC44A2 silencing (Supplemental Figure 11G). These results together suggest that LEN inhibits VSMC phenotypic switching through inducing SLC44A2 expression.

### Supplementation with LEN suppresses aortic aneurysm initiation.

**Figure 8:**
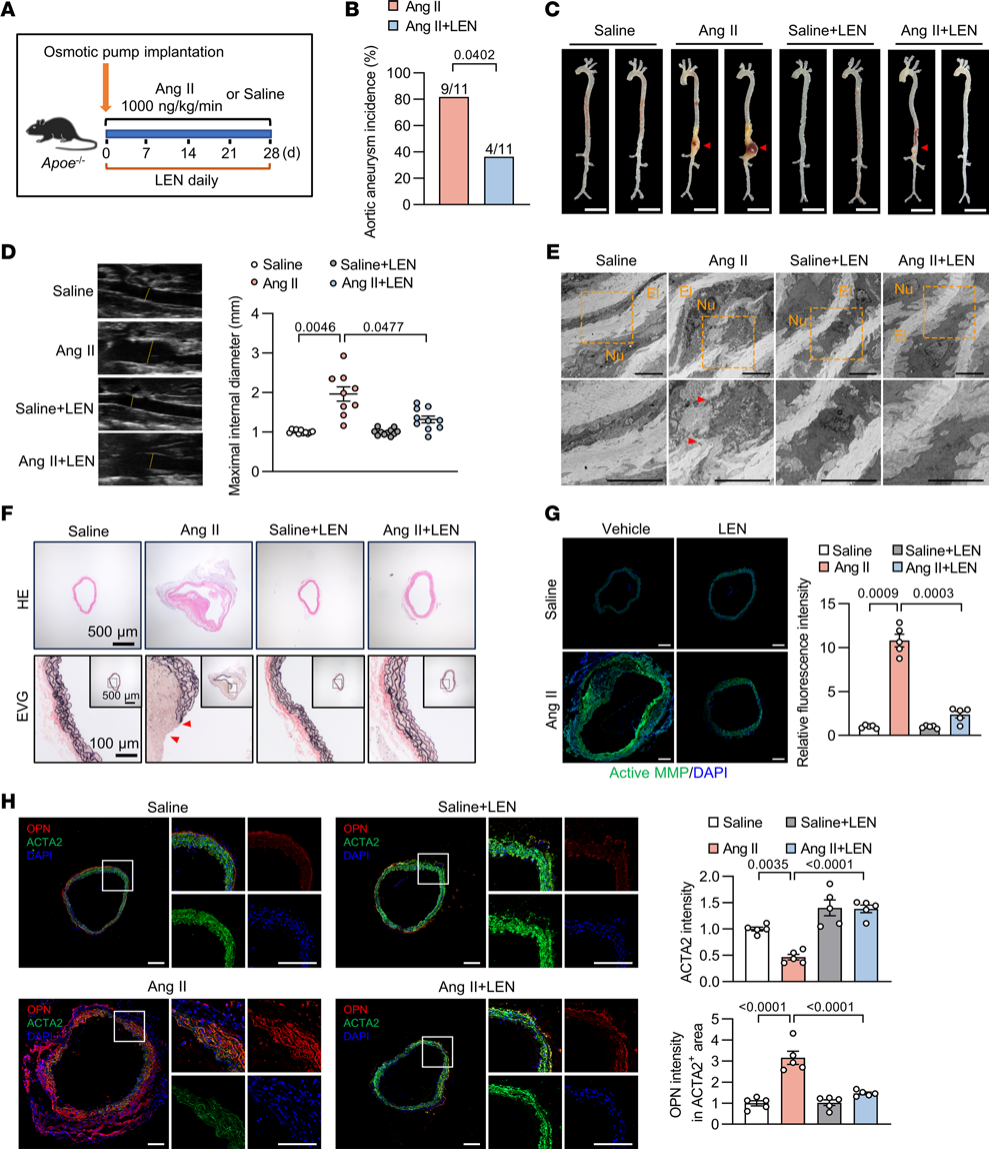
Administration of LEN relieves aortic aneurysm in mice. (**A**) Eight- to 10-week-old male *Apoe^–/–^* mice were implanted subcutaneously with osmotic pumps to infuse saline or Ang II (1,000 ng/kg/min) with or without intragastric administration of LEN (20 mg/kg/day) for 28 days. (**B**) The incidence of aortic aneurysm in Ang II–infused *Apoe^–/–^* mice administered vehicle or LEN. *n* = 11. (**C**) Representative morphology of aortas from Ang II–infused *Apoe^–/–^* mice administered vehicle or LEN. Scale bars: 5 mm. *n* = 11. (**D**) Ultrasound images and inner diameter quantification of the suprarenal abdominal aorta. *n* = 9–11. (**E**) Electron microscopic images of the suprarenal abdominal aorta. Red arrowheads indicate elastin breaks. El, elastin; Nu, nucleus. Scale bars: 5 μm. *n* = 3. (**F**) H&E and EVG staining of the suprarenal abdominal aorta. Red arrowheads indicate elastin breaks. *n* = 5. (**G**) Immunofluorescence images of in situ zymography (DQ gelatin, green) in the suprarenal abdominal aorta. Scale bars: 200 μm. *n* = 5. (**H**) Immunofluorescent staining for OPN (red), ACTA2 (green), and staining with DAPI (blue) in the suprarenal abdominal aorta. Scale bars: 200 μm. *n* = 5. Differences were analyzed by Fisher’s exact test (**B**), Welch’s ANOVA followed by Tamhane’s T2 multiple-comparison test (**D** and **G**), or 1-way ANOVA followed by Tukey’s multiple-comparison test (**H**).

To assess LEN’s in vivo efficacy, we administered LEN daily for 28 days in the aortic aneurysm model induced by Ang II infusion (Figure 8A). LEN did not overtly affect body weight (Supplemental Figure 12A) and systolic blood pressure (Supplemental Figure 12B). As reported, the most common adverse reactions of LEN at high doses are hematological adverse reactions, including neutropenia, thrombocytopenia, and anemia (25). Therefore, we examined the effects of LEN on in vivo hematopoiesis. Administration of LEN did not induce myelosuppression (Supplemental Table 2), consistent with prior work showing that LEN does not cause a decline in peripheral blood counts in WT mice (26). Additionally, LEN showed no side effects on metabolic parameters and hepatorenal function (Supplemental Table 3). Compared with the vehicle group, LEN-treated mice exhibited lower aortic aneurysm incidence after Ang II infusion (Figure 8B), along with attenuated aortic dilation (Figure 8C). The aortic rupture rate in the vehicle group was 27.27%, while it was 9.09% in LEN-treated mice after Ang II infusion (Supplemental Figure 12C). Diameters of the suprarenal abdominal aorta were progressively increased after Ang II infusion, whereas LEN treatment inhibited their enlargement (Figure 8D). Accordingly, transmural medial breaks were improved in LEN-treated groups (Figure 8, E and F), concomitant with the reduced MMP activities and the mitigated VSMC dedifferentiation in aortic tissues (Figure 8, G and H, and Supplemental Figure 12D). Of note, costaining of ACTA2 and SLC44A2 showed a significantly upregulated SLC44A2 level in the media layer after LEN administration (Supplemental Figure 12E). PLA signals for SLC44A2-NRP1-ITGB3 association were increased after LEN administration (Supplemental Figure 12, F and G). Moreover, we observed elevated serum TGF-β levels and increased aortic SMAD2 phosphorylation under LEN treatment (Supplemental Figure 12, H and I). Taken together, these data demonstrate that LEN activates TGF-β/SMAD signaling via SLC44A2 to prevent aortic aneurysm.

Finally, to verify the specific mechanism of LEN in vivo, we administered LEN to Ang II–infused *Slc44a2*^SMKO^ mice. The results showed that the protective effect of LEN was totally abolished by SLC44A2 deficiency, as evidenced by unimproved aortic aneurysm incidence, aortic diameter expansion, aortic rupture rate, elastin breakage, and MMP activation in Ang II–infused *Slc44a2*^SMKO^ mice treated with LEN (Supplemental Figure 13, A–H). These results conclusively demonstrate that the effect of LEN is dependent on SLC44A2.

## Discussion

**Figure 9:**
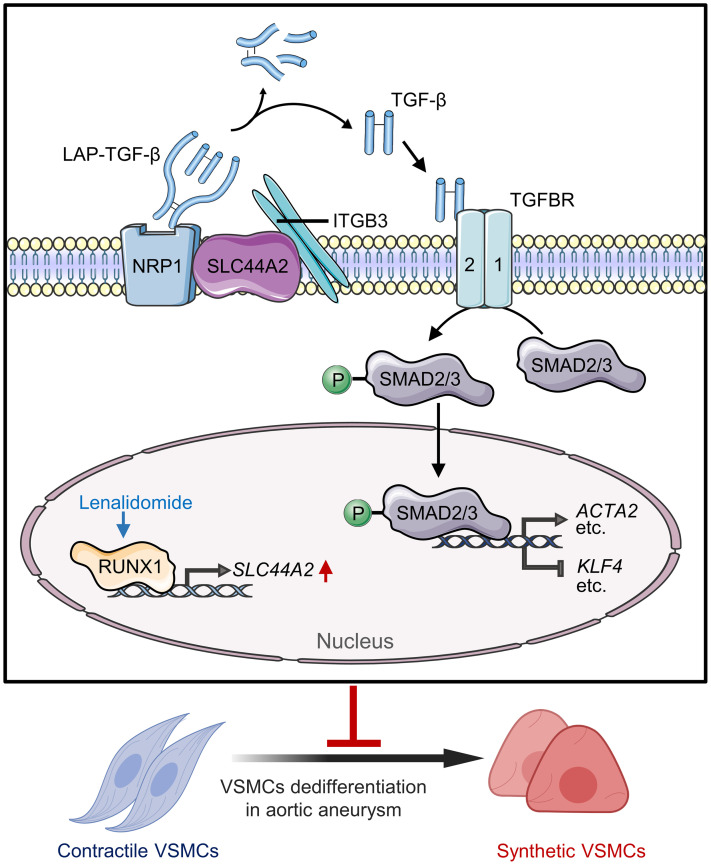
Proposed model for SLC44A2 as a therapeutic target in aortic aneurysm. Lenalidomide promotes RUNX1-mediated transcription of SLC44A2. The upregulated SLC44A2 acts as a scaffolding protein to interact with NRP1 and ITGB3 to activate TGF-β/SMAD signaling, further promoting the expression of VSMC contractile genes and inhibiting the expression of VSMC synthetic genes to restrain the VSMC phenotypic switching in aortic aneurysm.

This study elucidates the critical role of SLC44A2 in regulating VSMC phenotypic switching and its implication in aortic aneurysm development. First, SLC44A2 was adaptively augmented in aortic aneurysm lesions from humans and mice. Overexpression of SLC44A2 in VSMCs mitigated the vascular remodeling of aortic aneurysm, while VSMC-specific knockout of SLC44A2 aggravated the development of aortic aneurysm in Ang II–infused mice. Moreover, mechanistic studies revealed that SLC44A2 mediated the interaction between NRP1 and ITGB3. The formation of the NRP1-SLC44A2-ITGB3 trimolecular complex contributed to the activation of TGF-β and subsequent TGF-β/SMAD pathway elicitation to maintain the contractile phenotype of VSMCs. Additionally, we demonstrated that the transcription factor RUNX1 could bind to the *SLC44A2* gene promoter to activate its transcription. Lastly, LEN may upregulate RUNX1 to increase SLC44A2 expression, ameliorating VSMC phenotypic switching and protecting against aortic aneurysm (Figure 9).

SLC44A2 deficiency has been associated with hair cell loss, spiral ganglion degeneration, and hearing loss in mice, and with Ménière disease and transfusion-related acute lung injury in humans (10, 23). Emerging data show that SLC44A2 is a thrombosis regulator, controlling mitochondrial energetics in platelet activation and production of neutrophil extracellular traps (20, 27). Genome-wide association studies have linked the expression of the human neutrophil antigen 3b epitope on the SLC44A2 protein with a 30% decreased risk of venous thrombosis (28). However, the precise role of SLC44A2 in cardiovascular disease is not well understood. Although SLC44A2 was upregulated in aortic aneurysm tissues from humans and mice, we found a profound suppressive effect of SLC44A2 on aortic aneurysm development. Notably, SLC44A2 was markedly increased in Ang II–induced aneurysmal mice, in parallel with the activation of TGF-β signaling, including TGF-β secretion and SMAD2/3 phosphorylation. Accumulating evidence highlights that upregulated TGF-β levels during aortic aneurysm may serve as an adaptive response to maintain aortic strength, and the blockade of TGF-β by neutralizing antibody could promote the development and rupture of aortic aneurysm in experimental aneurysm models (14, 16, 18). Similarly, a previous study documented that Ang II caused a significant increase in ADAM15 protein levels in the abdominal aorta, which could be an important compensatory mechanism that limits aortic aneurysm formation, whereas mice lacking ADAM15 developed aortic aneurysm (29). Another study reported that hepcidin expression was markedly increased in VSMCs within the aneurysm tissue, and mice with VSMC-specific deletion of hepcidin exhibited a heightened phenotype of aortic aneurysm (30). We demonstrated here that enhanced SLC44A2 expression acts as an adaptive program in VSMCs that attenuates VSMC dedifferentiation and protects against aortic aneurysm. By activating TGF-β signaling, SLC44A2 could effectively amplify the adaptive response in the aortic wall.

SLC44A2 is mainly localized at cellular and mitochondrial membranes, and functions by interacting with proteins containing VWF-A domains. SLC44A2 was initially reported as an antigen in the inner ear, essential for maintaining normal hearing through its interaction with cochlin (7). Furthermore, SLC44A2 can bind to von Willebrand factor (VWF), a key molecule in hemostasis, and this interaction on neutrophils leads to agglutination (23). Arg154Gln polymorphism of SLC44A2 results in a reduced binding of SLC44A2 to VWF (9). We identified NRP1 as a binding partner of SLC44A2 by mass spectrometry scanning. NRP1 knockdown abolished the protective effect of SLC44A2 overexpression against VSMC phenotypical switching. NRP1 is capable of binding to a broad repertoire of ligands, accounting for its diverse biological functions in immunity, tumorigenesis, and vascular development (31). NRP1 exerts pleiotropic roles in TGF-β signaling, where it activates TGF-β signaling in stromal fibroblast cell lines and breast cancer cells (32). Conversely, NRP1 suppresses the endothelial stalk-cell phenotype by limiting TGF-β/SMAD activation (33). NRP1 is a high-affinity receptor for the inactive membrane-bound latent form, LAP-TGF-β, in T cells (34), while the precise molecular mechanism underlying NRP1-induced latent TGF-β activation remains unclear.

It is well established that LAP-TGF-β activation occurs in an integrin-dependent manner (21). Integrins bind to arginine-glycine-aspartic acid (RGD) motifs in the LAP segment, which leads to a physical traction– and stress-mediated deformation of the latent complex, allowing for subsequent liberation of active TGF-β (35). Notably, integrin αvβ3 is abundant in blood vessels (22) and its regulation of Rho GTPase activation is involved in VSMC differentiation (36). SLC44A2 engagement with ITGB3 mediates adhesion of neutrophils to primed platelets (20), but the interaction between SLC44A2 and ITGB3 in VSMCs is yet to be elucidated. Our results demonstrate that SLC44A2 acts as a scaffold protein, assembling ITGB3 and NRP1, thereby activating the TGF-β/SMAD signaling pathway. We showed that the amino acid sequence 505–659 of SLC44A2 was essential for binding NRP1 with its MAM domain, while the amino acid sequence 55–232 of SLC44A2 was essential for binding ITGB3 with its VWF-A domain. These results provide the evidence for a regulatory SLC44A2/NRP1/ITGB3 signaling axis in TGF-β activation.

TGF-β signaling plays a vital but diverse role in aortic aneurysm and VSMC reprogramming (37). Some literature posits that the activation TGF-β signaling in VSMCs is the primary cause of Marfan and Loeys-Dietz syndromes (38, 39), where these inherited aortic aneurysm predispositions were initially proposed to be due to overactivity in the TGF-β pathway. However, most current evidence supports the reverse hypothesis that the TGF-β pathway protects against aortic aneurysm formation (40, 41). Indeed, pathogenic variants or deficiency of TGF-β pathway genes, including *TGFB2*, *TGFB3*, *TGFBR1*, *TGFBR2*, and *SMAD3*, confer risks for aortic destruction and thoracic aortic aneurysm formation (42). A combination of reduced TGF-β signaling and hypercholesterolemia drives ascending aortic aneurysm development (13). Meanwhile, anti–TGF-β blocking antibody has been used to augment aneurysm growth and induce intraluminal thrombus when establishing an aortic aneurysm mouse model (43). This was in agreement with a single study showing that adenovirus-mediated overexpression of TGF-β stabilized expanding aortic aneurysm in rats (17). When overexpressed in the heart and plasma, TGF-β1 is a vasculoprotective cytokine that prevents aortic dilation (44). Additionally, intramural delivery of TGF-β1 hydrogel can effectively decrease aneurysm progression in CaCl_2_-induced aortic aneurysm (45). Proefferocytic anti-CD47 antibody therapy promotes TGF-β signaling and prevents aneurysm formation (46). These data suggest that upregulating the TGF-β pathway is a legitimate therapeutic approach for aortic aneurysm, assuming effective and safe interventions can be developed.

TGF-β/SMAD signaling protects against aortic aneurysm by abrogation of VSMC phenotypic switching. Mutations in TGFBR2 compromise both basal and TGF-β–induced expression of contractile proteins in VSMCs (47). Loss of smooth muscle TGF-β signaling input, when combined with hyperlipidemia, results in a transdifferentiation of small population of VSMCs to a mesenchymal stem cell–like state (13). Our results consistently reveal that SLC44A2 maintains VSMC contractile gene transcription by activating TGF-β/SMAD signaling.

So far, the transcriptional regulation of SLC44A2 remains unclear. In our study, we identified RUNX1 as a regulator of SLC44A2 transcription. We found that the upregulated RUNX1 in VSMCs contributed to the compensating increase in SLC44A2 during aortic aneurysm. RUNX1 is a vital transcription factor in hematopoiesis and its dysregulation is intimately related to MDS (48). LEN is the first-line medication for MDS by virtue of its ability to reverse the cytologic and cytogenetic abnormalities by upregulating RUNX1 (24, 49). Our study indicated that RUNX1 may be one of the major factors involved in LEN’s effects on SLC44A2 expression. Use of LEN in vivo significantly reduced the incidence of aortic aneurysm. Moreover, compared with the dosage of LEN (50 mg/kg/day) for hematological disease (26), the application of low-dose LEN (20 mg/kg/day) showed an effective role in aortic aneurysm therapy in mice with no hematological adverse reactions, including neutropenia, thrombocytopenia, and anemia. This finding is consistent with thrombocytopenia seen in patients treated with LEN, which is often a dose-limiting toxicity (50, 51). Treatment with LEN may facilitate the clinical management of aortic aneurysm.

There are several limitations of this study. First, the long-axis ultrasonic imaging evaluates aortic diameter in the anterior-posterior direction and may result in underestimations of aortic diameter compared with short-axis measurement in both anterior-posterior and transverse directions (52). Second, MASMC isolation from the whole abdominal aorta has a limited ability in exhibiting the specific features of aortic aneurysm, given that aortic dilation in Ang II–infused mice occurs predominantly in the suprarenal abdominal aorta. Also, our study found that SLC44A2 expression was increased in aortas of patients with AAA, unveiling that SLC44A2 may be exploited as a promising target for therapy development against AAA. This direction deserves future confirmation studies using larger cohorts of patients with AAA. Our results revealed that LEN suppressed aortic aneurysm initiation in Ang II–induced mouse models, highlighting LEN supplementation as a potential treatment for AAA. Extending clinical research will be required to validate the efficacy and translational feasibility of LEN in AAA management. Finally, given the dose-limiting haematological adverse reactions of LEN, further studies are expected to elucidate the safety, tolerability, and pharmacokinetic profile in long-term clinical use for AAA.

In summary, we uncovered a crucial role of SLC44A2 in activating TGF-β/SMAD signaling by interacting with NRP1 and ITGB3, which is essential for VSMCs’ contractile phenotype maintenance. LEN inhibits VSMC phenotypic switching by modulating the RUNX1/SLC44A2 axis and improves the aortic pathology in mouse aortic aneurysm models, revealing a potential prospect for clinical application in humans.

## Methods

### Sex as a biological variable.

Our study exclusively examined male mice because aortic aneurysm is a sexually dimorphic disease and aortic aneurysm exhibits lower female prevalence (1, 53). It is unknown whether the findings are relevant for female mice.

Detailed descriptions of experimental methods of the current study are provided in Supplemental Methods.

### scRNA-seq and analysis.

Analysis of scRNA-seq data was performed according to a previously reported method (54). Cells with a mitochondria ratio of greater than 20% and fewer than 500 genes were filtered out. Integrative cell clustering results were visualized as a 2-dimensional UMAP plot and read counts were natural-log normalized (each transcript counts/total counts × 10,000) using the “NormalizeData” function for each cell. Following single-cell clustering and annotation of each cell state cluster, we predominantly focused on *Slc44a2* expression patterns across 8 cell lineages, encompassing VSMCs, fibroblasts, endothelial cells, macrophages, T cells, B cells, erythrocytes, and dendritic cells.

### Human tissue sample acquisition and preparation.

Two types of human abdominal aortic tissues were used for this study: AAA samples (*n* = 6) and non-AAA samples (*n* = 6). The diagnosis of AAA was confirmed by computed tomographic angiography. Abdominal aortic tissues were collected during surgical repair for AAA. Non-AAA samples were obtained from organ donor controls.

As previously described (55–57), aortic segments were freshly isolated in the operating theatre, with AAA tissue harvested at the maximal dilation. The aortic tissue was then processed as follows. Both non-AAA and AAA aortic samples were placed in ice-cold physiological salt solution immediately upon removal, followed by stripping of the periaortic tissue and mural thrombus. The aortic tissue was divided into several segments, which were either fixed in 4% paraformaldehyde (PFA) for histologic analyses or snap-frozen in liquid nitrogen followed by storage at –80°C for RNA or protein extraction. The entire process from aorta collection to tissue processing and storing was completed within 3 hours.

### Animal studies.

*Apoe*^–/–^ mice were purchased from GemPharmatech Co. Ltd. and *Tagln^Cre/+^* mice were purchased from the Model Animal Research Center of Nanjing University. *Slc44a2*^KO^ and *Slc44a2^fl/fl^* mice were originally generated by the Animal Center of Nanjing Medical University. For specific ablation of SLC44A2 in VSMCs (*Slc44a2*^SMKO^), *Slc44a2^fl/fl^* mice were crossed with *Tagln^Cre/+^* mice. *Apoe*^–/–^ mice were crossed with *Tagln^Cre/+^* mice to generate *Apoe*^–/–^ *Tagln^Cre/+^* mice. PCR primers (5′–3′) used for genotyping were as follows: TCGCAGAATTACTACGGGAAGC and GGGTGACCAGCTGATTCATCAG (*Slc44a2^fl/fl^* mice); TGCCACGACCAAGTGACAGCAATG and ACCAGAGACGGAAATCCATCGCTC (*Tagln^Cre/+^* mice). All experimental male mice (8 to 10 weeks old) were on a C57BL/6J background and maintained with free access to chow and water.

### Aortic aneurysm model.

Ang II–induced aneurysm model: 8- to 10-week-old male mice were infused with Ang II (1,000 ng/kg/min) or saline by osmotic pumps (Alzet model 2004, Alza Corp) for 28 days. Mice were anesthetized with inhaled isoflurane and the minipumps were surgically implanted into the subcutaneous space of the mice in the back of the neck.

Animals were regularly monitored and weighed. Aneurysm was defined as a 50% or greater increase in aortic diameter of the segment in unchallenged mice with the same genetic background.

### Abdominal ultrasonography in mice.

Aortic diameters were measured by high-frequency ultrasound Vevo 2100 echography device (VisualSonics). These measurements were performed on day 0, 14, and 28 after Ang II infusion. Mice were anesthetized using 2% isoflurane and placed on a supine position. The abdominal area was shaved and coated with ultrasound transmission gel before positioning the acquisition probe. As previously described (58–60), long-axis ultrasound scans of suprarenal aortas were performed from the aortic hiatus to the renal artery. The probe was first applied on the short axis to locate the abdominal aorta, adjacent to the inferior vena cava. The abdominal aorta was then centered and the probe was switched to the long axis. Color mode was activated to help localize the 2 renal arteries, displaying a red blood flow toward the probe on the screen. The probe was then adjusted to capture the area of the maximum dilation in the suprarenal abdominal aorta. Maximal internal diameters of aortic images were measured with Vevo LAB software.

### Lentivirus-mediated overexpression.

Mouse *Slc44a2* cDNA was amplified by PCR and cloned into the pLVX-FLEX-EF1a-ZsGreen lentiviral vector, which was provided by Jingjing Ben (Department of Pathophysiology, Nanjing Medical University, Nanjing, China). The correct sequence of the *Slc44a2* gene in this construct was verified by sequencing. We cloned the expression cassette in an inverse, antisense orientation between 2 different *loxP* sites. The construct was designed so that Cre induction could be used to mediate the inversion of the *Slc44a2* cassette into a sense orientation. Control or *Slc44a2* lentivirus (1 × 10^9^ TU per mouse) was injected into *Apoe*^–/–^ *Tagln^Cre/+^* mice via tail vein. In these mice, Cre induction mediated the initial flipping of the cassette, contingent on the orientation and location of 2 *loxP* sites.

### Drug administration.

Stock solution of LEN was prepared in dimethyl sulfoxide (DMSO), stored at –80°C, and diluted with sterile saline before intragastric administration. To examine its preventive effects against Ang II–induced aortic aneurysm, *Apoe*^–/–^ mice were administered LEN via oral gavage concurrent with the 28-day Ang II infusion period.

### Transmission electron microscopy.

Suprarenal abdominal aortas were dissected and sliced into small fragments of 3–5 mm each. Aortic slices were then fixed in 5% glutaraldehyde for 1–2 days. Specimens were postfixed in 1% osmium tetroxide. After en bloc staining with 2% aqueous uranyl acetate for 2 hours, samples were dehydrated in a series of ethanol up to 100% and embedded in epoxy resin. Ultrathin sections were cut with an EM UC7 ultramicrotome (Leica) and poststained with lead nitrate. Sample grids were observed under a JEM-1400Flash Electron Microscope (JEOL).

### MMP activity determined by in situ zymography and gel zymography.

For in situ zymography, MMP activity was determined by an EnzChek Gelatinase/Collagenase assay kit (E12055, Thermo Fisher Scientific) following the manufacturer’s protocol. The isolated suprarenal abdominal aortic tissue was embedded into OCT solution and rapidly frozen with dry ice. Freshly cut frozen aortic sections or HASMCs were incubated with a fluorogenic gelatin substrate (DQ gelatin, D12054, Thermo Fisher Scientific) at a concentration of 25 μg/mL at 37°C for 24 hours, while being protected from light. The fluorescence of DQ gelatin is quenched until MMP-catalyzed hydrolysis occurs. The resulting fluorescence intensity is directly proportional to proteolytic digestion. Negative control zymograms were incubated in the presence of the MMP inhibitor 1,10-phenanthroline (10 mM). The samples were then fixed in 4% PFA and stained with DAPI. Proteolytic activity was detected as green fluorescence (at 495 nm absorption/515 nm emission) by confocal microscopy (Zeiss LSM 800).

For gel zymography, supernatants from cultured HASMCs were harvested and centrifuged by Amicon Ultra Centrifugal Filter (UFC801096, Millipore) to yield concentrated conditioned media, as previously described (61). The conditioned media were then subjected to 10% sodium dodecyl sulfate polyacrylamide gel electrophoresis (SDS-PAGE) polymerized in the presence of 1 mg/mL gelatin as a substrate for MMP activity. After electrophoresis, gels were washed 3 times with 2.5% Triton X-100 to remove SDS, and then incubated for 48 hours (37°C) in developing buffer (50 mM Tris-HCl [pH 7.4], 150 mM NaCl, 5 mM CaCl_2_, and 0.1% Brij-35). The gels were then stained with Coomassie Brilliant Blue and destained to reveal clear bands indicating zones of gelatinolytic activity.

### PLA.

PLA was performed with Duolink reagents (DUO92101, Sigma-Aldrich) following the manufacturer’s instructions. Suprarenal abdominal aortic cryosections or cells were fixed with 4% PFA and permeabilized in the same manner as the standard immunostaining procedure (62), followed by blocking with Duolink blocking buffer for 1 hour. Primary antibodies from 2 different species were then incubated overnight at 4°C. After washing, exact PLUS and MINUS probes conjugated with secondary antibodies were added and hybridized for 1 hour at 37°C. Ligation, rolling circle amplification, and detection with fluorescent probes were performed. PLA signals, recognized as red fluorescent dots, were visualized and images were captured using confocal microscopy (Zeiss LSM 800). The primary antibodies used were mouse anti-SLC44A2 (sc-101266, Santa Cruz Biotechnology), rabbit anti-NRP1 (ab81321, Abcam), mouse anti-NRP1 (sc-5307, Santa Cruz Biotechnology), and rabbit anti-ITGB3 (18309, Proteintech). Negative controls were performed using only 1 primary antibody.

### Cell culture.

HASMCs (6110, Sciencell) were propagated in Smooth Muscle Cell Medium (1101, Sciencell) with 2% fetal bovine serum (FBS), Smooth Muscle Cell Growth Supplement (SMCGS), and 100 U/mL penicillin/streptomycin at 37°C with 5% CO_2_.

HEK293 cells purchased from American Type Culture Collection were maintained in Dulbecco’s modified Eagle’s medium (DMEM, Thermo Fisher Scientific) or DMEM/F12 (Thermo Fisher Scientific) with 10% FBS (Gibco).

Confluent cells (80%–85%) were rested in serum-free medium for 24 hours and then treated with Ang II (1 μM) at the specified times, followed by subsequent experiments (transfection, etc.).

### Isolation of MASMCs.

MASMCs were isolated from the whole aortas or the whole abdominal aortas of saline-infused mice or Ang II–infused aortic aneurysm mouse models by enzymatic digestion, as described previously (63, 64). Briefly, mice were dissected until the thoracic cavity was exposed, and then perfused with cold sterile PBS. The aorta was cleaned from surrounding tissues, followed by rinsing the aorta in cold sterile PBS. Then adventitia and endothelium were gently removed, and the aorta was cut into 1–2 mm explants. The finely cut tissues were digested in collagenase type II (LS004177, Worthington Biochemical Corporation) at 37°C with 5% CO_2_ in an incubator for 3–4 hours. Afterwards, the same amount of FBS was added to stop the enzymatic reaction. Cell pellets were collected by centrifuging at 2,000*g* for 10 minutes and supernatants were discarded as much as possible. Cells were resuspended with DMEM supplemented with 10% FBS and 100 U/mL penicillin/streptomycin. MASMCs were identified based on immunofluorescent staining of ACTA2.

### Collagen gel contraction assay.

HASMCs were treated with siRNA or lentivirus and cultured in serum-free medium for 24 hours prior to being seeded into collagen gels, as previously described (65). Collagen gels were prepared by mixing HASMCs with type I collagen (A1048301, Thermo Fisher Scientific), 2× DMEM, 1 M NaOH, and distilled water. The mixture was seeded in 24-well cell culture plates and incubated at 37°C for 30 minutes. Polymerized gels were then dislodged from the well by gentle mechanical force. Digital photographs of collagen gel lattices were taken after 24 hours, and the gel surface area was measured by ImageJ (NIH).

### Statistics.

All values are presented in the figures as mean ± SEM, with a *P* value of less than 0.05 considered statistically significant. The *n* values in figure legends represent biological replicates or the number of mice and human samples. For statistical comparisons, we first evaluated whether data were normally distributed using the Shapiro-Wilk normality test. Nonparametric tests were used when data were not normally distributed. For 2-group parametric tests, the Levene test was applied to assess the equality of variances. Significant difference between 2 groups was determined by unpaired, 2-tailed Student’s *t* test when data showed equal variance; otherwise, *t* test assuming unequal variance was performed. For comparisons among more than 2 groups, the Brown-Forsythe test was used to evaluate homogeneity of variance. For comparing the differences between different groups, 1-way ANOVA or Welch’s ANOVA was applied for equal variances assumed or not, respectively. Two-way ANOVA with mixed effects was used for comparing the parameters that were repeatedly measured over time, including body weight, blood pressure, and inner diameters of suprarenal abdominal aorta of the mice at 0 to 28 days after osmotic pump implantation. All graphs were generated and statistical analyses were performed using GraphPad Prism 8.

### Study approval.

The use of human aortic tissue was approved by the medical ethics committee of Nanjing Drum Tower Hospital and the ethics committee of Nanjing Medical University following the Declaration of Helsinki. Written informed consent was provided by all participants or the organ donors’ legal representatives before enrollment. All animal experiments were conducted in accordance with the ARRIVE guidelines for the care and use of laboratory animals, and with approval of the Nanjing Medical University Animal Care and Use Committee.

### Data availability.

All supporting data are available within the article and Supplemental Methods. Values for all data points in graphs are reported in the Supporting Data Values file. scRNA-seq data are available in the NCBI GEO database under accession number GSE152583 (12).

## Author contributions

YJ, LX, and YH developed the concept, designed the study, and revised the manuscript. TS and SZ analyzed the data and drafted the manuscript. TS, SZ, SL, and CC performed the experiments. XL, XW, JC, ZW, and YW provided technical assistance. ZS reanalyzed RNA-seq data sets. XD and XL provided clinical samples. ZH, HC, FC, LW, HW, KS, BY, and ZZ supervised the in vivo and in vitro study. The order of co–first authors was determined by the volume of work each contributed to the study.

## Supplementary Material

Supplemental data

Unedited blot and gel images

Supporting data values

## Version 1 06/25/2024

In-Press Preview

## Version 2 08/15/2024

Electronic publication
