# Supplementary material for: SLC44A2 regulates vascular smooth muscle cell phenotypic switching and aortic aneurysm
Source: J Clin Invest. 2024 Jun 25;134(16):e173690. doi: 10.1172/JCI173690 (PMC11324303; doi:10.1172/JCI173690)
Supplement: Supplemental data [file jci-134-173690-s012.pdf]

Supplemental Material

SLC44A2 regulates vascular smooth muscle cell phenotypic switching and aortic aneurysm

Supplemental Tables

Supplemental table 1. The primer sequences used for quantitative real-time polymerase chain reaction (qRT-PCR) analysis were described

Gene	Forward (5' to 3' sequence)	Reverse (5' to 3' sequence)	Species
SLC44A2	TGGAGGACGAGCGGAAAAAC	TATCCGTGCAGCCCCTATTG	Human
OPN	ATCTCCTAGCCCCACAGACC	CACACTATCACCTCGGCCAT	Human
KLF4	GATGCTCACCCCACCTTCTT	TCATCTGAGCGGGCGAATTT	Human
MMP2	ACCAGCTGGCCTAGTGATGA	CCGCATGGTCTCGATGGTAT	Human
MMP9	TCTATGGTCCTCGCCCTGAA	CATCGTCCACCGGACTCAAA	Human
ACTA2	AAAGCAAGTCCTCCAGCGTT	TAGTCCCGGGGATAGGCAAA	Human
TAGLN	GGAAACCCACCCTCTCAGTC	TGCACTAGCCAAGTCATCCG	Human
CNN1	GAGGTTAAGAACAAGCTGGCCC	CGTTGGCCTCAAAAATGTCGT	Human
RUNX1	GGACGCCAGAAGGAAGTCAA	TCGGACCACAGAGCACTTTC	Human
Slc44a2	GATGGCCCTAGGATGTCTGC	GCTGACTTGCCCACTCTCAT	Mouse
Uchl1	AGTGGCCAACAACCAAGACA	TTGGCTCTATCTTCGGGGGA	Mouse
Dkk3	GTTCGTAGCAGTGTGGTGGA	CGGTGTGACTGAGAAAGGCT	Mouse
Anxa3	TAGAGGAAGGTCTCCTGCCC	ACCTCGATACCTCACCTGCT	Mouse
Cryab	GGCGCCTCCAAGTTAAGGAT	ATCAGGGATTTGGCAGGGTG	Mouse
Opn	AATCTCCTTGCGCCACAGAA	GGACATCGACTGTAGGGACG	Mouse
Klf4	TGGCCATCGGACCTACTTATC	CATGTCAGACTCGCCAGGTG	Mouse
Mmp2	CCACGGGCCCTATCATCTTC	CAGCACCTTTCTTTGGGCAC	Mouse
Mmp9	TTCACCGGCTAAACCACCTC	TAACGCCCAGTAGAGAGCCT	Mouse
Acta2	CGCCTCCAGTTCCTTTCCAA	AGAGGGGGCCACCCTATAAT	Mouse
Tagln	AGGGGTGACATCACTGCCTA	GACTGCACTTCTCGGCTCAT	Mouse
Cnn1	GGGTTACGGTTTGGGGAGAT	AACTCAGTGCTTCCTTCGGG	Mouse

Supplemental table 2. Peripheral blood counts of Ang II-infused aortic aneurysm mice, which were given a 20 mg/kg/day dose of LEN for 28 days

	Saline	Ang II	Saline+LEN	Ang II+LEN	Multiple comparison	P values
WBC (10^9/L)	5.62±0.35	5.07±0.36	5.30±0.43	5.17±0.49	Ang II vs Saline	0.7773
					Ang II+LEN vs Ang II	0.9980
RBC (10^{12}/L)	9.02±0.76	9.06±0.61	8.86±0.53	8.92±0.65	Ang II vs Saline	0.9999
					Ang II+LEN vs Ang II	0.9986
PLT (10^9/L)	749.80±91.11	766.40±94.96	766.00±69.23	803.40±69.28	Ang II vs Saline	0.9989
					Ang II+LEN vs Ang II	0.9883
NEU (10^9/L)	1.73±0.06	1.88±0.20	1.80±0.05	1.83±0.15	Ang II vs Saline	0.8604
					Ang II+LEN vs Ang II	0.9921

WBC, white blood cell; RBC, red blood cell; PLT, platelet; NEU, neutrophils. Data are expressed as the mean ± SEM (n = 5 mice per group). The P values were calculated by one-way ANOVA.

Supplemental table 3. Biochemical parameters of Ang II-infused aortic aneurysm mice, which were given a 20 mg/kg/day dose of LEN for 28 days

	Saline	Ang II	Saline+LEN	Ang II+LEN	Multiple comparison	P values
TC (mmol/L)	14.61±0.87	15.17±1.47	16.26±0.97	15.76±1.54	Ang II vs Saline	0.9883
					Ang II+LEN vs Ang II	0.9865
TG (mmol/L)	1.51±0.11	1.45±0.13	1.58±0.27	1.50±0.14	Ang II vs Saline	0.9959
					Ang II+LEN vs Ang II	0.9980
HDL (mmol/L)	0.81±0.05	0.86±0.10	0.92±0.19	0.82±0.05	Ang II vs Saline	0.9844
					Ang II+LEN vs Ang II	0.9941
ALT (U/L)	30.80±2.65	27.20±2.22	28.20±2.99	28.00±3.08	Ang II vs Saline	0.7931
					Ang II+LEN vs Ang II	0.9968
AST (U/L)	135.20±8.94	128.20±5.98	121.60±8.16	123.60±13.67	Ang II vs Saline	0.9542
					Ang II+LEN vs Ang II	0.9861
ALB (g/L)	29.54±0.75	26.30±1.28	27.10±0.84	25.42±0.95	Ang II vs Saline	0.1288
					Ang II+LEN vs Ang II	0.9184
BUN (mmol/L)	10.12±0.47	10.42±0.89	9.18±0.66	9.80±0.87	Ang II vs Saline	0.9915
					Ang II+LEN vs Ang II	0.9331
CREA (μmol/L)	16.00±0.55	16.60±1.63	17.00±2.30	18.20±1.93	Ang II vs Saline	0.9946
					Ang II+LEN vs Ang II	0.9129
UA (μmol/L)	87.40±5.25	86.00±4.21	81.00±7.51	86.60±4.77	Ang II vs Saline	0.9979
					Ang II+LEN vs Ang II	0.9998

TC, total cholesterol; TG, triglyceride; HDL, high-density lipoprotein; ALT, alanine

transaminase; AST, aspartate aminotransferase; ALB, albumin; BUN, blood urea nitrogen; CREA, creatinine; UA, uric acid. Data are expressed as the mean ± SEM (n = 5 mice per group). The P values were calculated by one-way ANOVA.

Supplemental table 4. Biotin labeled SLC44A2 nucleotide probes for EMSA analysis

Primers	Position	Strand	Nucleotide sequence
Human	-257/-237	Sense<br>Anti-sense	5'-ACGCCCAGCCTCAATATCTCC-3'<br>5'-TGCGGGTCGGAGTTATAGAGG-3'
Mouse	-426/-406	Sense<br>Anti-sense	5'-TTCTGACTTGTGTTCAAACTC-3'<br>5'-AAGACTGAACACAAGTTTGAG-3

Supplemental table 5. The primer sequences used for ChIP analysis

Gene	Forward (5' to 3' sequence)	Reverse (5' to 3' sequence)	Species
SLC44A2	TCTCCTTGACGATGAAATGCATAGA	CTTGAGGGTGTGCATGGTGA	Human
Slc44a2	CCAGTGCTGGGATTGGAAGA	GGTACTGAGCGCTGACTGAG	Mouse

Supplemental Figures and Figure Legends

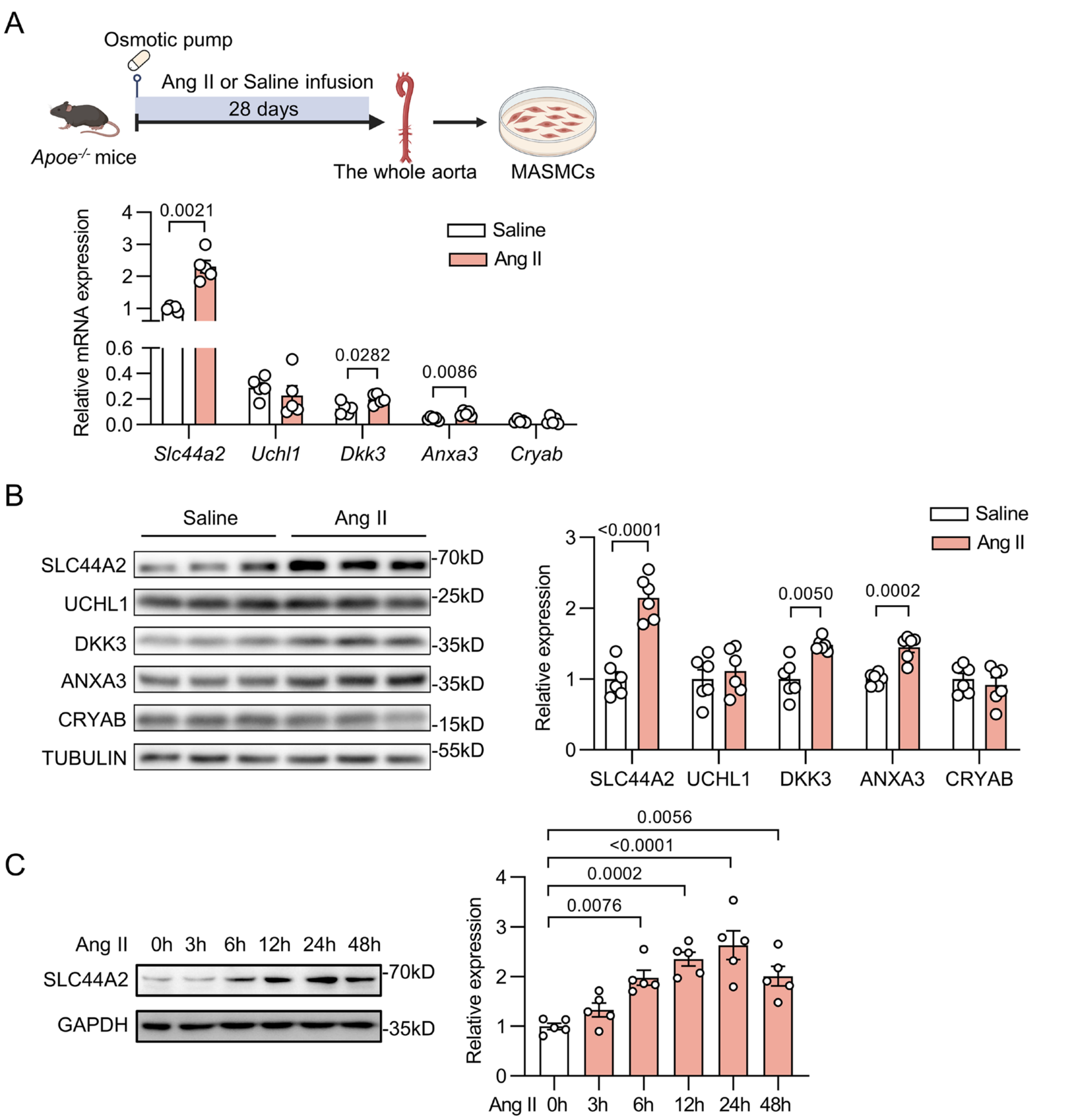

Supplemental Figure 1. SLC44A2 expression in MASMCs from Ang II-infused mice or in HASMCs treated by Ang II.
(**A**) MASMCs were isolated from the whole aortas of saline- or Ang II-infused mice. The mRNA levels of *Slc44a2*, *Uchl1*, *Dkk3*, *Anxa3*, and *Cryab* were detected by qRT-PCR. n = 5. (**B**) MASMCs were isolated from the whole abdominal aortas of saline- or Ang II-infused mice. The protein levels of SLC44A2, UCHL1, DKK3, ANXA3, and CRYAB were detected by Western blotting. n = 6. (**C**) Western blot analysis of SLC44A2 expression in HASMCs treated with Ang II (1 μM) for 3, 6, 12, 24, and 48 hours. n = 5. **A** and **B**, unpaired two-tailed *t*-test or Welch's *t* test; **C**, one-way ANOVA.

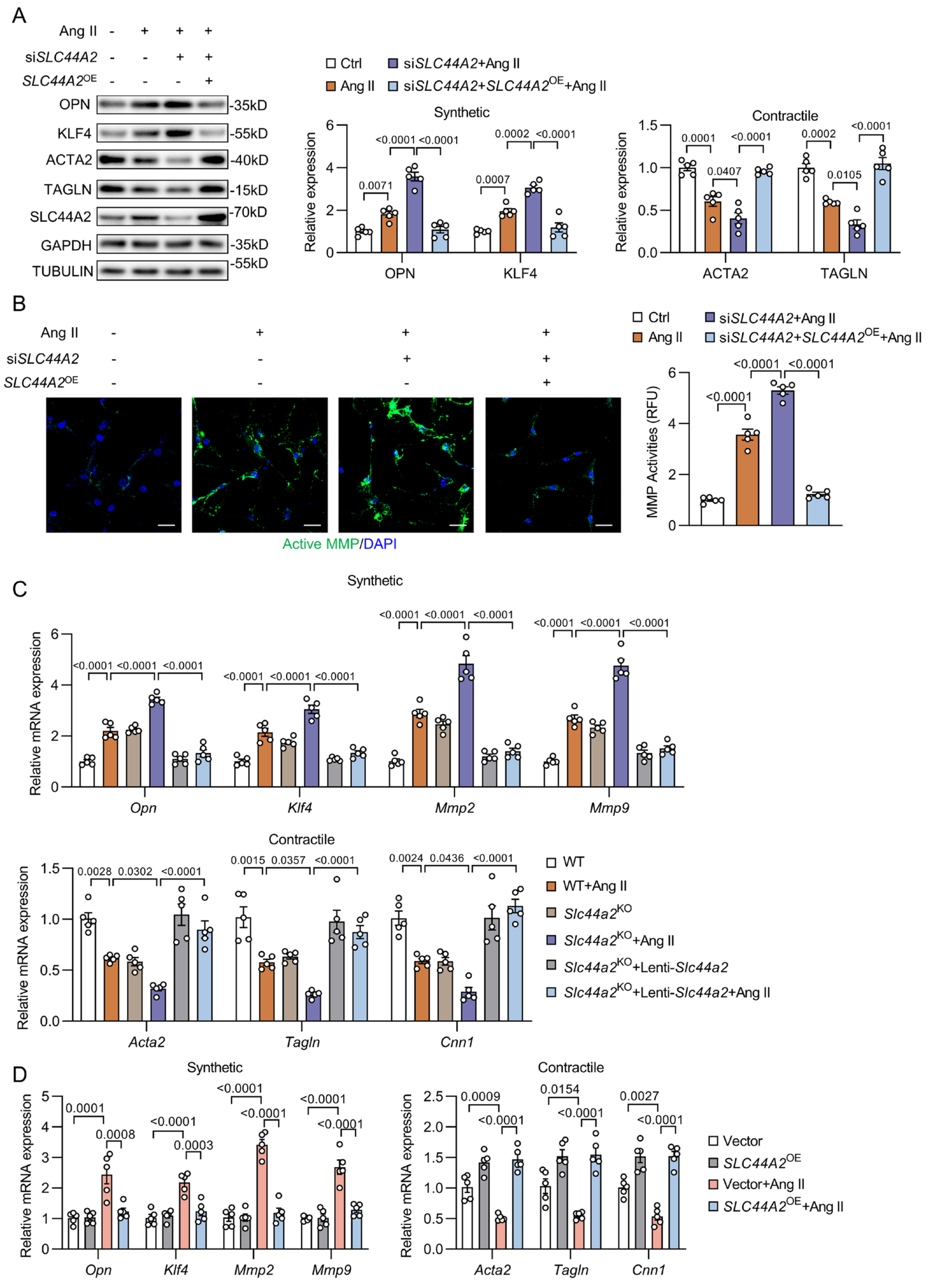

Supplemental Figure 2. SLC44A2 overexpression rescues phenotypic switching in SLC44A2 deficient VSMCs.

(**A** and **B**) HASMCs were transfected with SLC44A2 siRNA (si*SLC44A2*) against 3'UTR (Forward: CCATTGGGGCCTCTTGATGT; Reverse: GGTGTGGAGAAGGGGTTCAG) to silence endogenous SLC44A2 and infected with lentivirus to overexpress (OE) SLC44A2, followed by Ang II treatment (1 μM, 24 hours). (**A**) The synthetic and contractile markers were detected by Western blotting. n = 5. (**B**) Immunofluorescence images of in situ zymography (DQ gelatin). MMP activity

was quantified by immunofluorescence intensity. Scale bar, 40 μm. n = 5. (**C**) MASMCs isolated from the whole abdominal aorta of WT and *Slc44a2*^{KO} mice were infected with Lenti-Vector or Lenti-*Slc44a2*, then treated with vehicle or Ang II (1 μM, 24 hours). The VSMCs synthetic and contractile markers were detected by qRT-PCR. n = 5. (**D**) MASMCs isolated from the whole abdominal aorta of WT mice were infected with Lenti-Vector or Lenti-*Slc44a2*, and treated by vehicle or Ang II (1 μM, 24 hours). The VSMCs synthetic and contractile markers were detected by qRT-PCR. n = 5. **A-D**, one-way ANOVA.

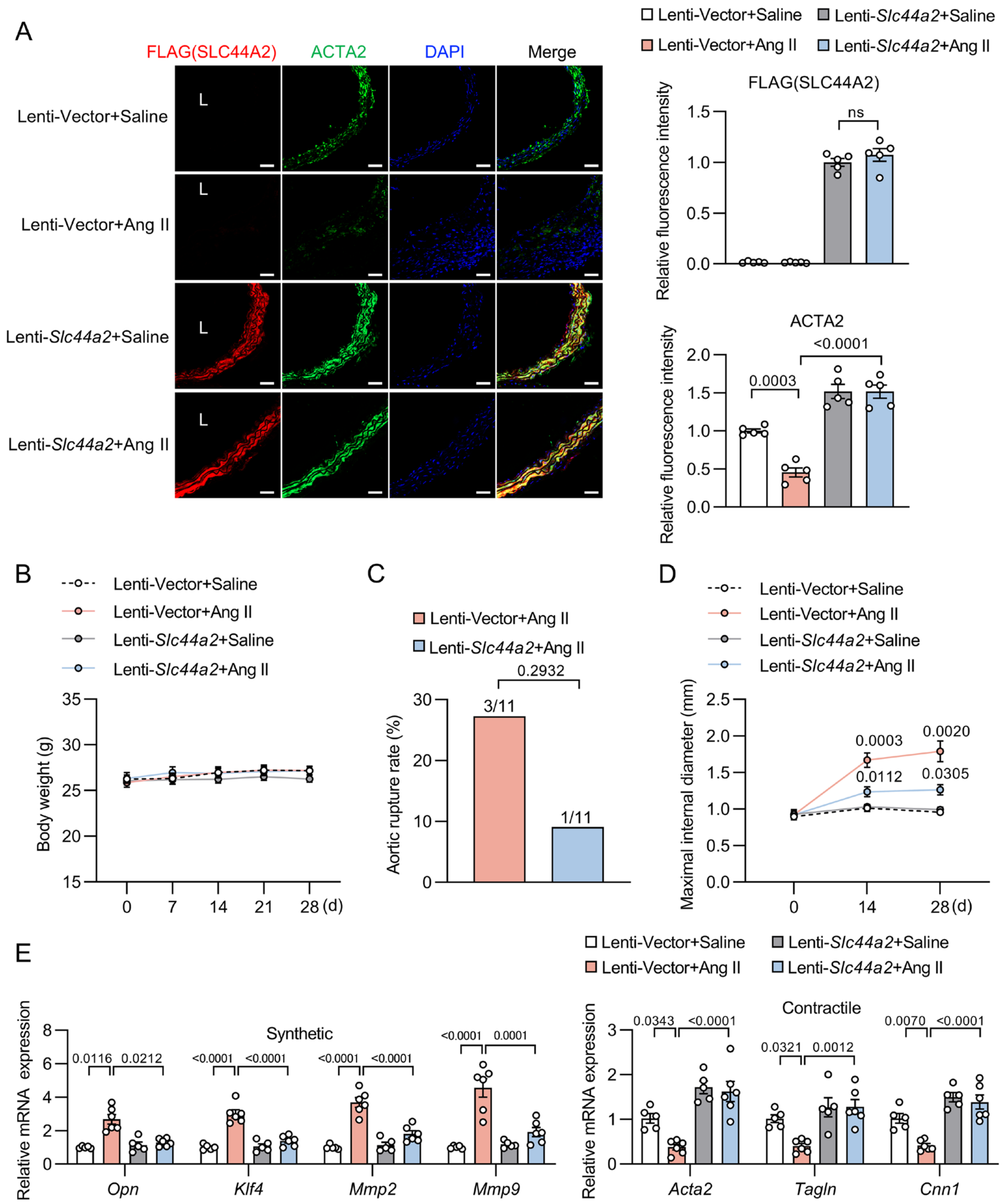

Supplemental Figure 3. SLC44A2 overexpression moderates VSMCs phenotypic switching in Ang II-infused *Apoe*$^{-/-}$ mice.

(**A-D**) Eight-week-old male *Apoe*$^{-/-}$/*Tagln*$^{Cre/+}$ mice were intravenously injected with

lentivirus containing control vector (Lenti-Vector) or reverse Slc44a2 sequence with two LoxP sites (Lenti-*Slc44a2*). After 2 weeks, osmotic pumps were implanted to infuse saline or Ang II (1000 ng/kg/min) for 28 days. (**A**) Immunofluorescence staining for FLAG(SLC44A2) (red), ACTA2 (green), and DAPI (blue) in the suprarenal abdominal aorta. L, lumen. Scale bar, 40 μm. n = 5. (**B**) The body weight at 0, 7, 14, 21, and 28 days after osmotic pumps implantation. n = 8-11. (**C**) The aortic rupture rate in Ang II-infused mice. n = 11. (**D**) Inner diameter of the suprarenal abdominal aorta was detected by ultrasound at 0, 14, and 28 days after osmotic pumps implantation. n = 8-11. (**E**) qRT-PCR analysis of VSMCs synthetic markers (*Opn*, *Klf4*, *Mmp2*, and *Mmp9*) and contractile markers (*Acta2*, *Tagln*, and *Cnn1*) in the whole aorta from saline- or Ang II-infused mice. n = 5-6. **A**, one-way ANOVA; **B** and **D**, two-way ANOVA with mixed-effects; **C**, Fisher's exact test; **E**, one-way ANOVA or Welch’s ANOVA.

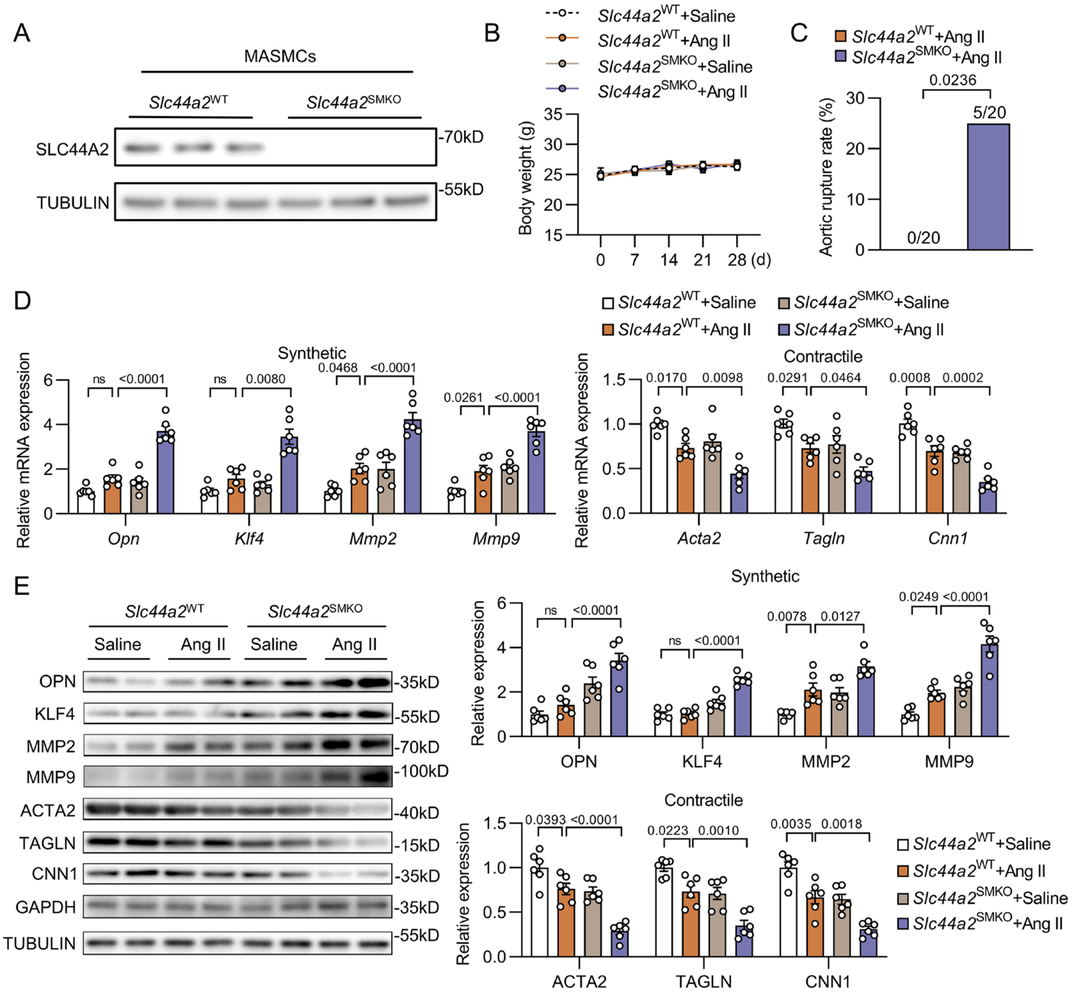

Supplemental Figure 4. SLC44A2 deficiency aggravates VSMCs phenotypic switching in Ang II-infused mice.

(**A**) Western blot analysis of SLC44A2 expression in the MASMCs from *Slc44a2*^{WT} and *Slc44a2*^{SMKO} mice. n = 3. (**B**) The body weight of *Slc44a2*^{WT} and *Slc44a2*^{SMKO} mice at 0, 7, 14, 21, and 28 days after osmotic pumps implantation. n = 11-20. (**C**) The aortic rupture rate in Ang II-infused mice. n = 20. (**D**) qRT-PCR analysis of VSMCs synthetic markers (*Opn*, *Klf4*, *Mmp2*, and *Mmp9*) and contractile markers (*Acta2*, *Tagln*, and *Cnn1*) in the whole aortas from saline- or Ang II-infused mice. n = 6. (**E**) Western blot analysis of VSMCs synthetic markers (OPN, KLF4, MMP2, and MMP9) and contractile markers (ACTA2, TAGLN, and CNN1) in the whole aortas from saline- or Ang II-infused mice. n = 6. **B**, two-way ANOVA with mixed-effects; **C**, Fisher's exact test; **D**, one-way ANOVA or Welch's ANOVA; **E**, one-way ANOVA.

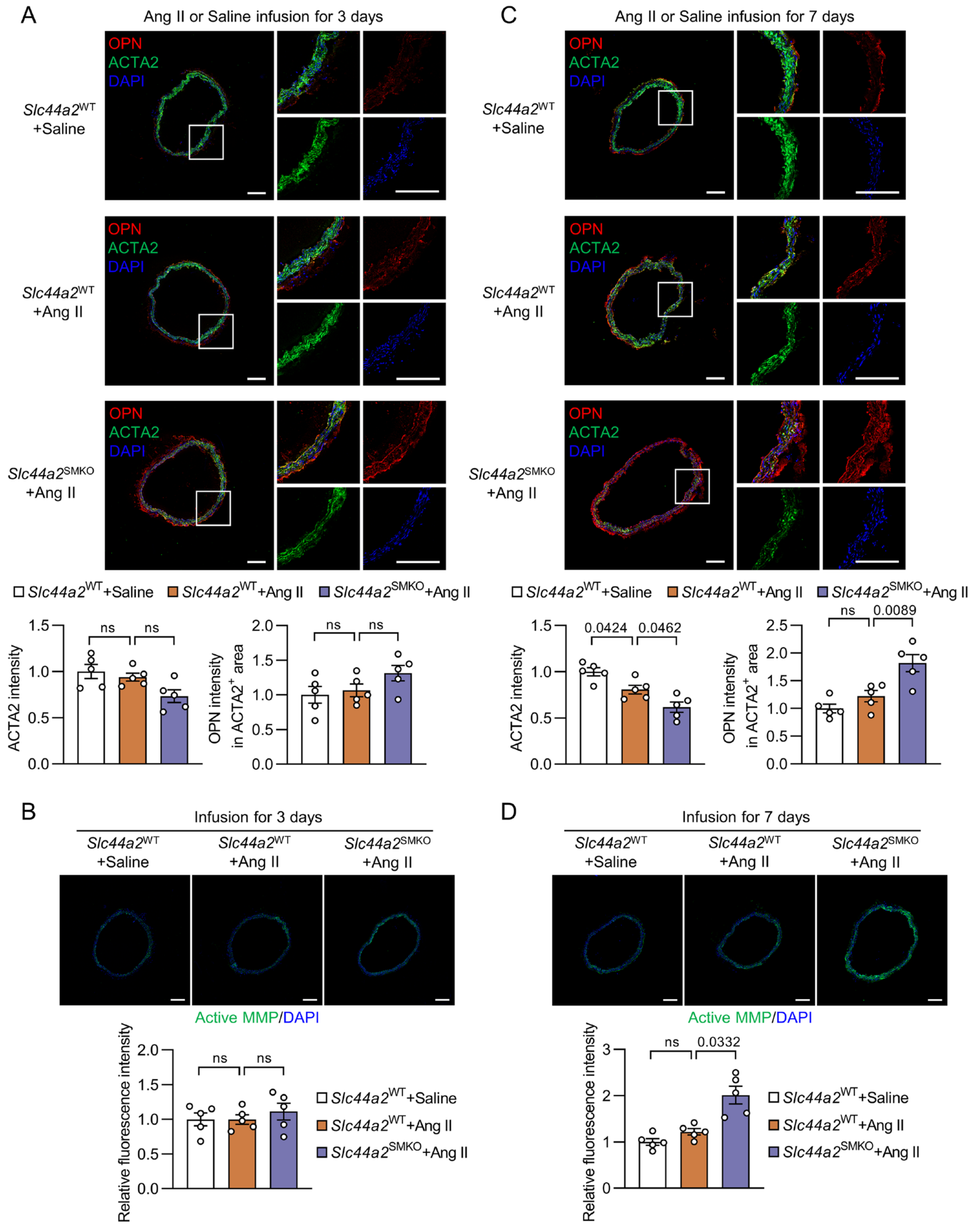

Supplemental Figure 5. SLC44A2 deficiency induces VSMCs phenotypic switching at early stages after Ang II infusion.

(**A and B**) *Slc44a2*^{WT} and *Slc44a2*^{SMKO} mice were infused with saline or Ang II (1000 ng/kg/min) for 3 days. (**A**) Immunofluorescence staining for OPN (red), ACTA2 (green), and DAPI (blue) in the suprarenal abdominal aorta. Scale bar, 200 µm. n = 5. (**B**) Immunofluorescence images of in situ zymography (DQ gelatin, green) in the suprarenal abdominal aorta. Scale bar, 200 µm. n = 5. (**C and D**) *Slc44a2*^{WT} and *Slc44a2*^{SMKO} mice were infused with saline or Ang II (1000 ng/kg/min) for 7 days. (**C**) Immunofluorescence staining for OPN (red), ACTA2 (green), and DAPI (blue) in the suprarenal abdominal aorta. Scale bar, 200 µm. n = 5. (**D**) Immunofluorescence images

of in situ zymography (DQ gelatin, green) in the suprarenal abdominal aorta. Scale bar, 200 μm. n = 5. **A-C**, one-way ANOVA; **D**, Welch's ANOVA.

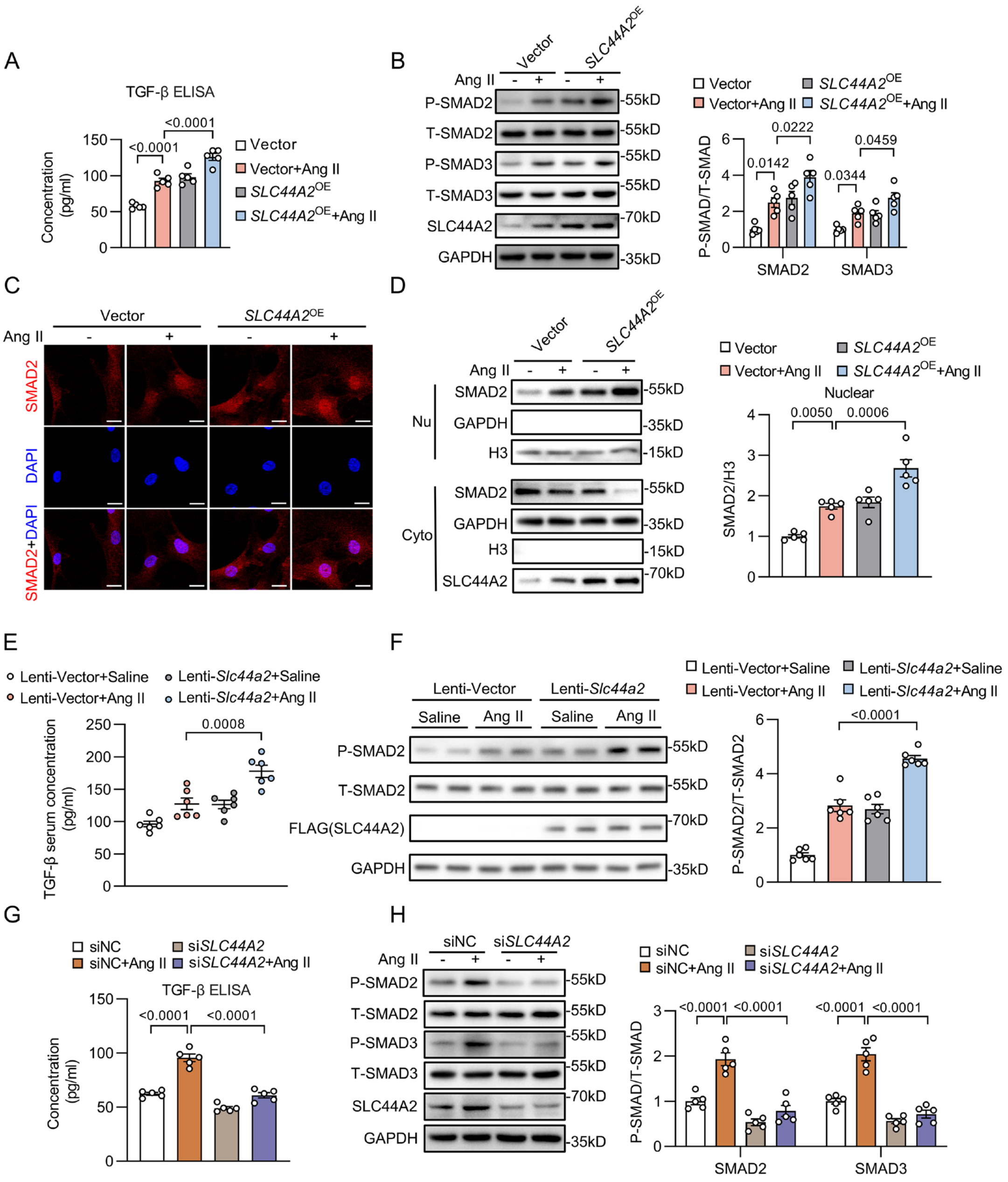

Supplemental Figure 6. SLC44A2 activates TGF-β signaling.

(**A-D**) HASMCs were infected with lentivirus containing vector or SLC44A2 encoding plasmids, and then treated with Ang II (1 μM). (**A**) The TGF-β level in culture medium was measured by ELISA. n = 5. (**B**) The levels of p-SMAD2 and p-SMAD3 were detected by Western blotting. n = 5. (**C**) The nuclear location of SMAD2 was detected by immunofluorescence staining with SMAD2 (red) and DAPI (blue). Scale bar, 20 μm. n = 3. (**D**) The SMAD2 level in cytoplasm and nucleus was detected by Western blotting. n = 5. (**E** and **F**) Eight-week-old male *Apoe*$^{-/-}$/*Tagln*$^{Cre/+}$ mice were intravenously injected with lentivirus containing control vector or Slc44a2. After 2 weeks, osmotic

pumps were implanted to infuse saline or Ang II for 28 days. (**E**) The serum TGF-β level was measured by ELISA. n = 6. (**F**) The levels of p-SMAD2 in the whole aorta were detected by Western blotting. n = 6. (**G** and **H**) HASMCs were transfected with si*SLC44A2* or siNC and then treated with Ang II (1 μM). (**G**) The TGF-β level in culture medium was measured by ELISA. n = 5. (**H**) The levels of p-SMAD2 and p-SMAD3 were detected by Western blotting. n = 5. **A**, **B**, and **D-H**, one-way ANOVA.

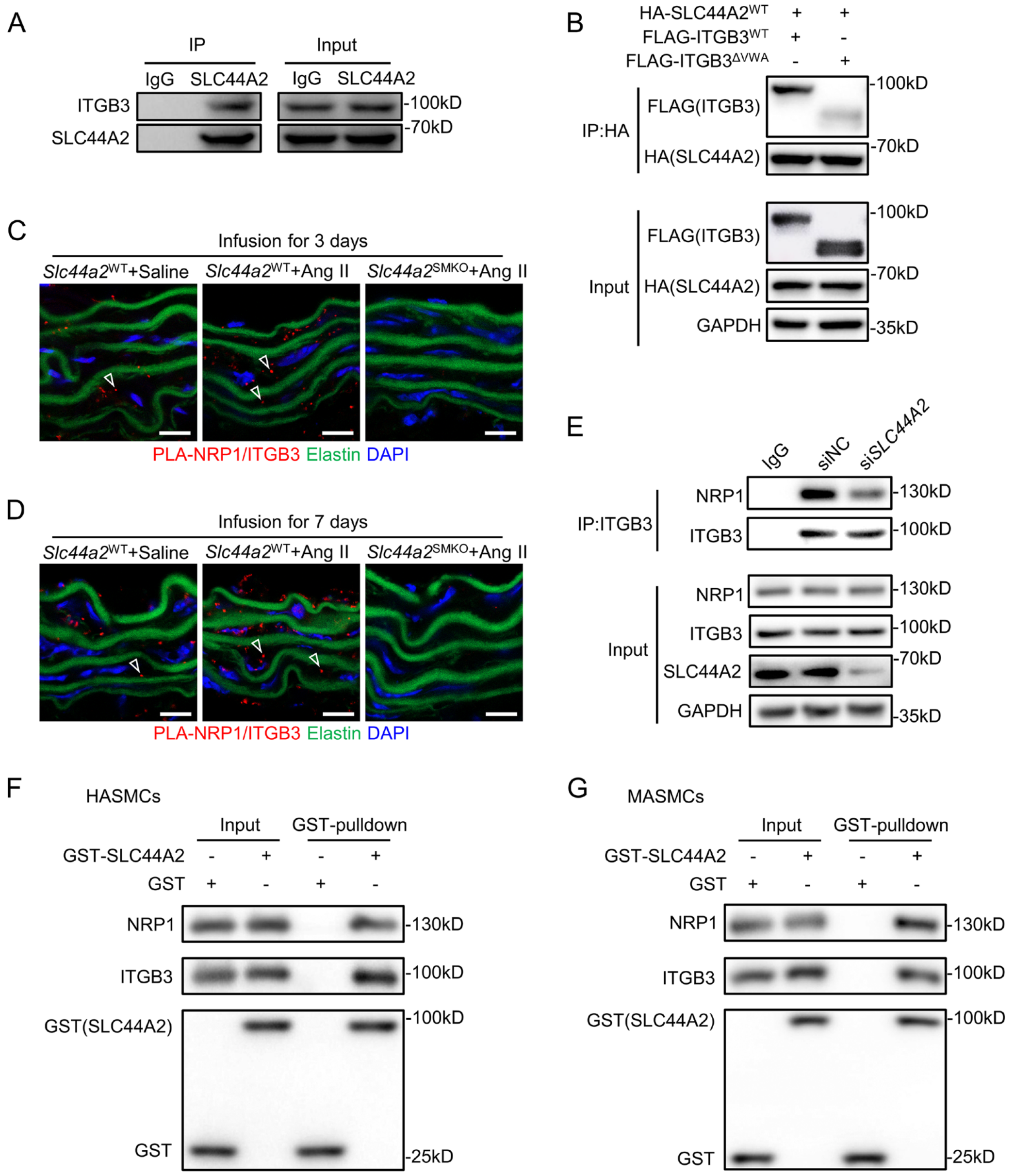

Supplemental Figure 7. SLC44A2 mediates the interaction of NRP1 and ITGB3. (**A**) Lysates of HASMCs were immunoprecipitated with anti-SLC44A2 antibody, and blotted with anti-ITGB3 and anti-SLC44A2 antibodies. n = 3. (**B**) HEK293 cells were transfected with $SLC44A2^{WT}$ plasmid and plasmids encoding $ITGB3^{WT}$ and $ITGB3^{\Delta VWA}$ for 24 hours. Lysates were immunoprecipitated with anti-HA antibody, and blotted with anti-Flag and anti-HA antibodies. n = 4. (**C**) *Slc44a2*^{WT} and *Slc44a2*^{SMKO}

mice were infused with saline or Ang II (1000 ng/kg/min) for 3 days. The interaction of NRP1 with ITGB3 in suprarenal abdominal aorta was detected by PLA. Scale bar, 10 μm. n = 5. (**D**) *Slc44a2*^{WT} and *Slc44a2*^{SMKO} mice were infused with saline or Ang II (1000 ng/kg/min) for 7 days. The interaction of NRP1 with ITGB3 in suprarenal abdominal aorta was detected by PLA. Scale bar, 10 μm. n = 5. (**E**) HASMCs were transfected with si*SLC44A2* or siNC for 24 hours. Lysates were immunoprecipitated with anti-ITGB3 antibody, and blotted with anti-NRP1 and anti-ITGB3 antibodies. n = 3. (**F**) GST pull-down assays were performed with purified GST or GST-SLC44A2 from E.coli and total proteins from HASMCs. n = 3. (**G**) GST pull-down assays were performed with purified GST or GST-SLC44A2 from E.coli and total proteins from MASMCs. n = 3.

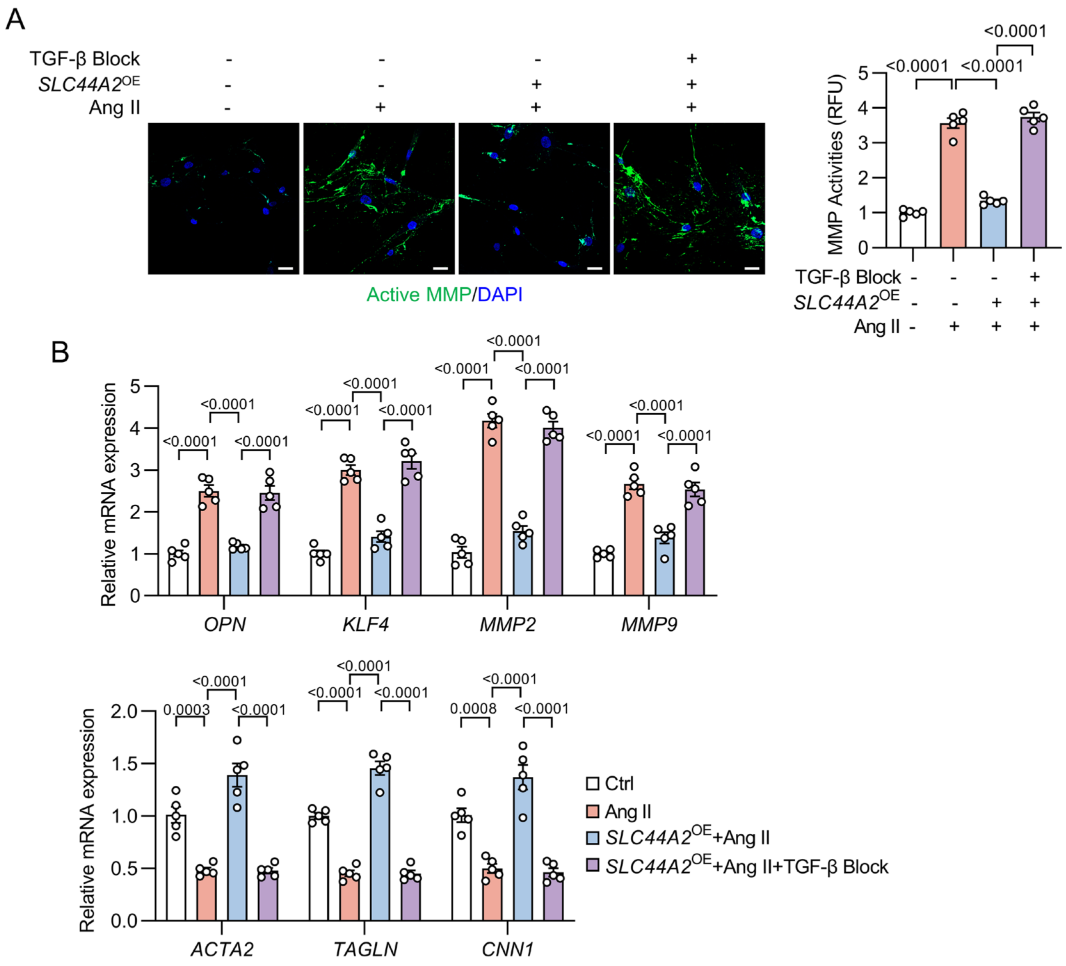

Supplemental Figure 8. The effect of SLC44A2 overexpression is abolished by TGF-β block.

(**A** and **B**) HASMCs were infected with lentivirus containing vector or SLC44A2 encoding plasmids, and then treated by Ang II (1 μM, 24 hours) with or without TGF-β neutralizing antibody (10 μg/mL). (**A**) Immunofluorescence images of in situ zymography (DQ gelatin) in HASMCs. MMP activity was quantified by immunofluorescence intensity. Scale bar, 40 μm. n = 5. (**B**) qRT-PCR analysis of

VSMCs synthetic and contractile markers in HASMCs. n = 5. **A** and **B**, one-way ANOVA.

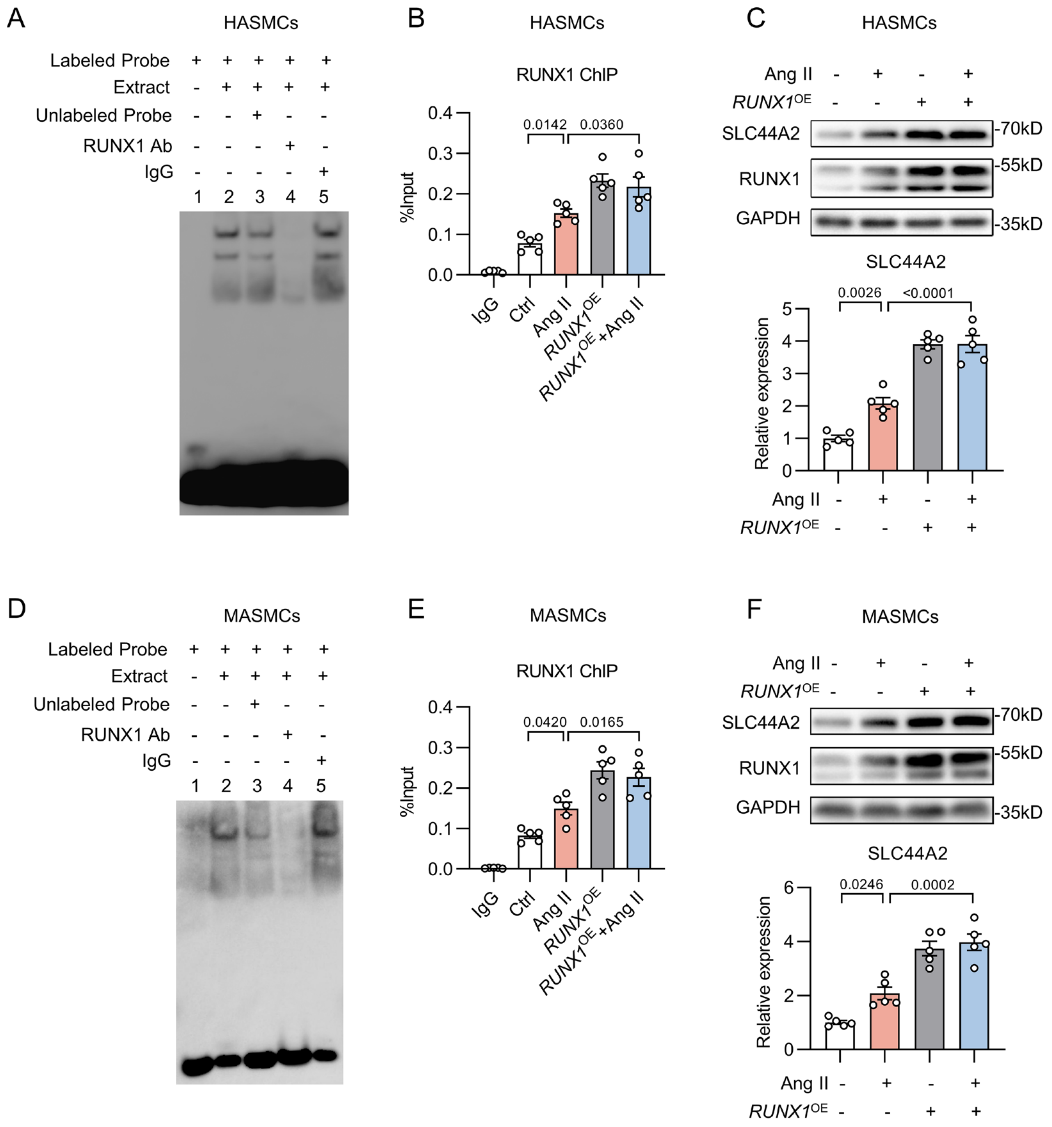

Supplemental Figure 9. RUNX1 regulates SLC44A2 transcription both in HASMCs and MASMCs.

(**A**) EMSA was performed with nuclear extract from HASMCs by oligonucleotide probe with RUNX1 bind sites. Lane 1 indicates no extract; lane 2, protein binding with extract; lane 3, competition with excess unlabeled probe; lane 4, inhibition of binding with RUNX1 antibody; and lane 5, effect of nonspecific IgG. n = 3. (**B** and **C**) HASMCs were infected with Lenti-Vector or Lenti-*RUNX1*, and treated by vehicle or Ang II (1 µM, 24 hours). (**B**) Chromatin immunoprecipitation (ChIP) assays were performed with IgG or anti-RUNX1 antibody, followed by qRT-PCR with primers targeting SLC44A2 promoter regions. n = 5. (**C**) The levels of SLC44A2 and RUNX1 were detected by Western blotting. n = 5. (**D**) EMSA was performed with nuclear extract from MASMCs isolated from the whole abdominal aortas by oligonucleotide probe with RUNX1 bind

sites. Lane 1 indicates no extract; lane 2, protein binding with extract; lane 3, competition with excess unlabeled probe; lane 4, inhibition of binding with RUNX1 antibody; and lane 5, effect of nonspecific IgG. n = 3. (**E** and **F**) MASMCs isolated from the whole abdominal aortas were infected with Lenti-Vector or Lenti-*RUNX1*, and treated by vehicle or Ang II (1 μM, 24 hours). (**E**) Chromatin immunoprecipitation (ChIP) assays were performed with IgG or anti-RUNX1 antibody, followed by qRT-PCR with primers targeting SLC44A2 promoter regions. n = 5. (**F**) The levels of SLC44A2 and RUNX1 were detected by Western blotting. n = 5. **B**, **C**, **E**, and **F**, one-way ANOVA.

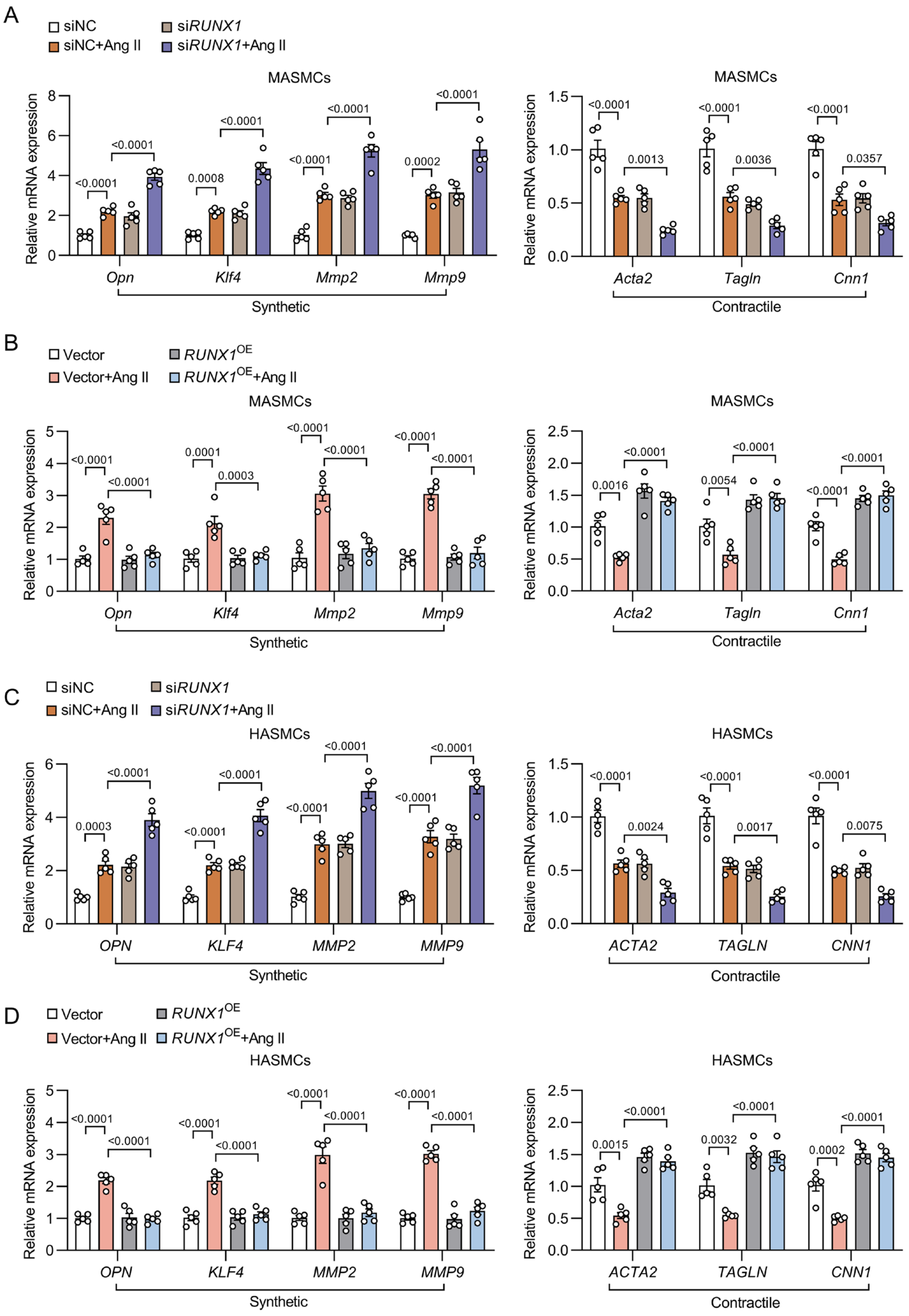

Supplemental Figure 10. RUNX1 regulates VSMCs phenotypic switching.
(**A**) MASMCs isolated from the whole abdominal aortas were transfected with si*RUNX1* or siNC, and then treated with Ang II (1 μM, 24 hours). qRT-PCR analysis of VSMCs synthetic markers (*Opn*, *Klf4*, *Mmp2*, and *Mmp9*) and contractile markers (*Acta2*, *Tagln*, and *Cnn1*). n = 5. (**B**) MASMCs isolated from the whole abdominal aortas were infected with lentivirus containing vector or RUNX1 encoding plasmids to overexpress RUNX1 (*RUNX1*^{OE}), and then treated with Ang II (1 μM, 24 hours). qRT-

PCR analysis of VSMCs synthetic markers (*Opn*, *Klf4*, *Mmp2*, and *Mmp9*) and contractile markers (*Acta2*, *Tagln*, and *Cnn1*). n = 5. (**C**) HASMCs were transfected with si*RUNX1* or siNC, and then treated with Ang II (1 μM, 24 hours). qRT-PCR analysis of VSMCs synthetic markers (*OPN*, *KLF4*, *MMP2*, and *MMP9*) and contractile markers (*ACTA2*, *TAGLN*, and *CNN1*). n = 5. (**D**) HASMCs were infected with lentivirus containing vector or RUNX1 encoding plasmids to overexpress RUNX1 (*RUNX1*^{OE}), and then treated with Ang II (1 μM, 24 hours). qRT-PCR analysis of VSMCs synthetic markers (*OPN*, *KLF4*, *MMP2*, and *MMP9*) and contractile markers (*ACTA2*, *TAGLN*, and *CNN1*). n = 5. **A**-**D**, one-way ANOVA.

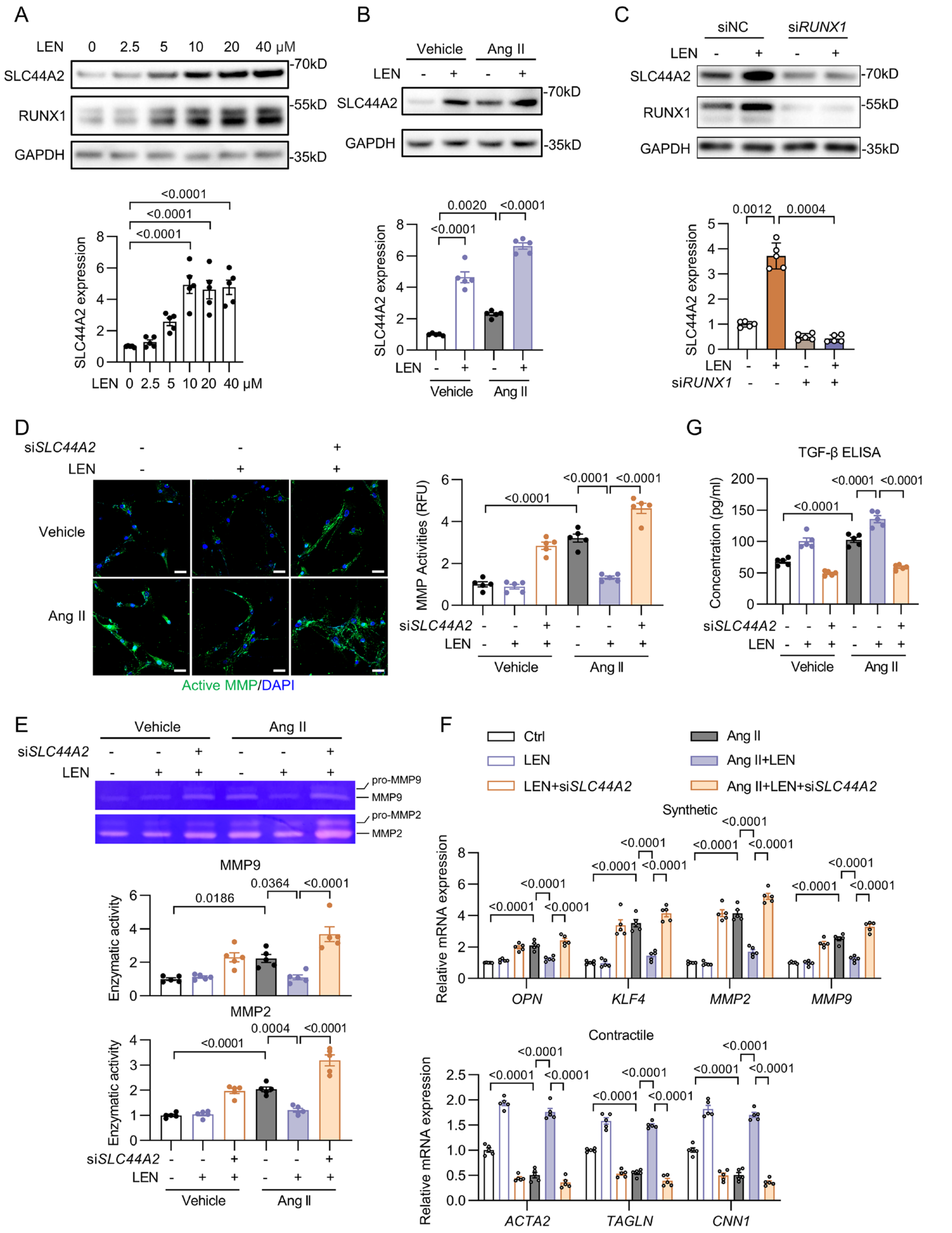

Supplemental Figure 11. LEN may enhance SLC44A2 expression in a RUNX1-dependent manner to maintain VSMCs contractile phenotype.

(**A**) The expression of SLC44A2 and RUNX1 in HASMCs treated by LEN (2.5/5/10/20/40 μM, 24 hours) was detected by Western blotting. n = 5. (**B**) The expression of SLC44A2 in HASMCs treated by Ang II (1 μM, 24 hours) with or without LEN (10 μM, 24 hours). n = 5. (**C**) HASMCs were transfected with si*RUNX1* and then treated by LEN (10 μM) for 24 hours. The expression of SLC44A2 was detected by Western blotting. n = 5. (**D-G**) HASMCs were transfected with si*SLC44A2* and then treated by Ang II (1 μM) and LEN (10 μM) for 24 hours. (**D**) Immunofluorescence

images of in situ zymography (DQ gelatin) in HASMCs. MMP activity was quantified by immunofluorescence intensity. Scale bar, 40 μm. n = 5. (**E**) The activity of MMP2 and MMP9 in culture medium was measured by gel zymography. n = 5. (**F**) The levels of VSMCs synthetic and contractile markers were detected by qRT-PCR. n = 5. (**G**) The TGF-β level in culture medium was measured by ELISA. n = 5. **A**, **B**, **D**-**G**, one-way ANOVA; **C**, Welch's ANOVA.

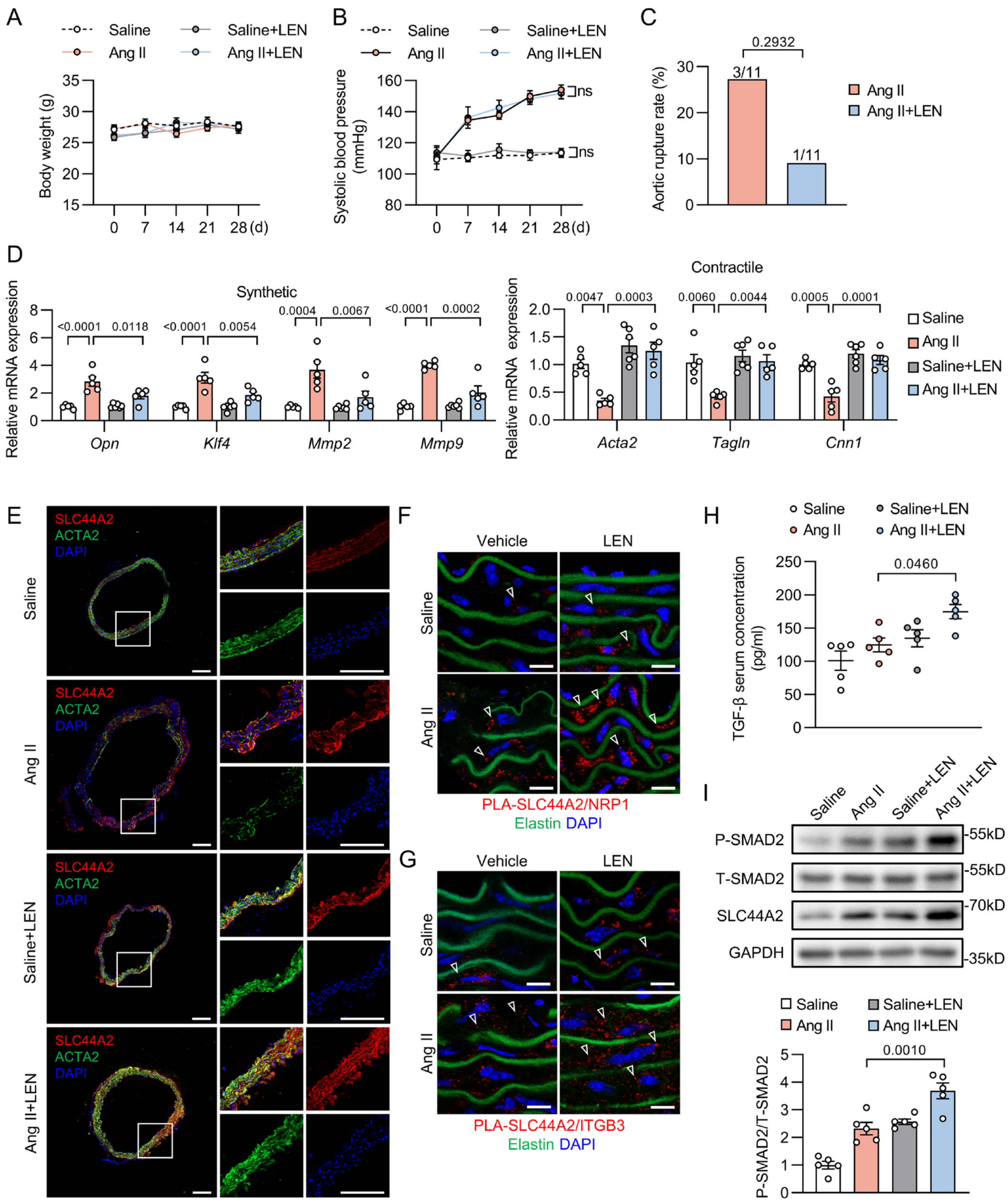

Supplemental Figure 12. LEN mitigates VSMCs dedifferentiation in Ang II-infused mice via activating TGF-β/SMAD pathway by promoting SLC44A2/NRP1/ITGB3 complex.

(**A-I**) Eight- to ten-week-old male *Apoe*$^{-/-}$ mice were implanted subcutaneously with osmotic pumps to infuse saline or Ang II (1000 ng/kg/min) with or without intragastric administration of LEN (20 mg/kg/day) for 28 days. (**A**) The body weight of Ang II-infused *Apoe*$^{-/-}$ mice with vehicle or LEN administration at 0, 7, 14, 21, and 28 days. n = 8-11. (**B**) The systolic blood pressure of Ang II-infused *Apoe*$^{-/-}$ mice with vehicle or LEN administration at 0, 7, 14, 21, and 28 days. n = 8-11. ns, no significance. (**C**) The aortic rupture rate in Ang II-infused *Apoe*$^{-/-}$ mice administrated with vehicle or LEN. n = 11. (**D**) qRT-PCR analysis of VSMCs synthetic markers (*Opn*, *Klf4*, *Mmp2*, and *Mmp9*) and contractile markers (*Acta2*, *Tagln*, and *Cnn1*) in the whole aorta from saline- or Ang II-infused mice. n = 5-6. (**E**) Immunofluorescence staining for SLC44A2

(red), ACTA2 (green), and DAPI (blue) in the suprarenal abdominal aorta. Scale bar, 200 μm. n = 3. (**F**) The interaction of SLC44A2 with NRP1 (red dots marked by arrowheads) in the suprarenal abdominal aorta was detected by PLA. Scale bar, 10 μm. n = 3. (**G**) The interaction of SLC44A2 with ITGB3 (red dots marked by arrowheads) in the suprarenal abdominal aorta was detected by PLA. Scale bar, 10 μm. n = 3. (**H**) The serum TGF-β level was measured by ELISA. n = 5. (**I**) The level of p-SMAD2 in the whole aorta was detected by Western blotting. n = 5. **A** and **B**, two-way ANOVA with mixed-effects; **C**, Fisher's exact test; **D**, **H**, and **I**, one-way ANOVA.

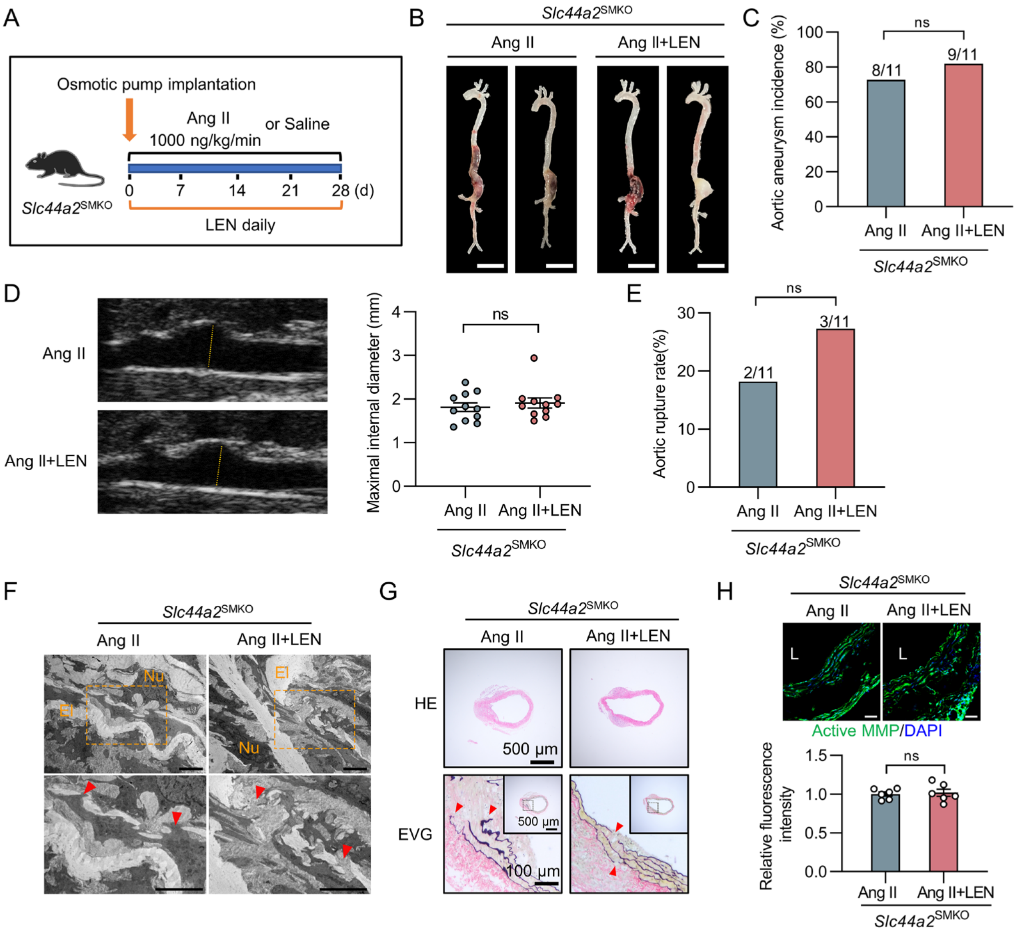

Supplemental Figure 13. SLC44A2 deficiency abolishes the protective effect of LEN in vivo.

(**A**) Eight- to ten-week-old male *Slc44a2*^{SMKO} mice were infused with saline or Ang II (1000 ng/kg/min) for 28 days by osmotic pumps with or without intragastric administration of LEN (20 mg/kg/day). (**B**) Representative morphology of aortas from Ang II-infused *Slc44a2*^{SMKO} mice administrated with vehicle or LEN. Scale bar, 5 mm. n = 11. (**C**) The incidence of aortic aneurysm in Ang II-infused *Slc44a2*^{SMKO} administrated with vehicle or LEN. n = 11. (**D**) Ultrasound images and inner diameter quantification of the suprarenal abdominal aorta in Ang II-infused *Slc44a2*^{SMKO} mice administrated with vehicle or LEN. n = 11. (**E**) The aortic rupture rate in Ang II-infused *Slc44a2*^{SMKO} mice administrated with vehicle or LEN. n = 11. (**F**) Electron microscopic

images of the suprarenal abdominal aorta in Ang II-infused *Slc44a2*^{SMKO} mice administrated with vehicle or LEN. Red arrowheads indicate elastin breaks. El, elastin. Nu, Nucleus. Scale bar, 5 µm. n = 5. (**G**) Hematoxylin and eosin (HE) and elastic Verhoeff-Van Gieson (EVG) staining of the suprarenal abdominal aorta from Ang II-infused *Slc44a2*^{SMKO} mice administrated with vehicle or LEN. Red arrowheads indicate elastin breaks. n = 5. (**H**) Immunofluorescence images of in situ zymography (DQ gelatin, green) in the suprarenal abdominal aorta from Ang II-infused *Slc44a2*^{SMKO} mice administrated with vehicle or LEN. L indicates lumen. Scale bar, 40 µm. n = 6. **C** and **E**, Fisher's exact test; **D**, Mann-Whitney *U* test; **H**, unpaired two-tailed *t*-test.

Supplemental Methods

Cytokines and reagents

Angiotensin II (Ang II, #M6240) was purchased from Abmole Bioscience Inc. TGF-β neutralizing antibody (#MAB1835) was obtained from R&D System. The following antibodies were used in Western blotting and immunofluorescence staining: SLC44A2 (#ARP44009_P050) antibody, Aviva Systems Biology; SLC44A2 (#sc-101266), NRP1 (#sc-5307), KLF4 (#sc-393462), OPN (#sc-21742), and RUNX1 (#sc-101146) antibodies, Santa Cruz Biotechnology; ACTA2 (#ab124964), TAGLN (#ab14106), NRP1 (#ab81321), GAPDH (#ab8245), Histone H3 (#ab1791), and Alexa Fluor® 647-conjugated fluorescent secondary antibodies (#ab150063), Abcam; FLAG tag (#66008-3-Ig), HA tag (#51064-2-AP), His tag (#66005-1-Ig), OPN (#22952-1-AP), and ITGB3 (#18309) antibodies, Proteintech; ACTA2 (#48938), Tubulin (#5335), P-SMAD2 (#18338), T-SMAD2 (#5339), P-SMAD3 (#9520), and T-SMAD3 (#9523) antibodies, Cell Signaling Technology; Alexa Fluor® 594- or 488-conjugated fluorescent secondary antibodies (#A21203, #A21206), Thermo Fisher Scientific. Nuclei were stained with 4, 6-diamidino-2-phenylindole (DAPI, #0100-20) from SouthernBiotech. Lenalidomide (LEN, #S1029) was purchased from Selleck.

Immunofluorescent staining on human paraffin-embedded aorta

Aorta sections were deparaffinized and rehydrated by successive washes of xylene, graded alcohol (vol/vol, 100%, 95%, 90%, 80%, and 75%), and double distilled H_2O (ddH_2O), followed by antigen retrieval. Sections were then permeabilized with 0.3%

Triton X-100 for 10 min and blocked in 10% BSA for 1 h. The tissue sections were then incubated overnight with primary antibodies against SLC44A2 and ACTA2. The negative controls were sections incubated with species-matched IgG. Following three washes in PBS, the samples were incubated for 1 h at room temperature with corresponding fluorescence secondary antibodies conjugated to Alexa Fluor 488 or 594. DAPI was used for nuclear staining. Slides were visualized under a confocal microscope (Zeiss LSM 800).

Blood pressure measurements

Blood pressure (BP) was measured weekly by an automated tail-cuff BP-2000 Blood Pressure Analysis System (Visitech Systems) for 4 weeks. For BP measurements, mice were placed in tail-cuff restrainers situated on a warmed surface. To ensure accurate measurements, mice were habituated to the device prior to the measurement of blood pressures. One set of 15 measurements were obtained for each mouse, and mean blood pressure was calculated.

Blood sampling

Whole blood samples of mice for hematological analyses were collected by tubes containing EDTA as an anticoagulant. Then blood counts were obtained from ADVIA (Siemens Heathcare) using mouse settings. Samples for biochemical assessment were placed into tubes without additive and centrifuged at 3,000 g for 10 min to separate the serum. Serum biochemical parameters were measured by an automatic biochemical analyzer.

Histopathologic analysis

Suprarenal abdominal aortas were excised and fixed in 4% PFA overnight before embedded in paraffin. Serial 5-μm-thick transverse sections were stained with hematoxylin and eosin (HE) to evaluate the aortic structure and morphology or Verhoeff-Van Gieson (EVG) to clarify fragmentation of elastic fibers. HE (#C0105S, Beyotime Biotechnology) and EVG staining kits (#ab150667, Abcam) were used according to the provided protocols. All images were captured by a light microscope (U-HGLGPS, Olympus).

Immunofluorescence staining on cryosections

Suprarenal abdominal aortas of mice were embedded in Optimal Cutting Temperature Compound (OCT, #4583, sakura) and sliced into 8-μm-thick sections. Frozen sections were fixed with 4% PFA for 20 min, permeabilized with 0.3% Triton X-100 for 15 min, and blocked in 10% BSA for 1 h. Sections were incubated with primary antibodies at 4°C overnight. The negative controls were sections incubated with species-matched IgG. After washing, Alexa Fluor® 594- or 488-conjugated fluorescent secondary antibodies were added. Nuclei were labeled with DAPI for 10 min, and imaging was conducted on a confocal microscope (Zeiss LSM 800).

TGF-β ELISA

Levels of endogenously active TGF-β in mouse serum and supernatant of VSMCs were measured using TGF-β ELISA Kit (#PT878 and #PT880, Beyotime Biotechnology) according to the manufacturer's instructions. Samples were diluted and incubated with

specific capture antibody and detection antibody. Absorbance was read using a microplate reader set to 450 nm. Concentrations were calculated using a standard curve generated with specific standards provided by the manufacturer.

Cellular immunofluorescence staining

Treated cells were fixed with 4% PFA for 20 min and permeabilized with 0.3% Triton X-100. Nonspecific binding sites were blocked with 3% BSA for 1 h. Cells were then stained with SMAD2 antibody at 4°C overnight. Afterwards, cells were incubated with Alexa Fluor® 594-conjugated fluorescent secondary antibody and counterstained by DAPI. Images were acquired by confocal microscope (Zeiss LSM 800).

RNA isolation and quantitative real-time PCR

Total RNA was extracted using TRIzol reagent (#TKR-9109, Takara). RNA was reverse transcribed using HiScript II Q RT SuperMix (Vazyme). Quantitative real-time PCR was performed using SYBR Green Master Mix (#Q131-02, Vazyme) on a QuantStudio qPCR System. mRNA levels were determined from the mean threshold cycle (Ct) values and normalized to the Ct of the internal control, GAPDH, unless stated otherwise. Fold changes were calculated by the $2^{-\Delta\Delta Ct}$ method and results were shown as mean ± SEM relative to the control. Real-time PCR primer pairs are listed in the **Supplemental Table 1**.

Protein extraction and Western blot analysis

Aortic tissues or cells were lysed using RIPA lysis buffer (#P0013B, Beyotime Biotechnology) containing protease inhibitor cocktail (#78438, Thermo Fisher

Scientific). Protein concentrations were quantified by the BCA method (#23227, Thermo Fisher Scientific). Equal amounts of protein from each sample were separated in SDS-PAGE gels and blotted into PVDF membranes. After blocked for 1.5 h in 5% non-fat milk, the membranes were incubated overnight at 4°C with primary antibodies, followed by the incubation of horseradish peroxidase-conjugated secondary antibodies for 2 h at room temperature. The membranes were scanned and detected by Amersham 600 series Imager (General Electric). Signal intensities were quantified using Image J software.

Co-immunoprecipitation

Total proteins from HASMCs or HEK293 cells were extracted by the lysis buffer (40 mM Hepes, pH 7.4, 2 mM EDTA, 10 mM pyrophosphate, 10 mM glycerophosphate, 0.5% Triton) supplemented with protease inhibitor cocktail. The supernatants were then collected after centrifugation at 12,000 g for 10 min. Cell lysates were mixed with IgG, SLC44A2, NRP1, ITGB3, HA-tag, His-tag, or FLAG-tag antibody at 4°C overnight, followed by precipitation with protein G-agarose beads for 4 h at 4°C. After washing, the immunoprecipitated complexes were immediately identified by SDS-PAGE and Western blot analysis.

Co-immunoprecipitation coupled to mass spectrometry (Co-IP/MS) analysis

SLC44A2 was immunoprecipitated using anti-SLC44A2 antibody to identify proteins interacting with SLC44A2 in HASMCs. The gel was cut into small pieces and in-gel

digestion was performed. Extracted peptides were solubilised in 0.1% TFA, desalted by C18 StageTip (Thermo Fisher Scientific) and then lyophilized.

LC-MS/MS analyses were performed on an Ekspert^{TM} nano LC 415 equipped with a TripleTOF® 5600+ (AB Sciex) mass spectrometer. Obtained Peptides were loaded on a Chrom XP C18 trap column (3 μm, 120 Å, 350 μm 0.5 mm; Eksigent) at a flow rate of 3 μL/min for 10 min and eluted through a separation column (3 μm, 120 Å, 75 μm 150 mm; Eksigent) at a flow rate of 300 nL/min using 98% water/0.1% formic acid and 98% acetonitrile/0.1% formic acid as the mobile phases A and B, respectively. The component of mobile phase B was collected on the basis of the following scheme: 0-0.1 min, 5-9%; 0.1-35 min, 9-25%; 35-45 min, 25-50%; 45-45.1 min, 50-80%; 45.1-50 min, 80%; 50-50.5 min, 80-5%; 50.5-60 min, 5%.

Raw files were processed using the MaxQuant software (version: 1.5.2.8), and searches were conducted against the UniProt reference sequences for human proteome. False discover rates (FDRs) were estimated using the target-decoy method. An FDR cutoff of 0.05 was selected for filtering both peptides and proteins.

Lentivirus construction and cell infection

Lentiviral vectors expressing SLC44A2^{WT} and SLC44A2 mutant (Δ55-232, Δ254-480, Δ505-659) were generated by Viraltherapy Technologies. Recombinant lentiviruses were produced by co-transfecting HEK293T cells with the lentiviral expression and packaging plasmids (plp1, plp2, pVSVG). The viral supernatant was harvested 48 h post-transfection and purified with a 0.45-μm filter before infection. VSMCs were infected with the lentivirus at MOI of 20, supplemented with polybrene (10 μg/mL).

siRNA interference assay

HASMCs were transfected with 50 nM siRNA targeting SLC44A2, ITGB3, or RUNX1 using Lipofectamine 3000 (#1742691, Life), following the manufacturer's protocol. Scrambled siRNA was used as the negative control (NC). 4 h post-transfection, the culture medium was changed to complete growth medium.

Dual luciferase reporter assay

Wild-type and mutant SLC44A2 promoters were cloned into pGL6 luciferase reporter vectors, respectively. The recombinant pGL6 dual luciferase reporter vectors were co-transfected into HEK293T cells with or without RUNX1 encoding plasmid. Subsequently, the fluorescein-labeled reporter gene activity was detected using a Dual-Luciferase Reporter Assay System kit (#E1910, Promega), according to the manufacturer's instructions.

Electrophoretic mobility shift assays (EMSA)

Nuclear extracts from HASMCs or MASMCs were prepared using NE-PER™ Nuclear and Cytoplasmic Extraction Reagents (#78833, Thermo Fisher Scientific) as manufacturer's instructions. Oligonucleotides encompassing the *SLC44A2* promoter region were synthesized from human or mouse genomic DNA by PCR and labeled with Biotin (GenScript Biotech Corporation) (**Supplemental Table 4**). EMSA was performed using Chemiluminescent EMSA Kit (#GS009, Beyotime Biotechnology). Nuclear extracts were incubated with 10 nM biotin-labeled oligos in the binding buffer for 20 min at room temperature. For competitive EMSA, unlabeled oligos were added

into the reaction mixture prior to the addition of labeled oligos. Supershift assays were performed with anti-RUNX1 or IgG antibodies. The DNA-protein complexes were resolved on BeyoGel™ EMSA PAGE (#GS3305S, Beyotime Biotechnology) and visualized by Amersham 600 series Imager (General Electric).

Chromatin immunoprecipitation (ChIP)

ChIP assays were performed on HASMCs or MASMCs using CHIP Assay Kit (#P2078, Beyotime Biotechnology). Briefly, cells were fixed with 10% formaldehyde for 15 min at 37°C and the reaction was quenched by glycine. Cells were then lysed and sonicated to fragment DNA to 100-200 bp. After centrifugation (12,000 × g, 10 min at 4 °C), the chromatin was incubated overnight with anti-RUNX1 or IgG antibodies at 4 °C. Then, the mixture was incubated with protein A/G agarose beads and rotated at 4 °C for 1 h. After washing the beads, the DNA fragments were eluted and subjected to PCR analysis using the primers listed in the **Supplemental Table 5**.

Glutathione S-transferase (GST) pull-down assay

GST and GST-tagged SLC44A2 E.coli expression systems were obtain from Genechem Corporation. Cell lysates were extracted from HASMCs and MASMCs. The GST pull-down assay was performed using Pierce™ GST Protein Interaction Pull-Down Kit (#21516, Thermo Fisher Scientific), following the manufacturer's instructions.
