# Supplementary material for: SLC44A2 regulates vascular smooth muscle cell phenotypic switching and aortic aneurysm
Source: J Clin Invest. 2024 Jun 25;134(16):e173690. doi: 10.1172/JCI173690 (PMC11324303; doi:10.1172/JCI173690)

SLC44A2 regulates vascular smooth muscle cell phenotypic switching and aortic aneurysm

Full unedited gel for Figure 1G **N=6**

Figure 1G

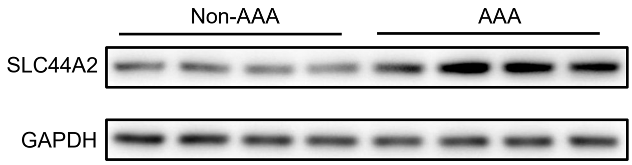

No. 1-4

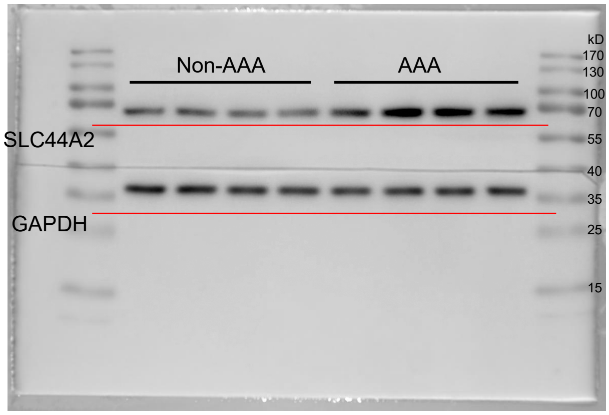

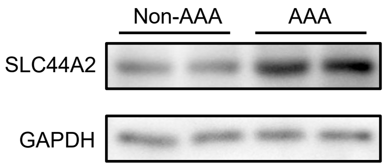

No. 5-6

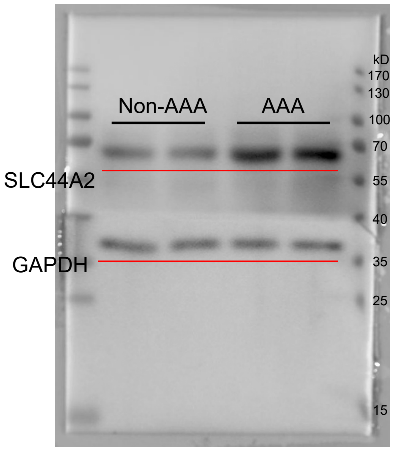

Full unedited gel for Figure 2A **N=5**

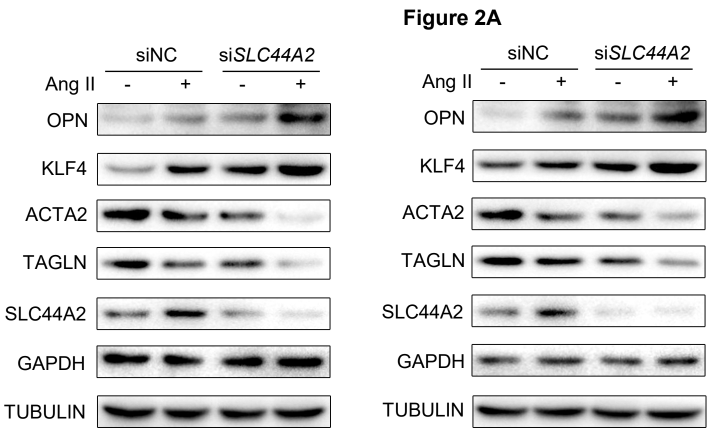

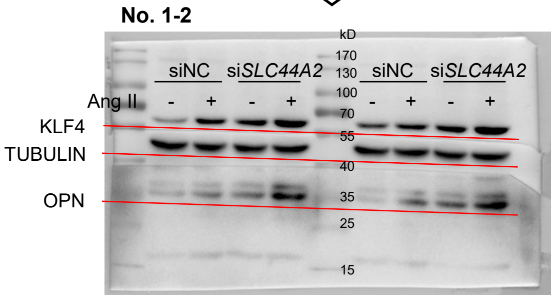

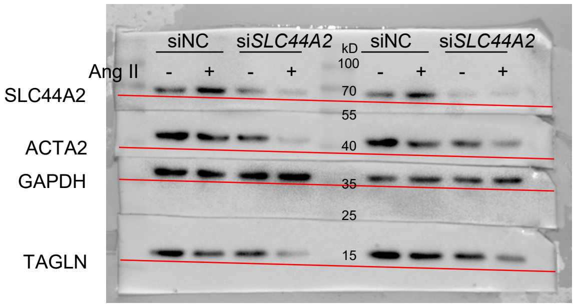

Full unedited gel for Figure 2A **N=5**

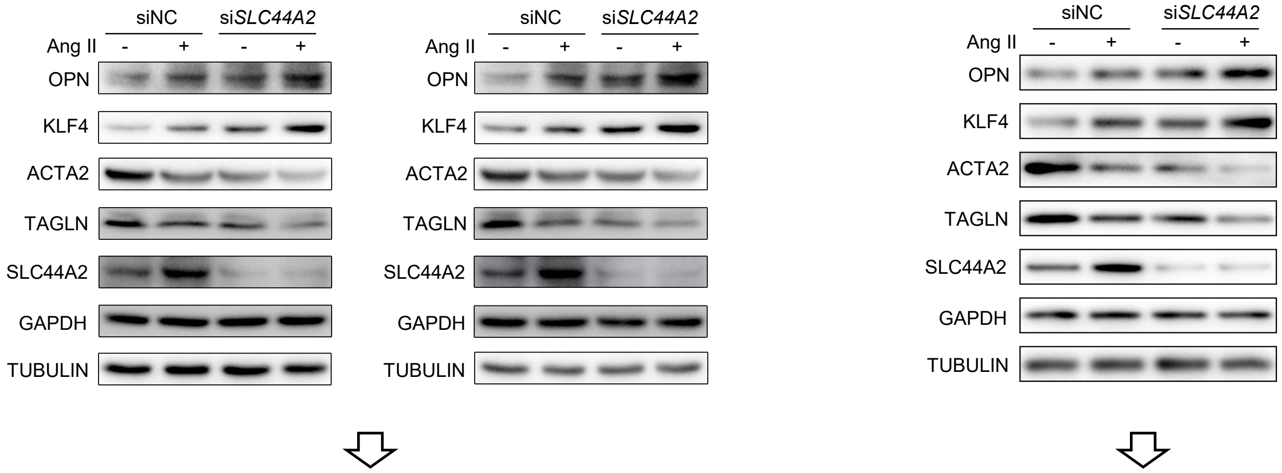

No. 3-4

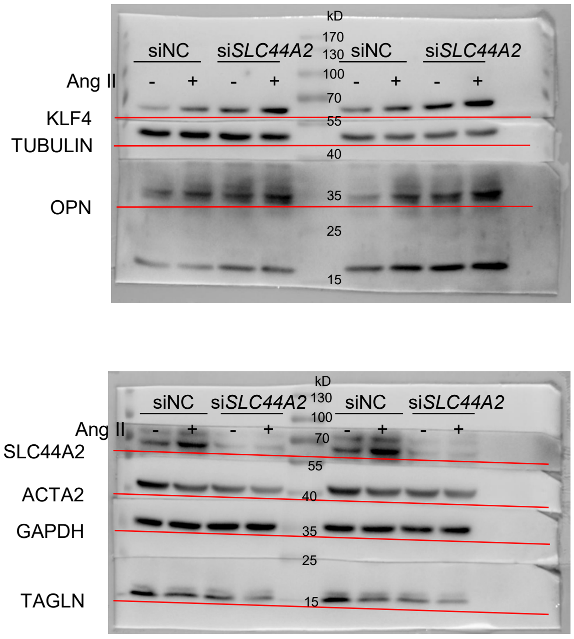

No. 5

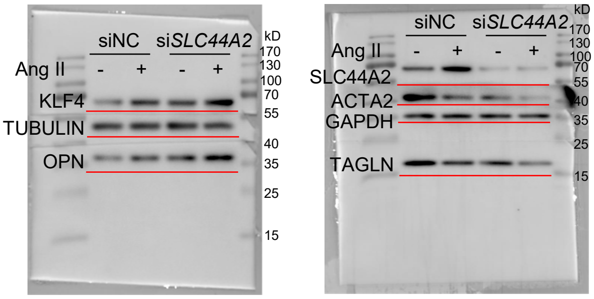

Full unedited gel for Figure 2E N=5

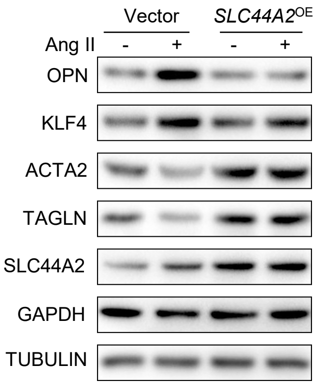

Figure 2E

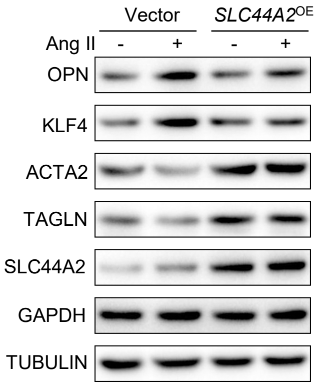

No. 1-2

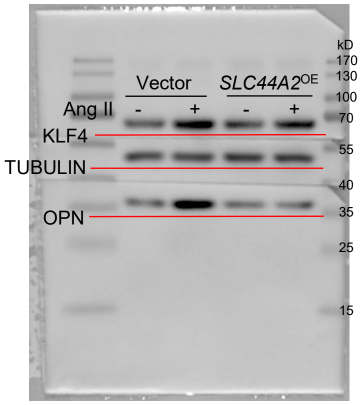

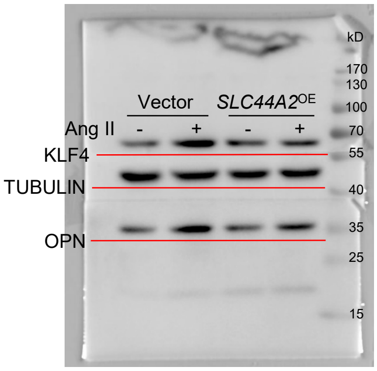

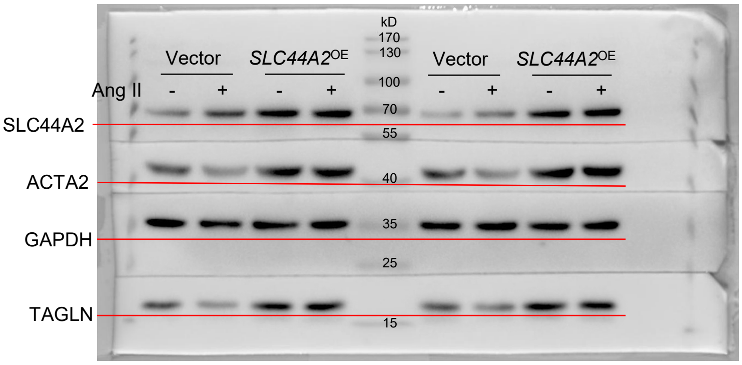

Full unedited gel for Figure 2E   N=5

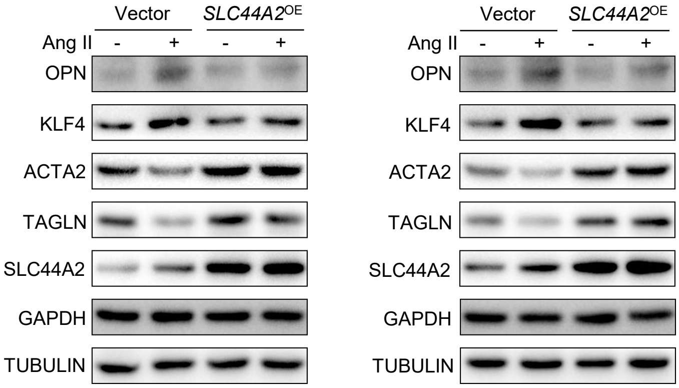

No. 3-4

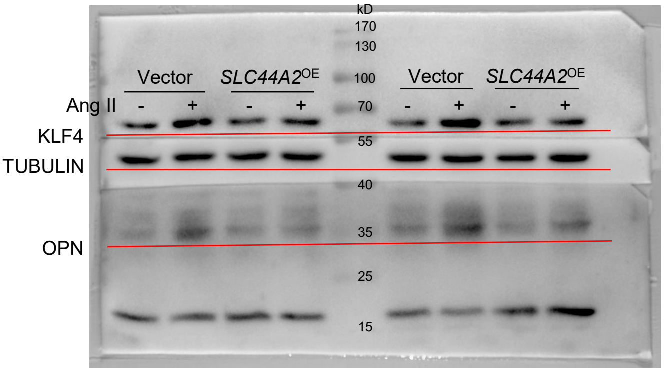

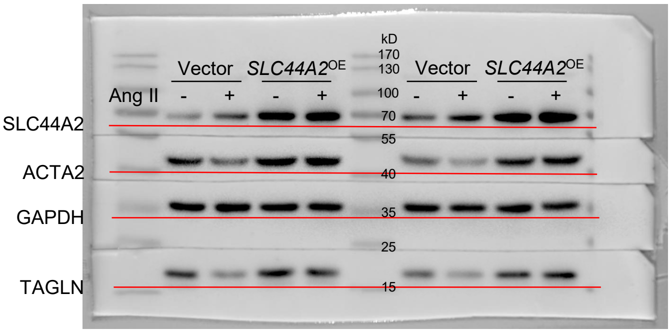

Full unedited gel for Figure 2E   N=5

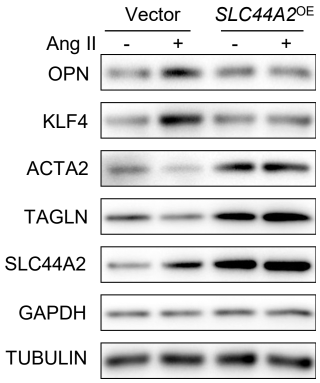

No. 5

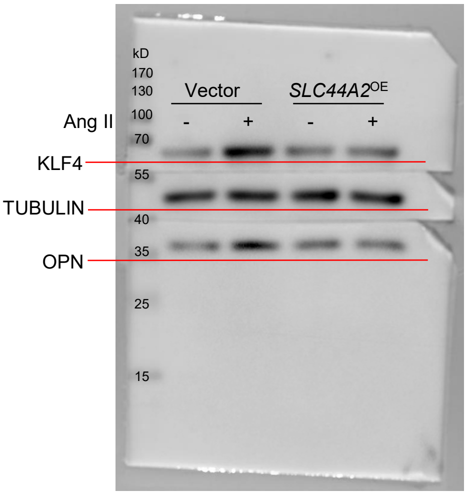

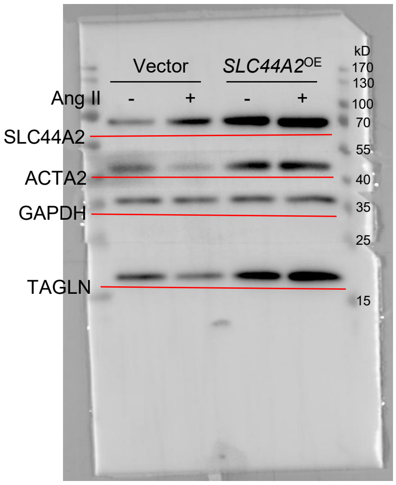

Full unedited gel for Figure 5B N=5

Figure 5B

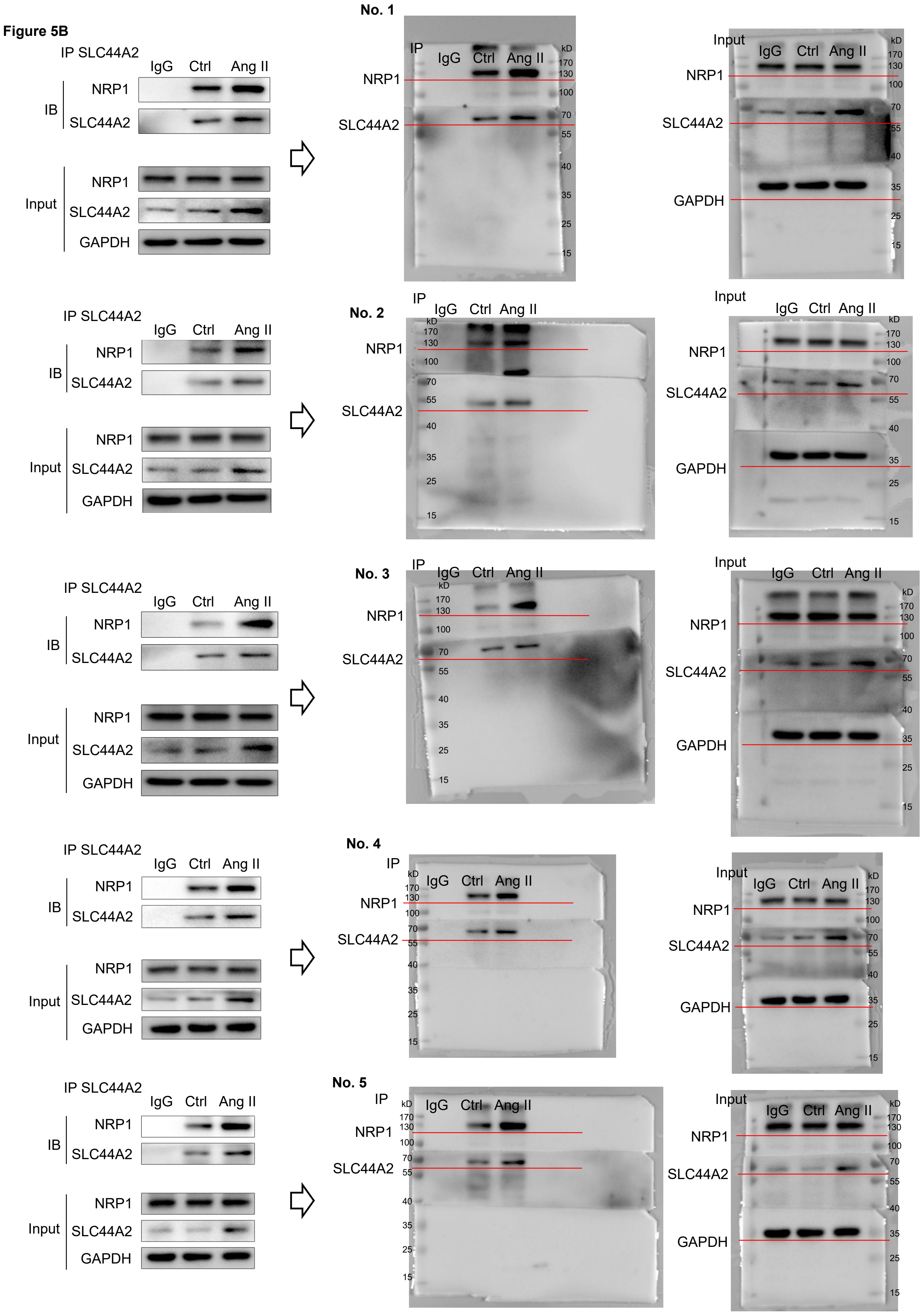

Full unedited gel for Figure 5C N=4

Figure 5C

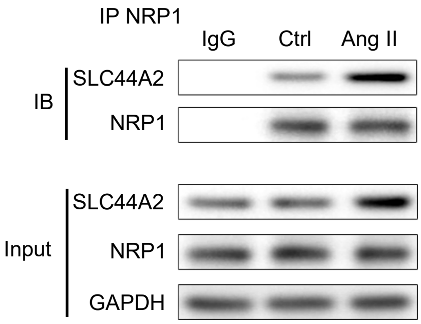

No. 1

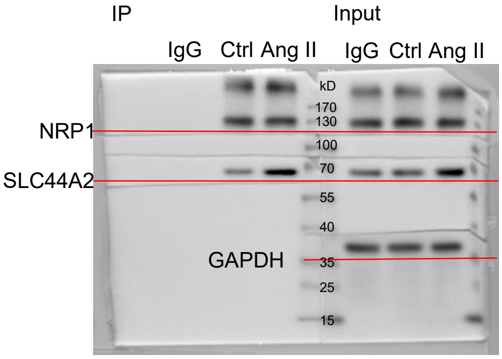

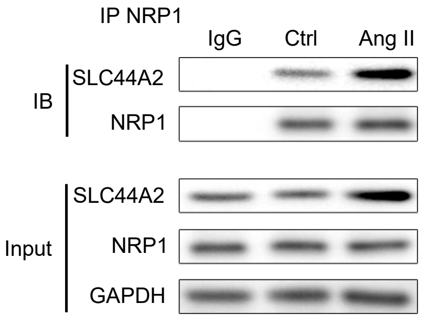

No. 2

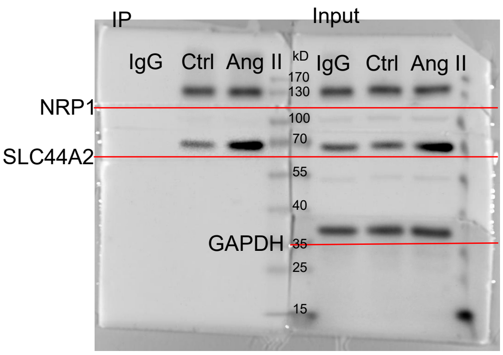

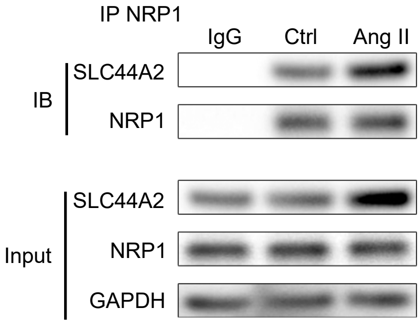

No. 3

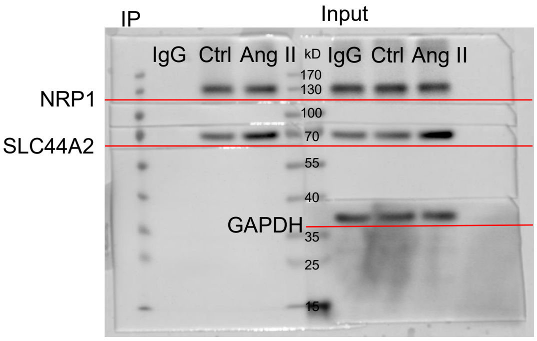

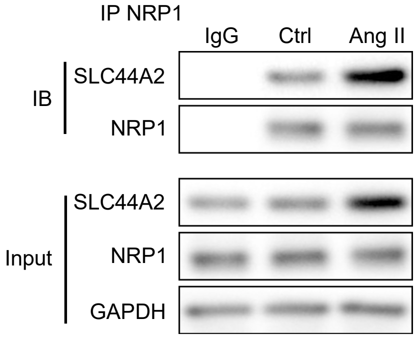

No. 4

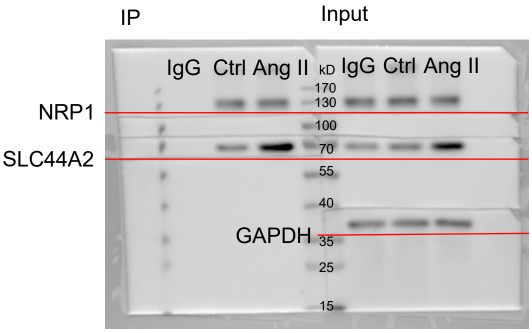

Full unedited gel for Figure 5F N=4

Figure 5F

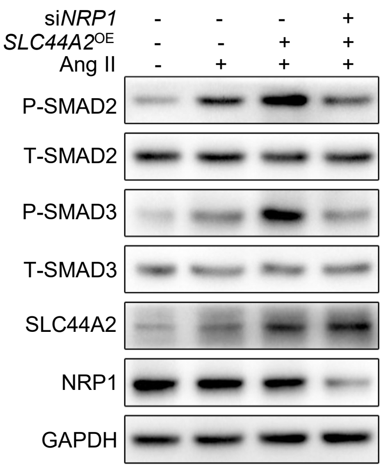

No. 1

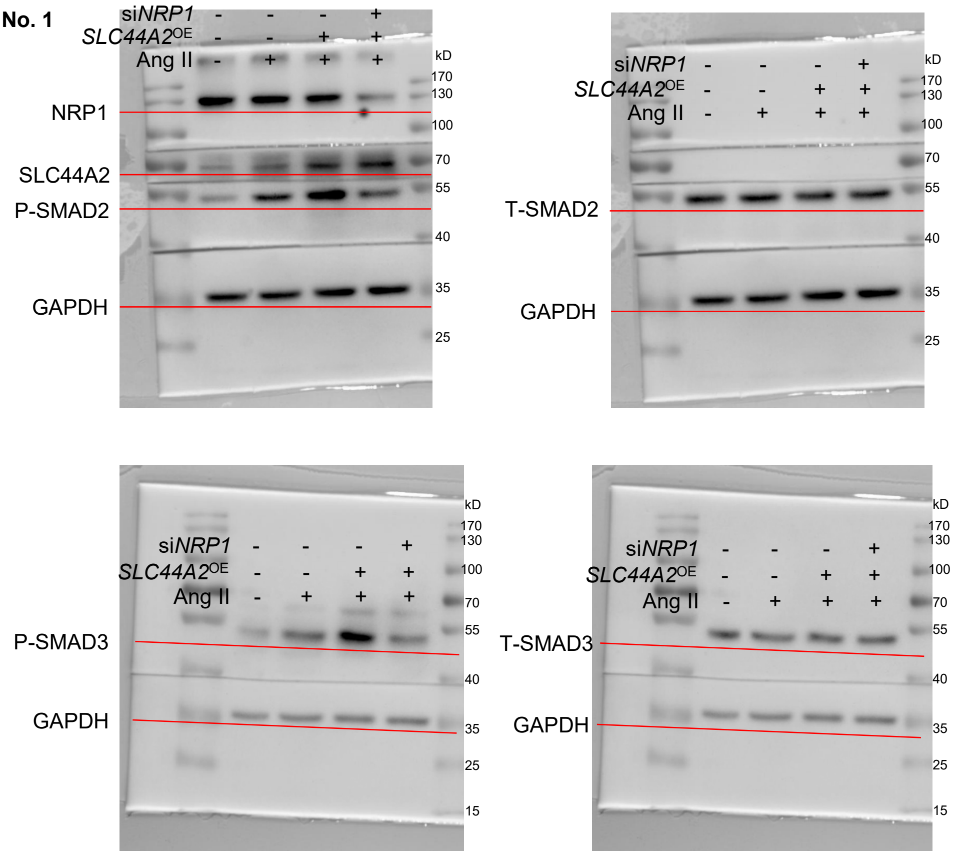

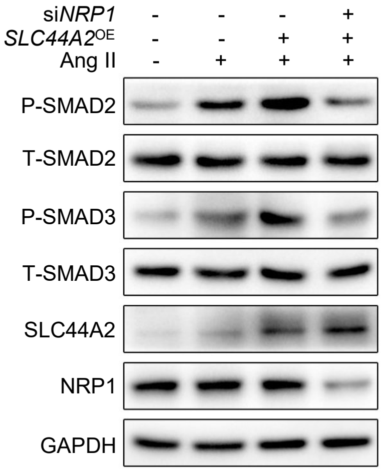

No. 2

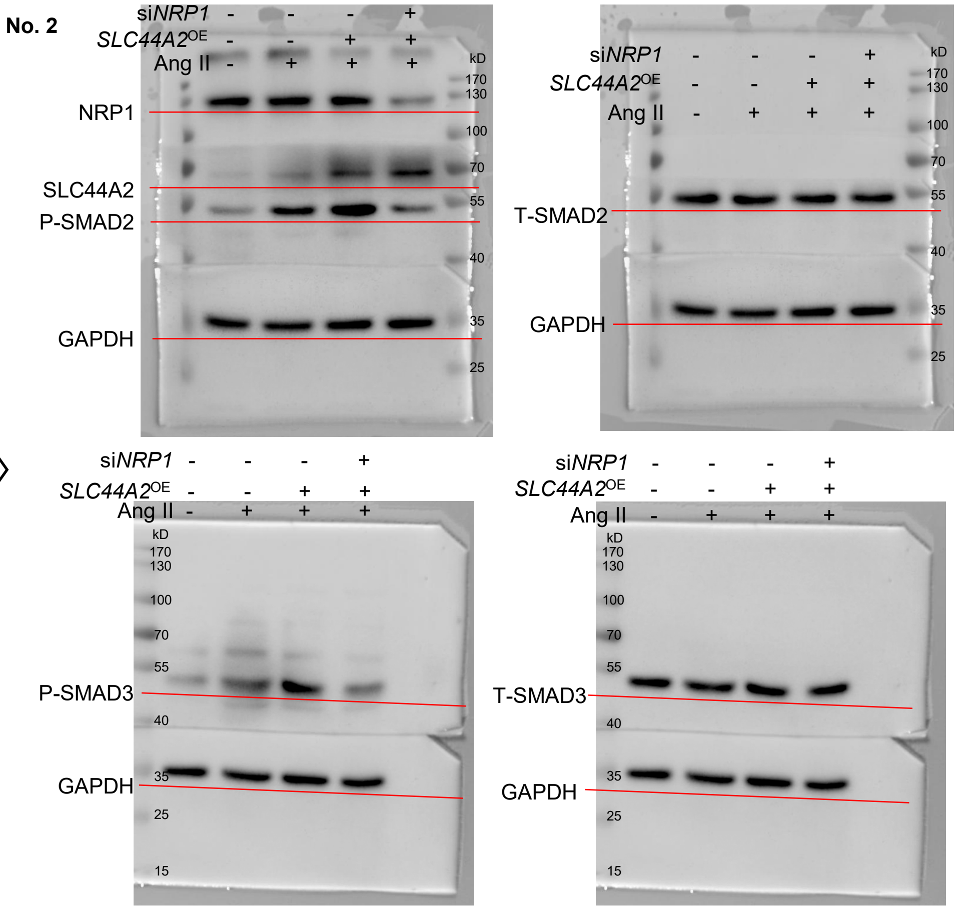

Full unedited gel for Figure 5F   N=4

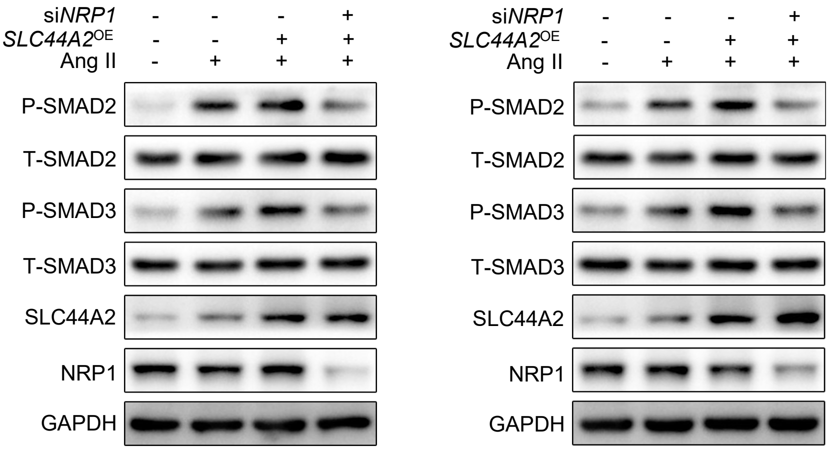

No. 3-4

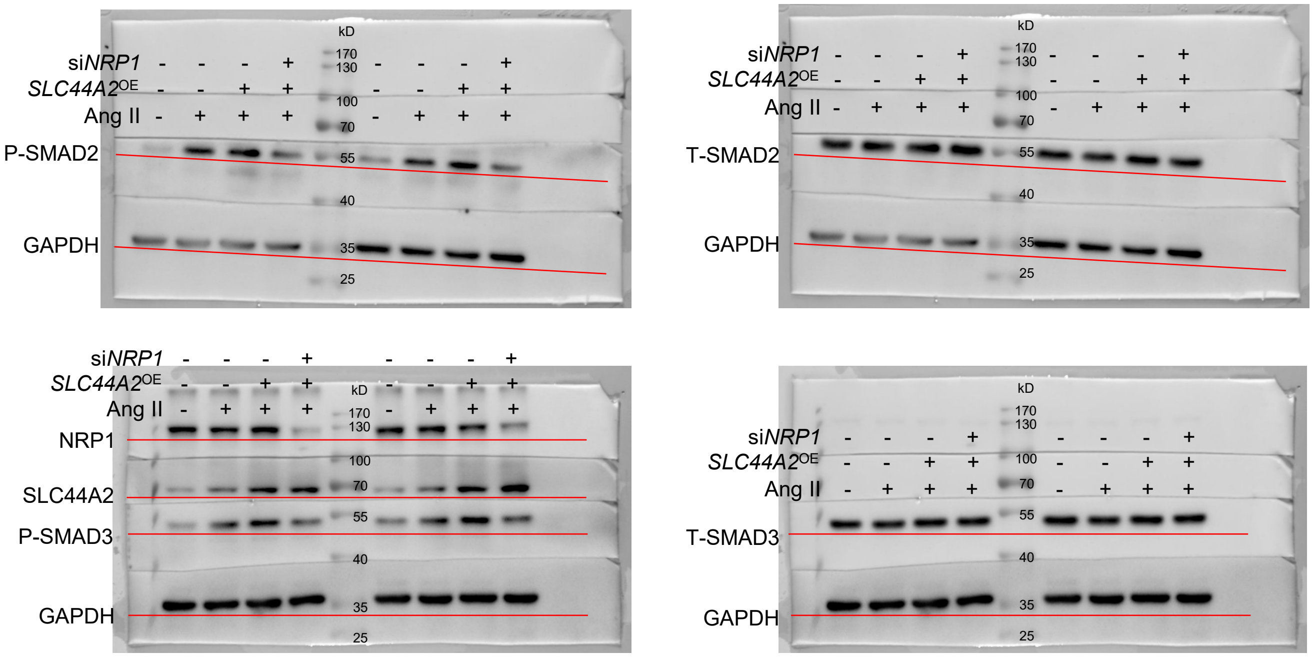

Full unedited gel for Figure 5G N=5

Figure 5G

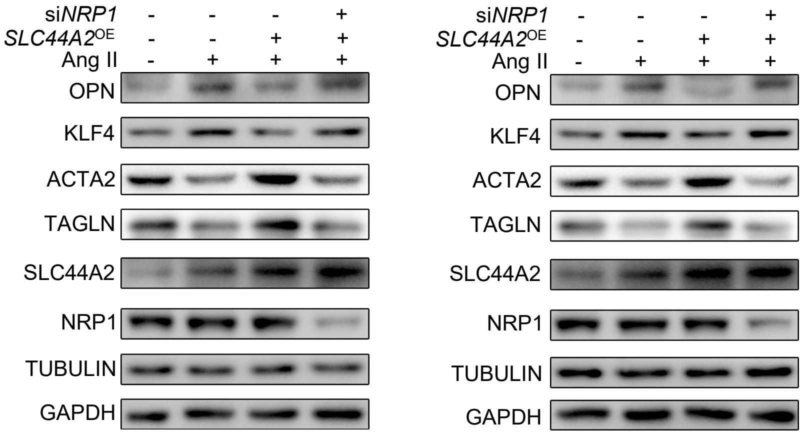

No. 1-2

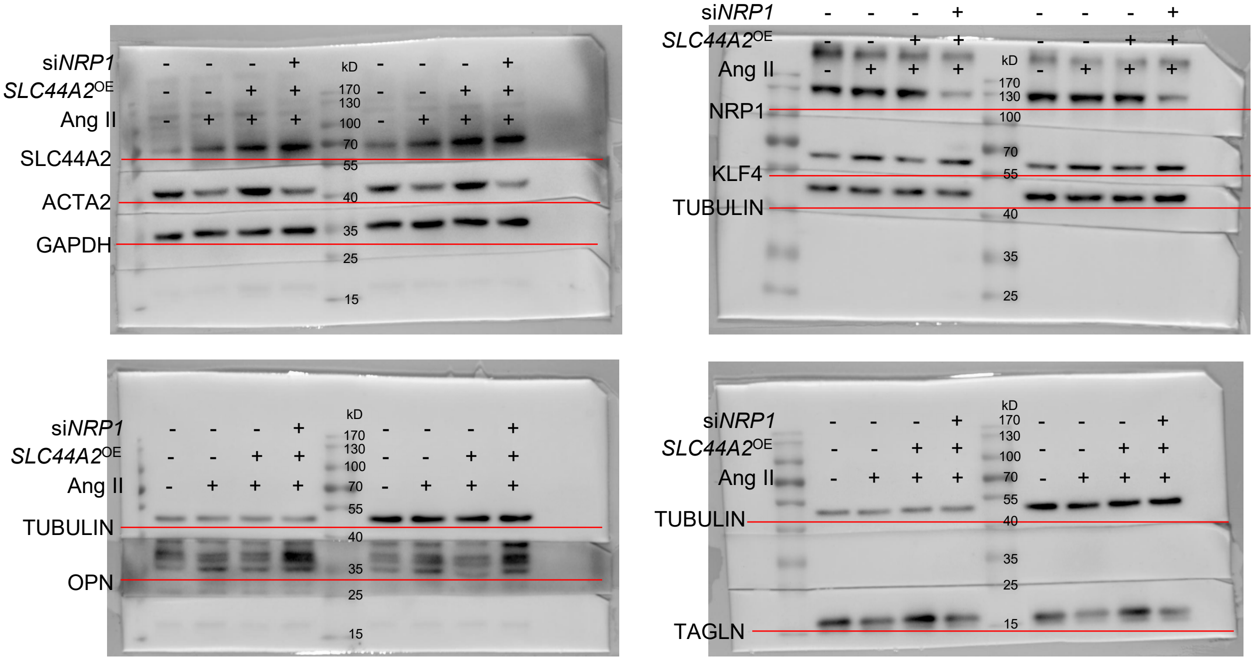

Full unedited gel for Figure 5G N=5

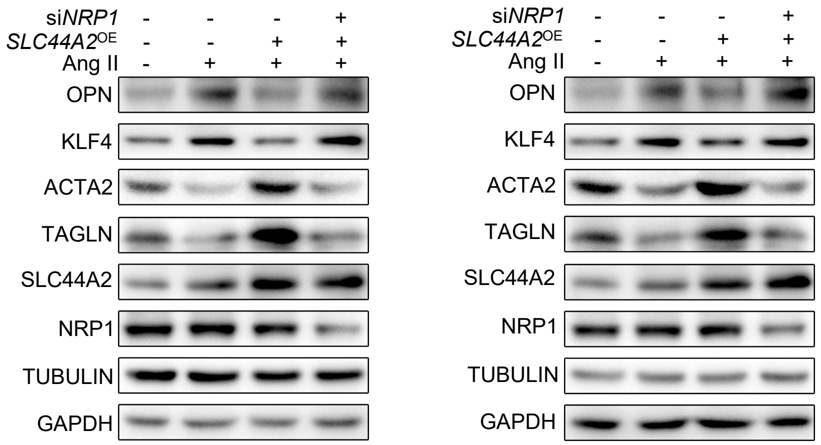

No. 3-4

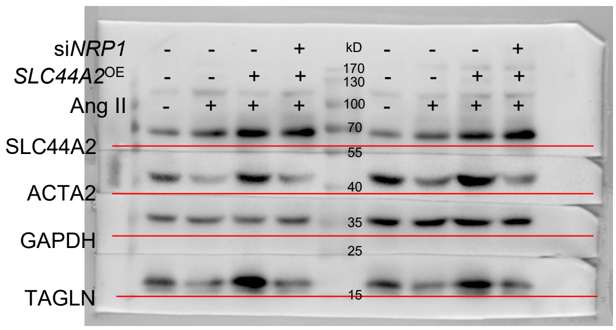

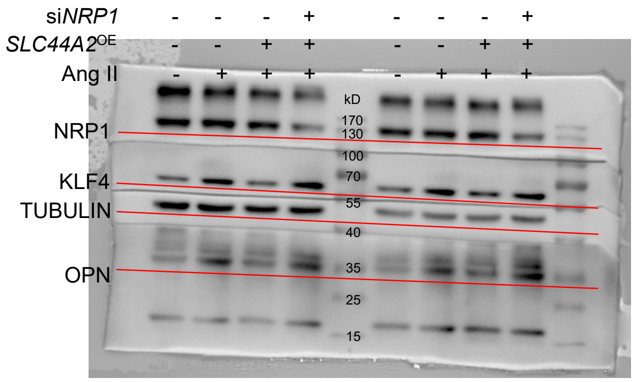

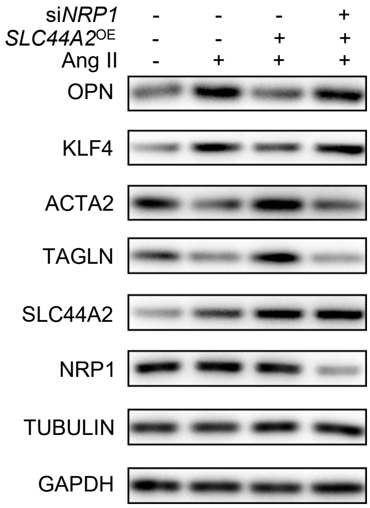

No. 5

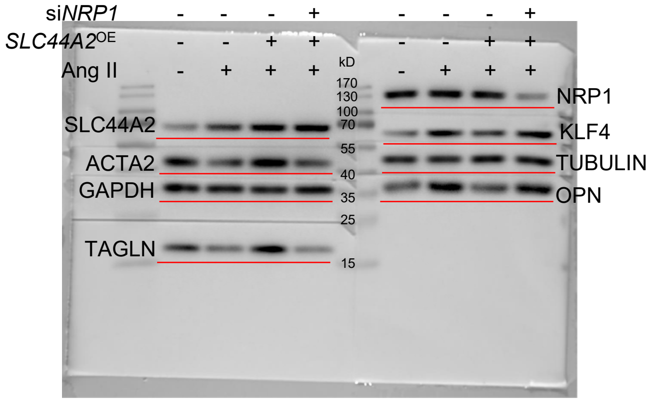

Full unedited gel for Figure 6A N=3

Figure 6A

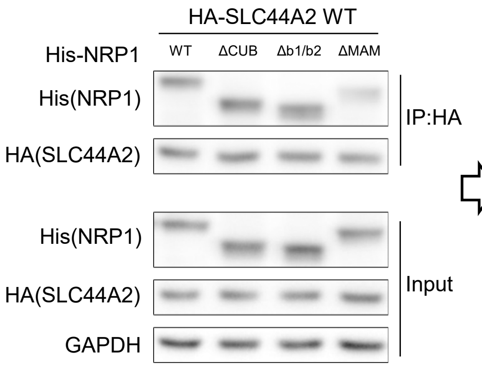

No. 1

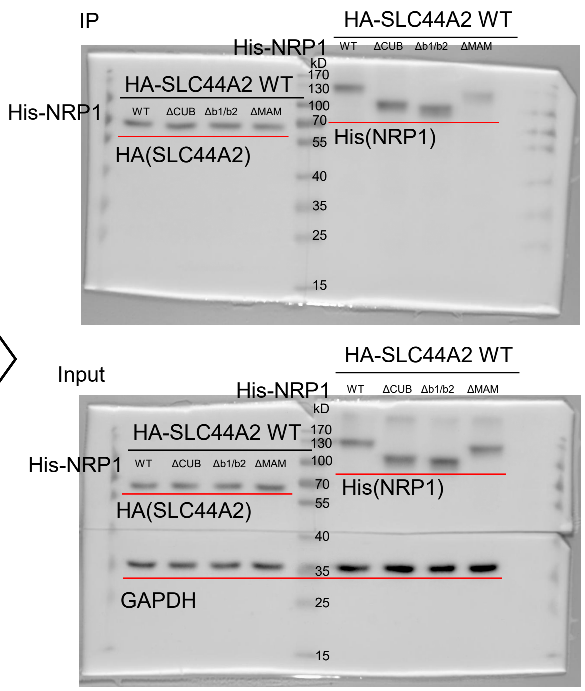

No. 2

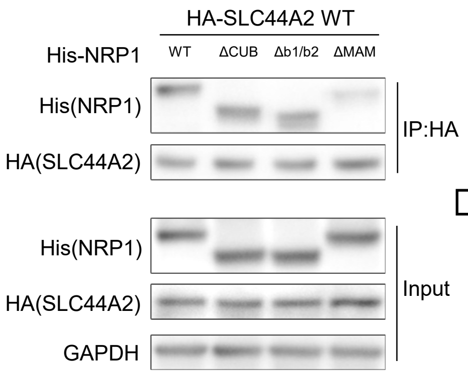

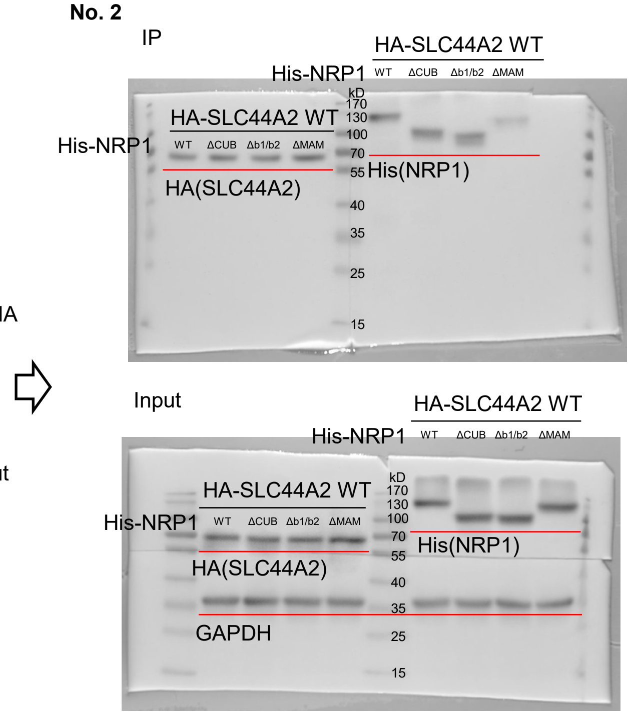

Full unedited gel for Figure 6A N=3

No. 3

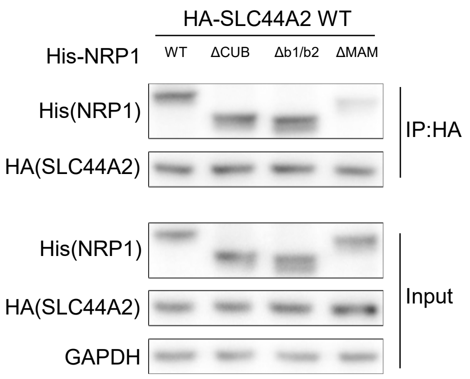

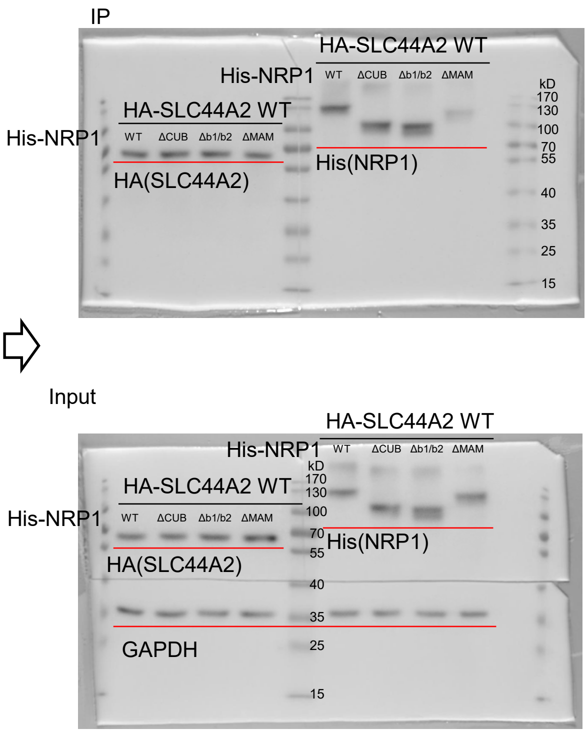

Full unedited gel for Figure 6B N=3

Figure 6B

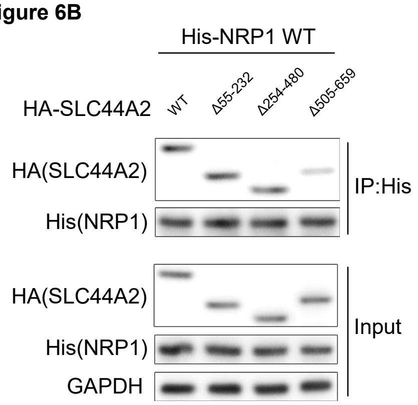

No. 1

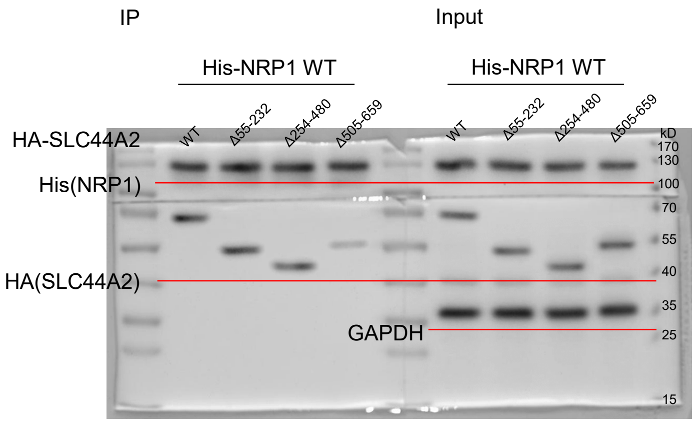

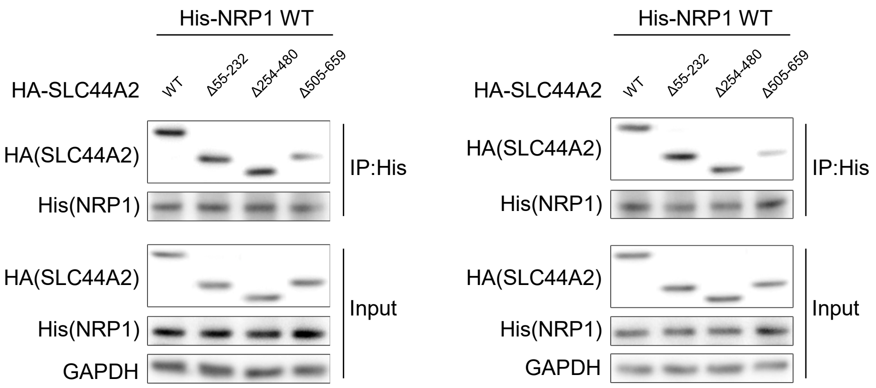

No. 2-3

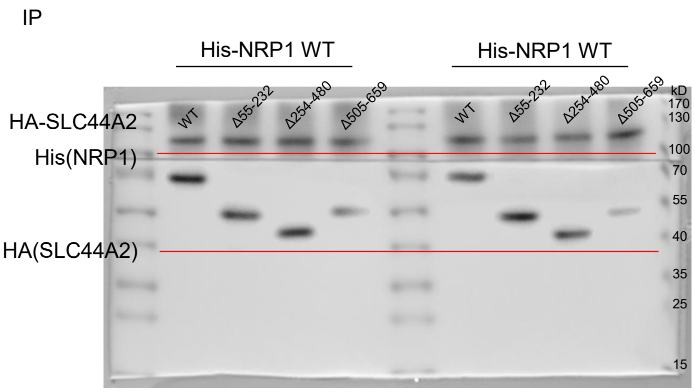

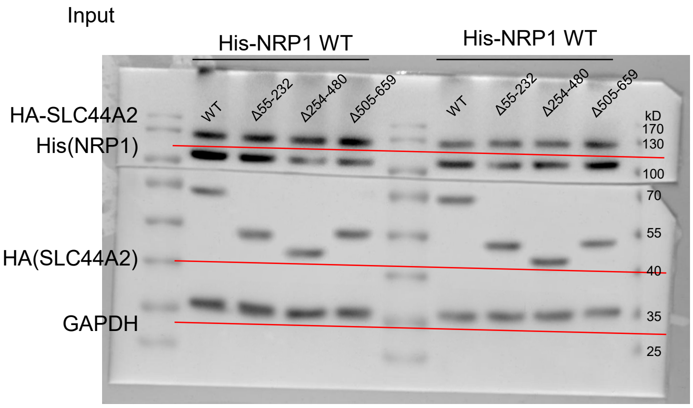

Full unedited gel for Figure 6E N=3

Figure 6E

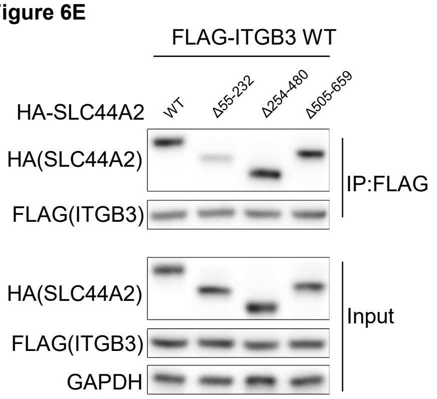

No. 1

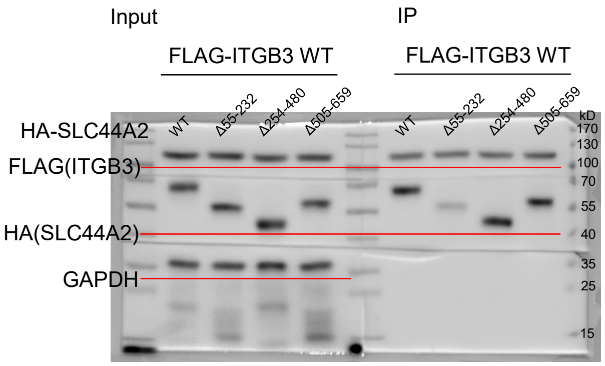

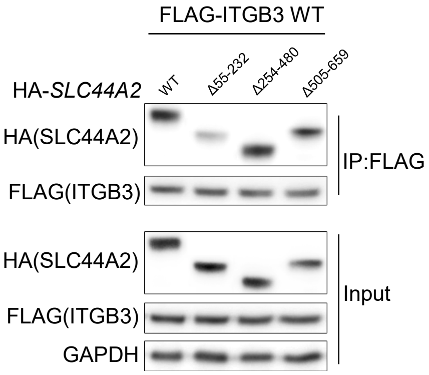

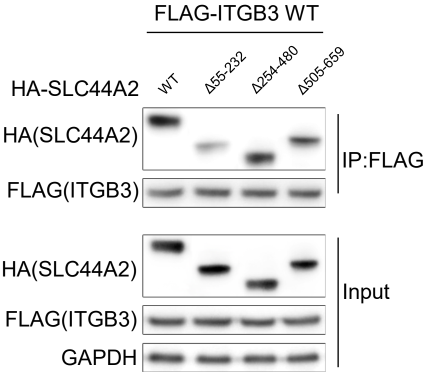

No. 2-3

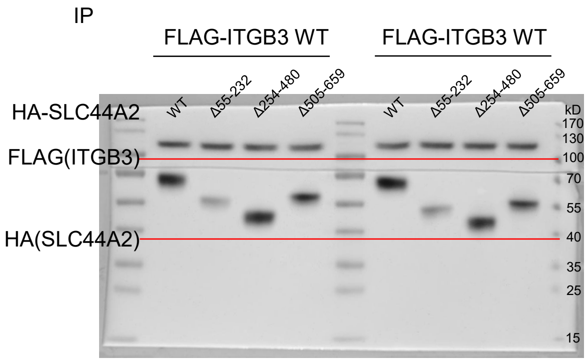

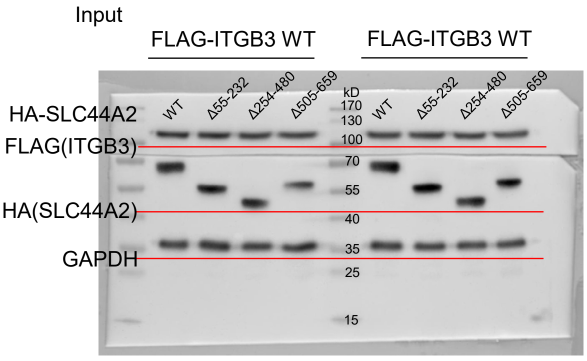

Full unedited gel for Figure 6I N=3

Figure 6I

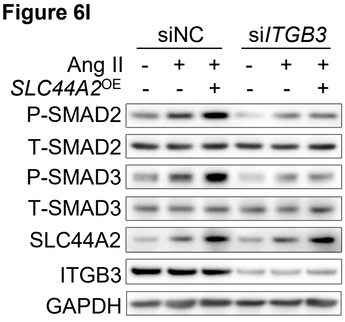

No. 1

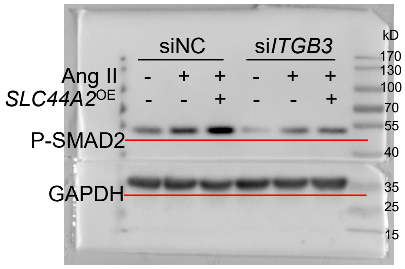

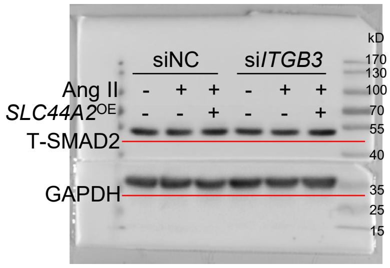

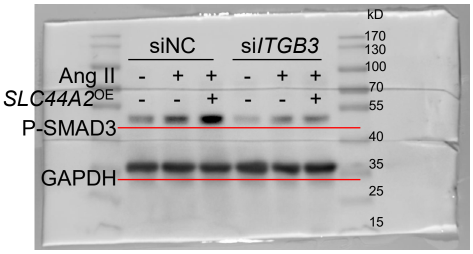

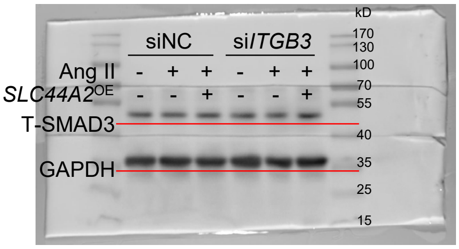

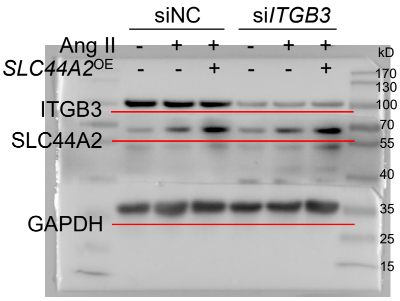

Full unedited gel for Figure 6I   N=3

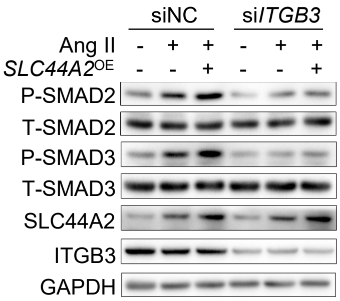

No. 2

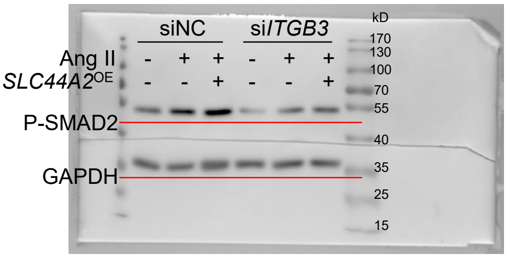

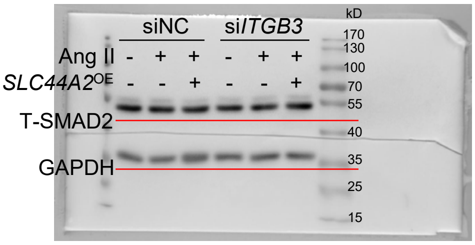

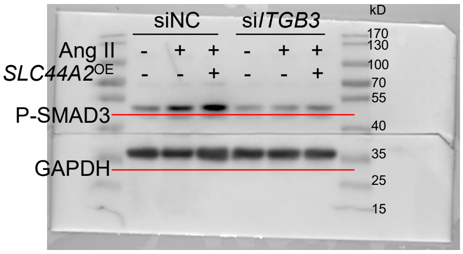

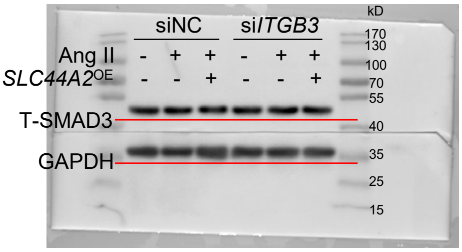

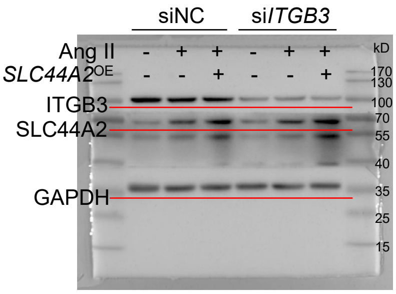

Full unedited gel for Figure 6I N=3

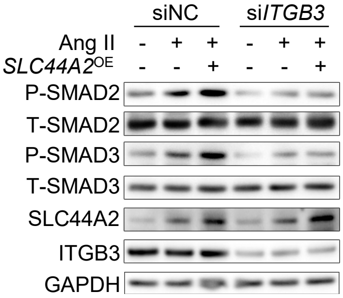

No. 3

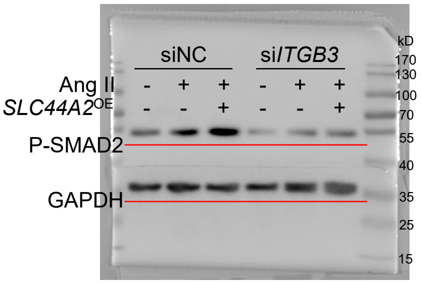

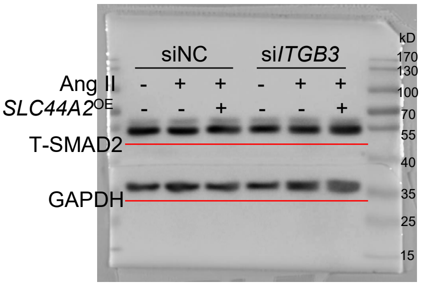

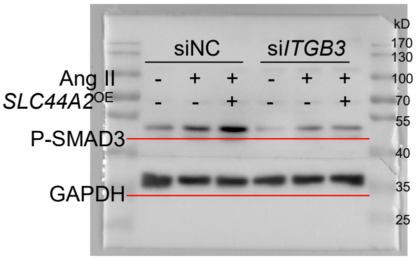

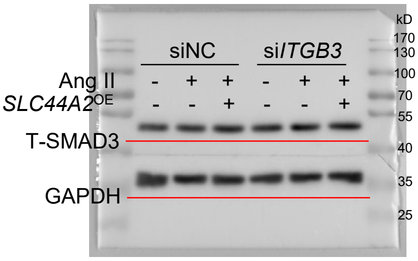

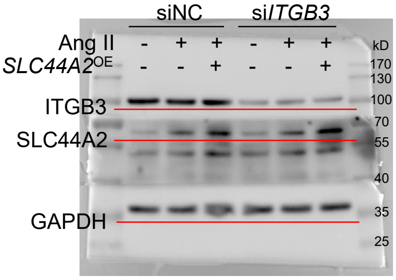

Full unedited gel for Figure 7B   N=5

Figure 7B

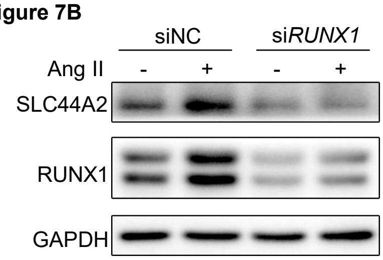

No. 1

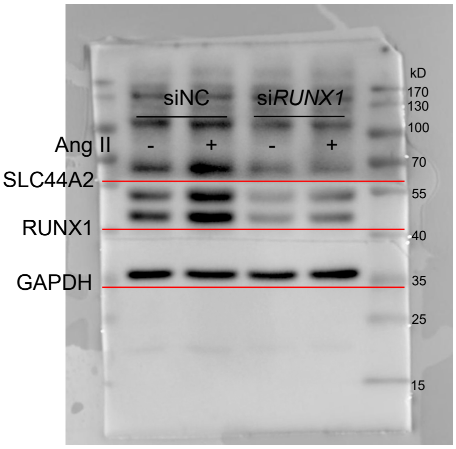

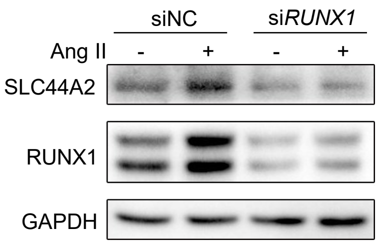

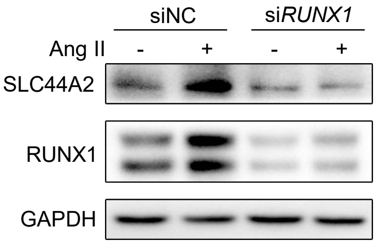

No. 2-3

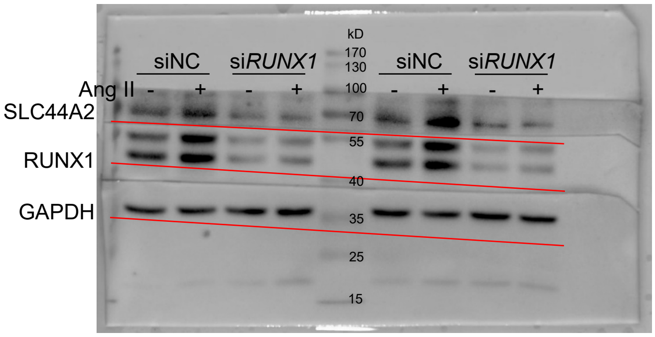

No. 4

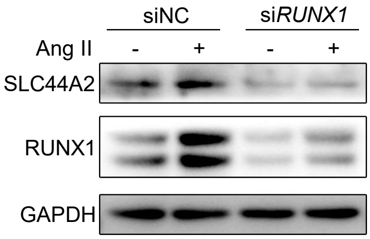

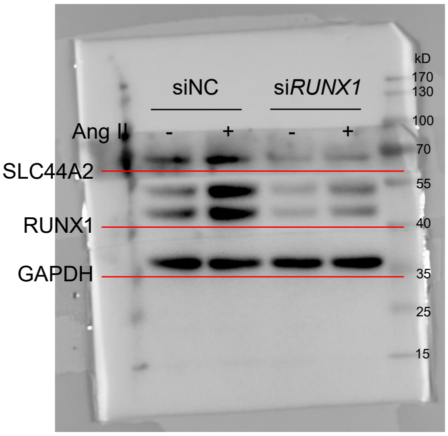

Full unedited gel for Figure 7B   N=5

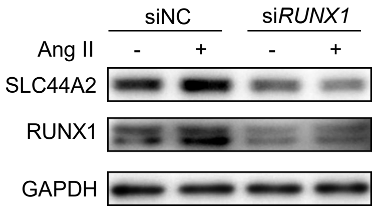

No. 5

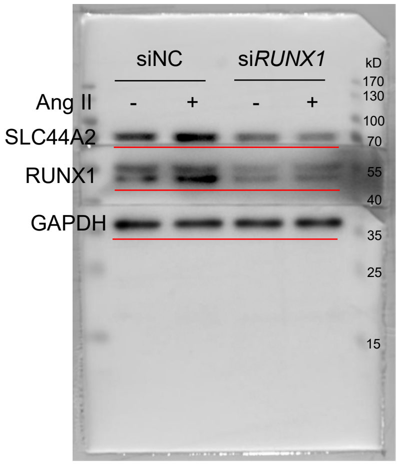

Full unedited gel for Figure 7C N=6

Figure 7C

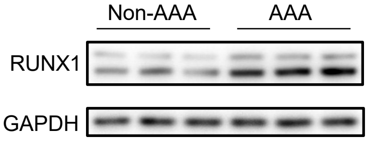

No. 1-3

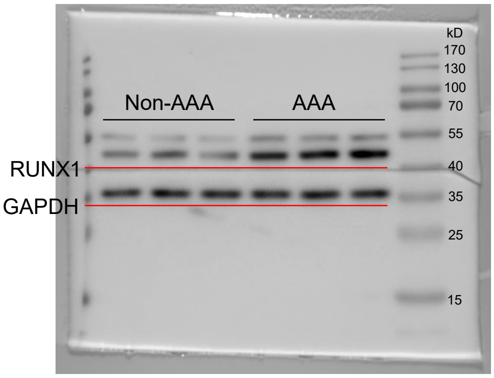

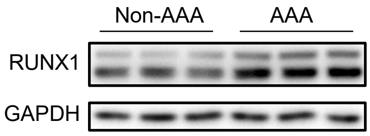

No. 4-6

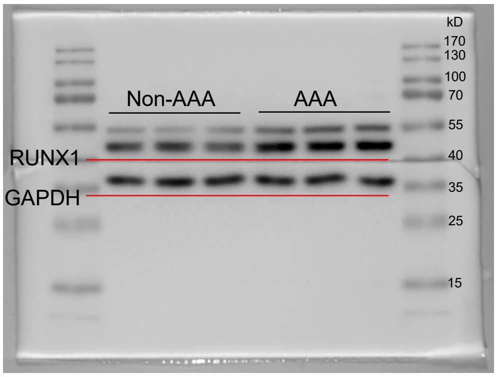

Full unedited gel for Supplemental Figure 1B N=6

No. 1-3

Supplemental Figure 1B

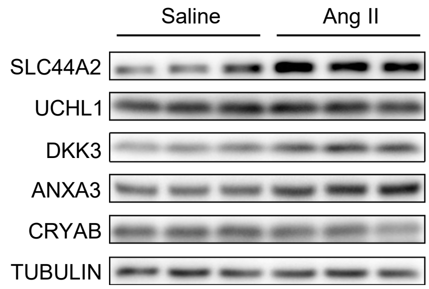

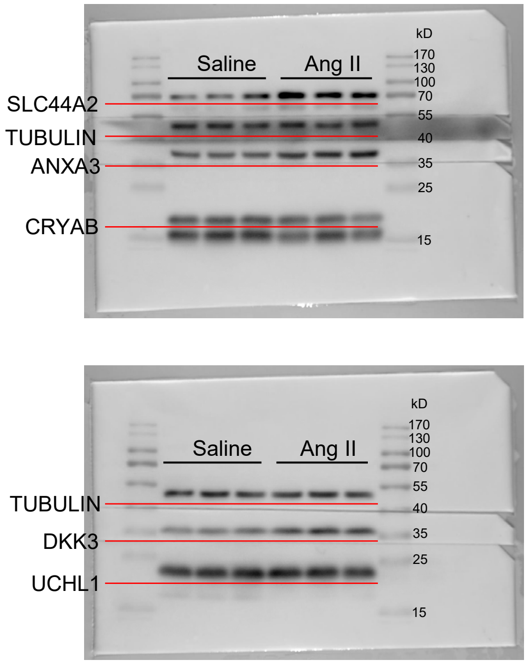

No. 4-6

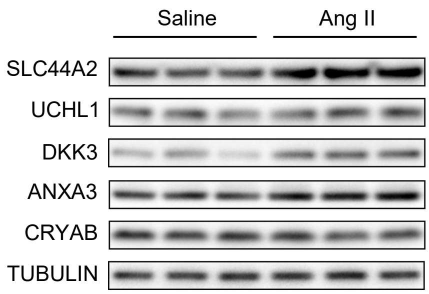

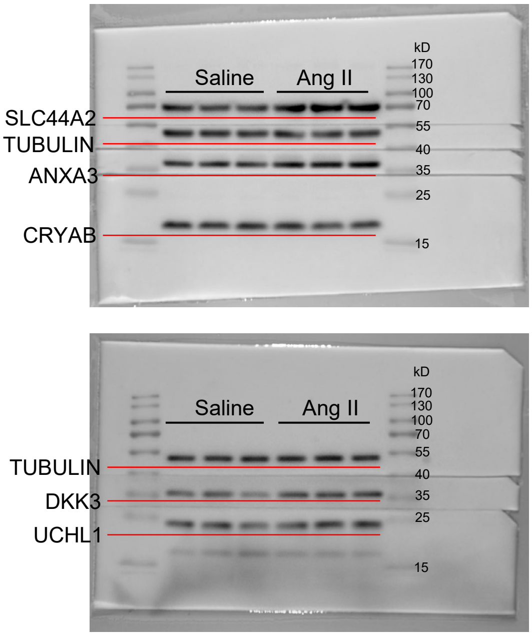

Full unedited gel for Supplemental Figure 1C N=5

Supplemental Figure 1C

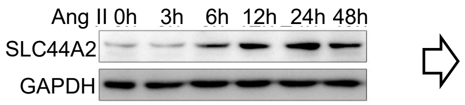

No. 1

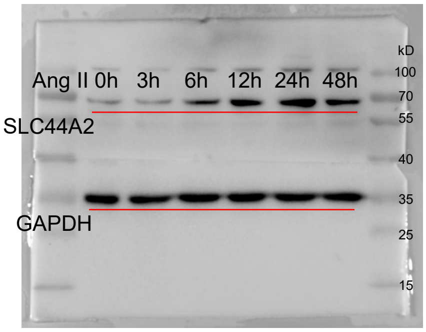

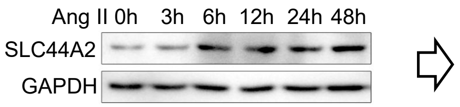

No. 2

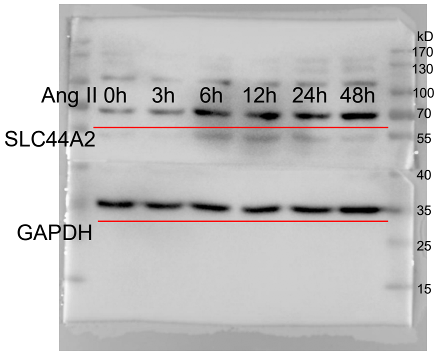

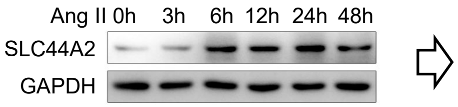

No. 3

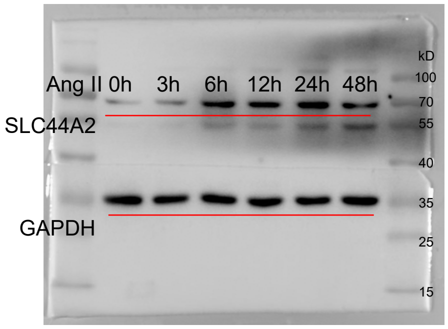

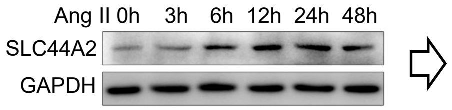

No. 4

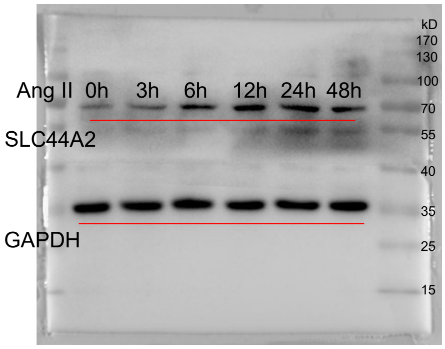

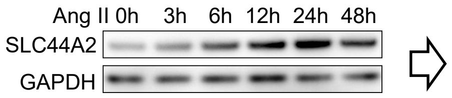

No. 5

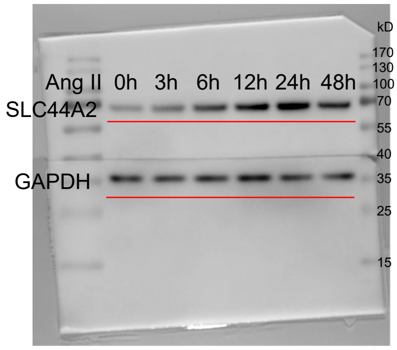

Full unedited gel for Supplemental Figure 2A   N=5

Supplemental Figure 2A

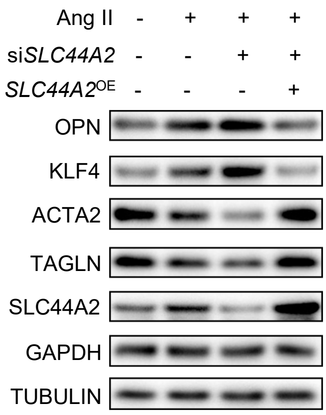

No. 1

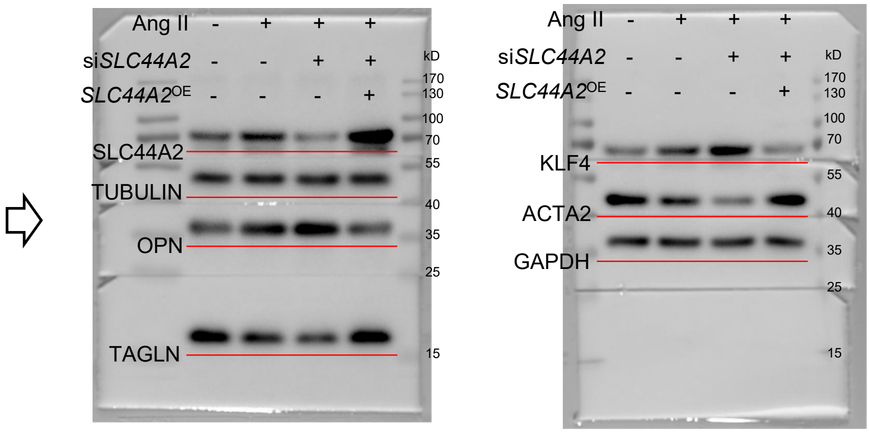

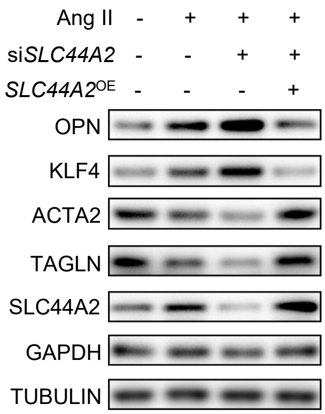

No. 2

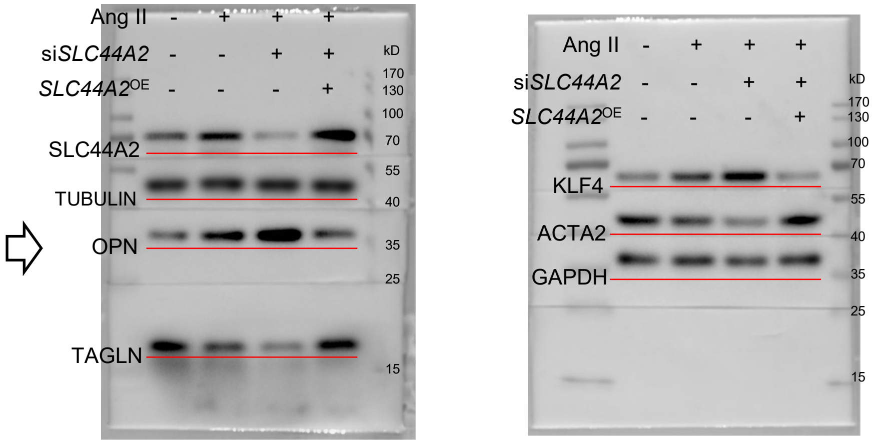

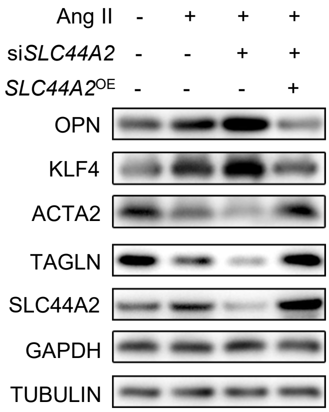

No. 3

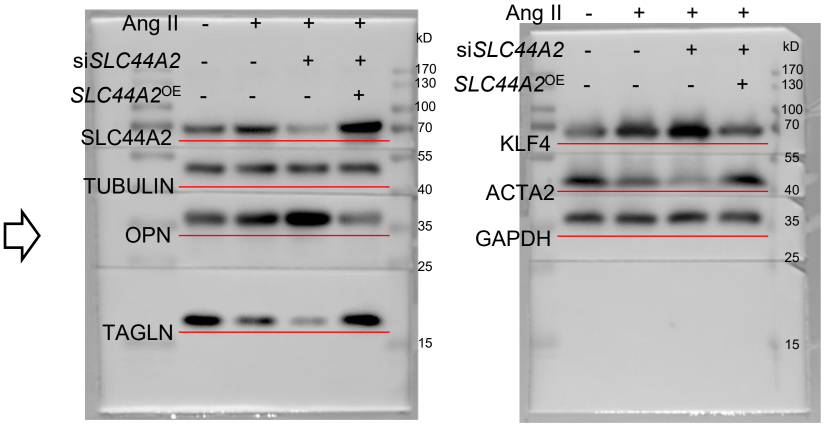

Full unedited gel for Supplemental Figure 2A   N=5

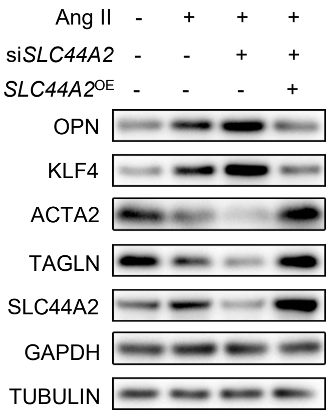

No. 4

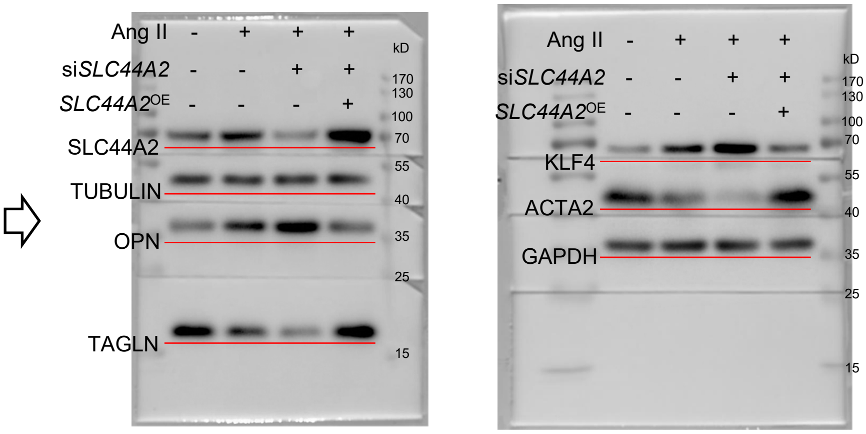

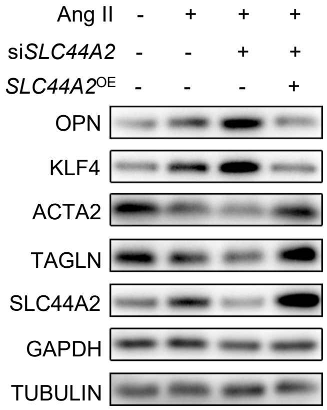

No. 5

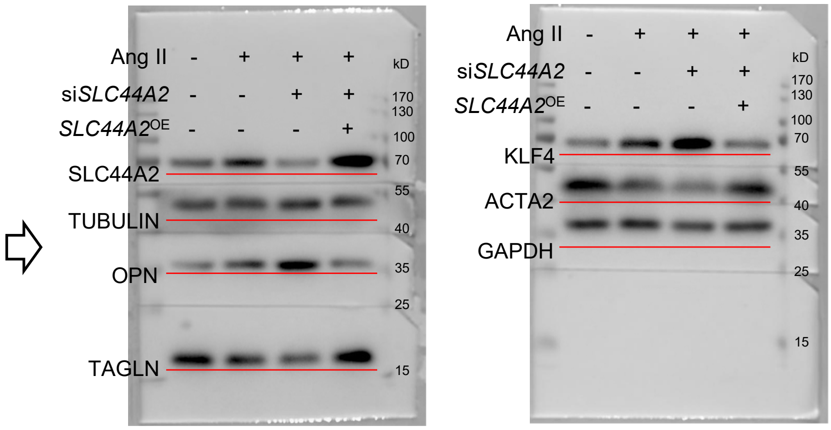

Full unedited gel for Supplemental Figure 4A   N=3

Supplemental Figure 4A

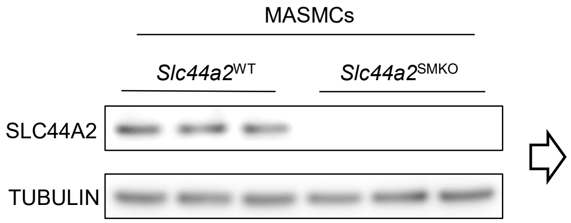

No. 1-3

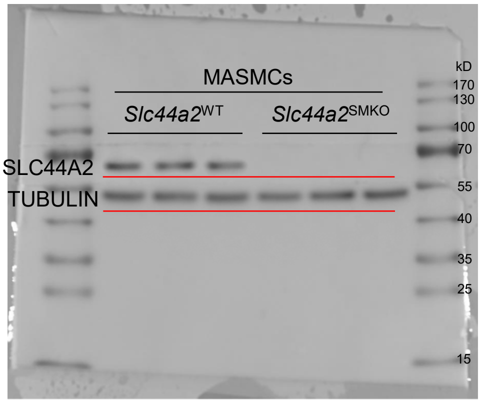

Full unedited gel for Supplemental Figure 4E N=6

No. 1-2

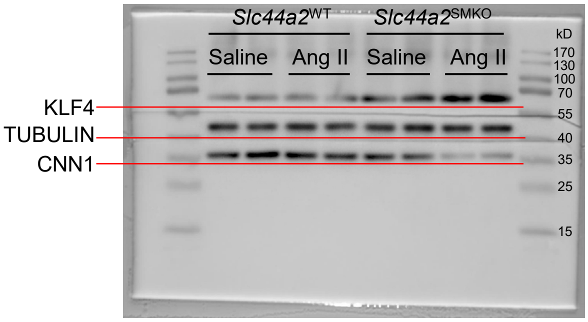

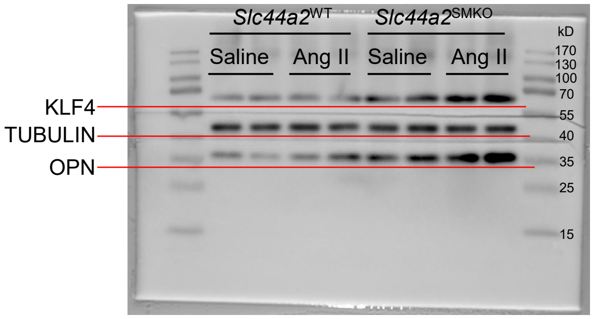

Supplemental Figure 4E

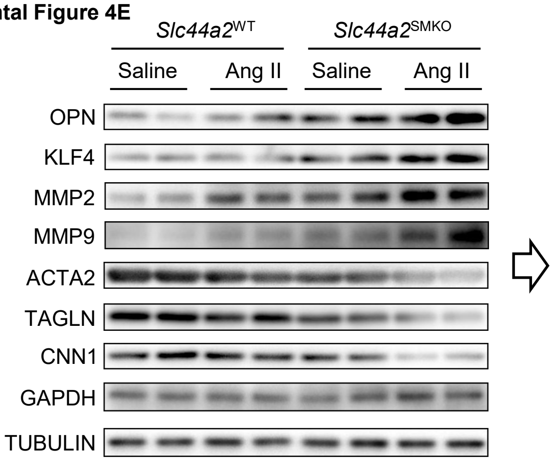

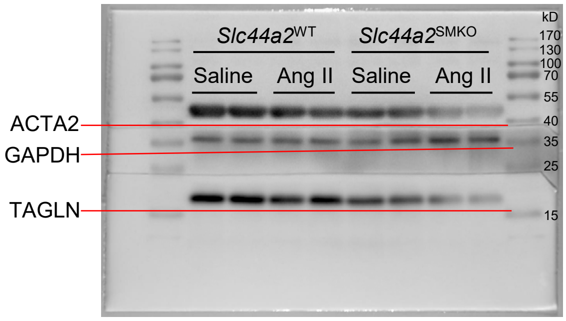

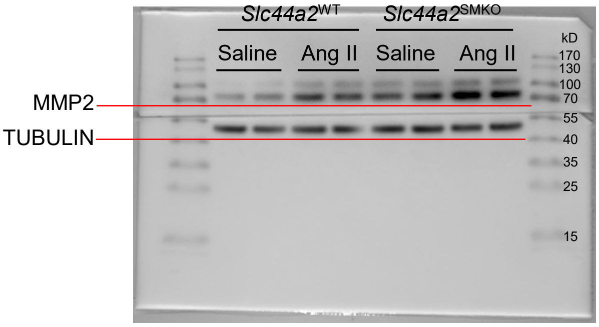

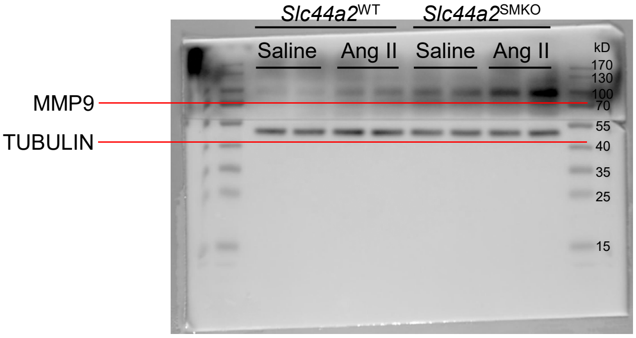

Full unedited gel for Supplemental Figure 4E N=6

No. 3-4

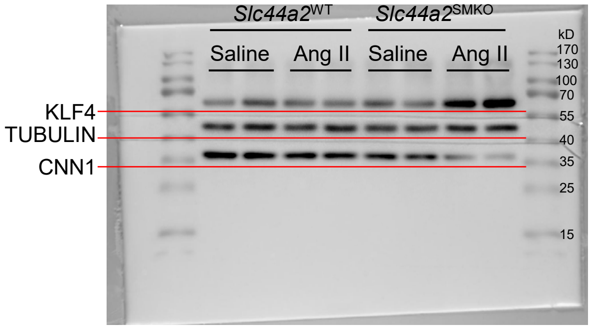

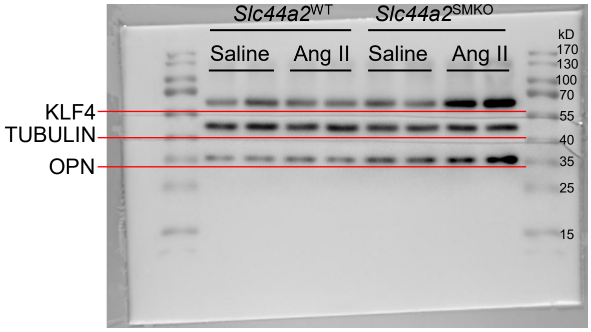

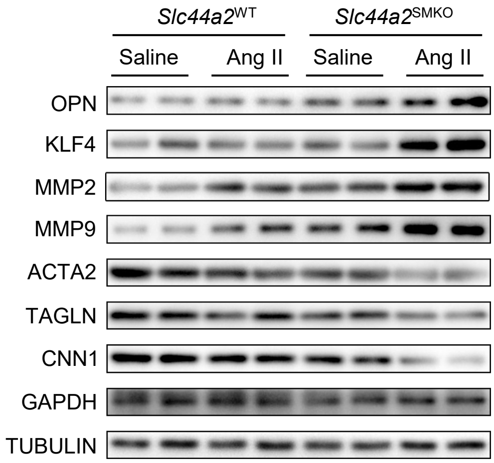

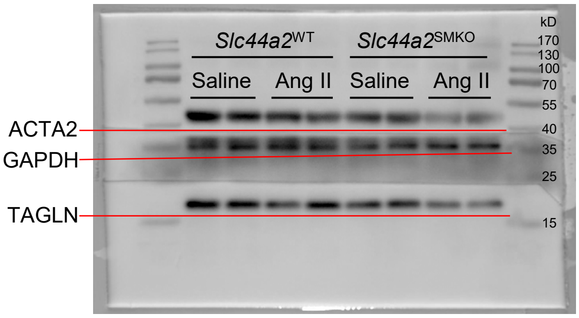

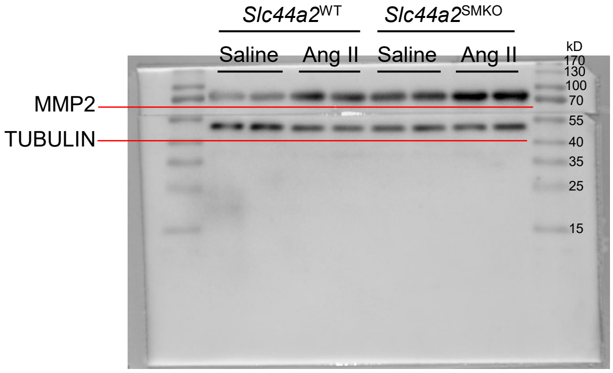

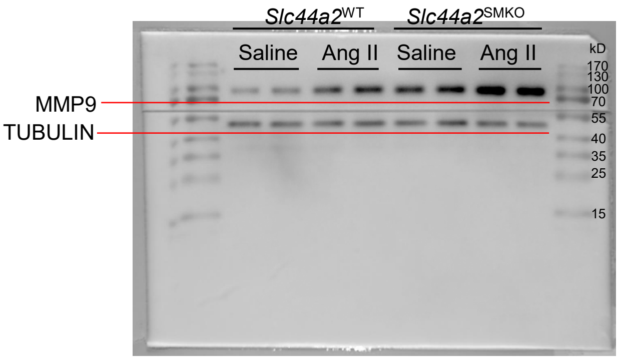

Full unedited gel for Supplemental Figure 4E   N=6

No. 5-6

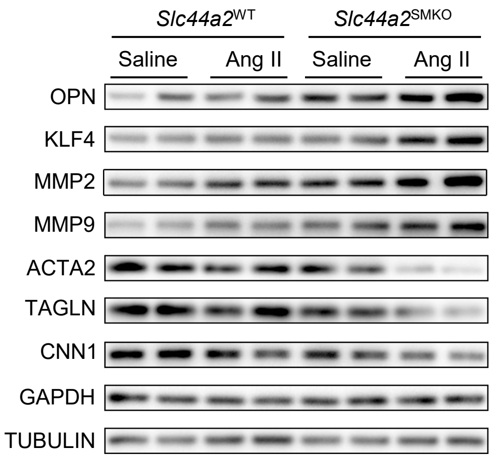

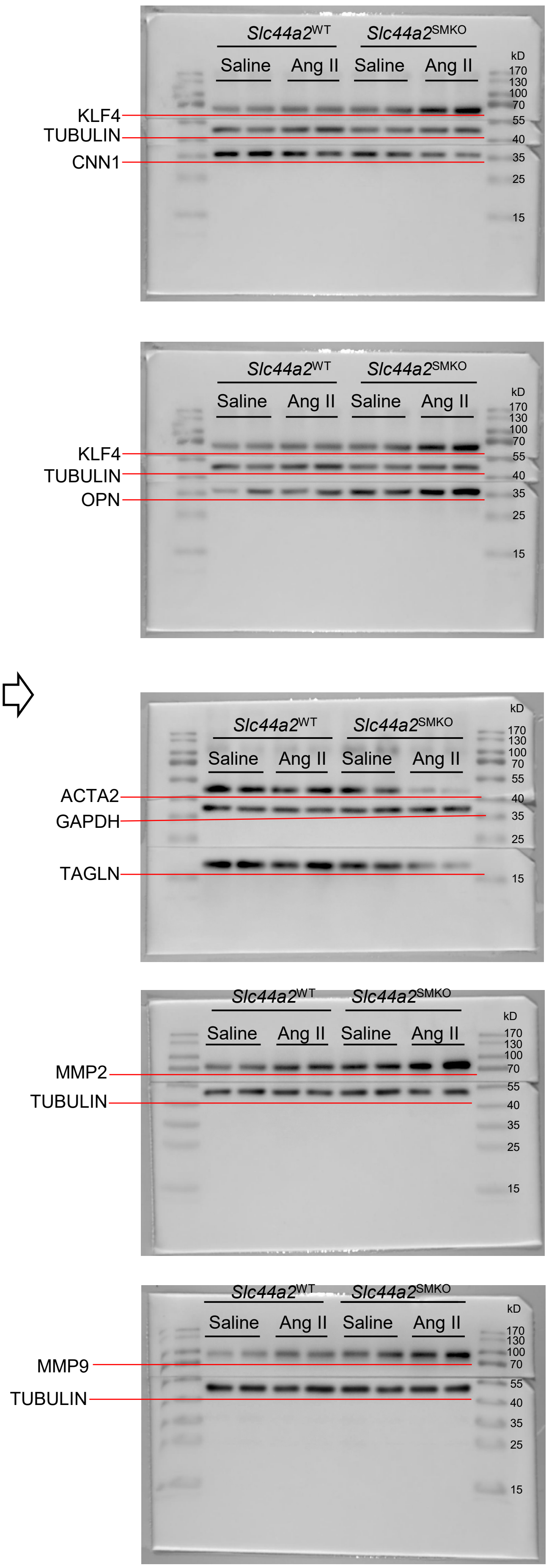

Full unedited gel for Supplemental Figure 6B N=5

Supplemental Figure 6B

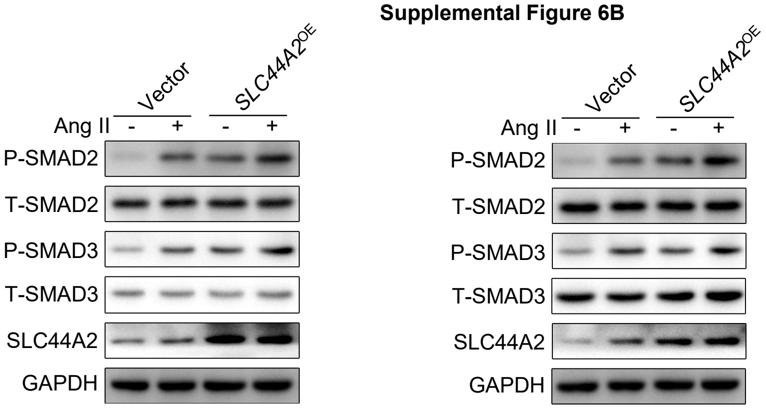

No. 1-2

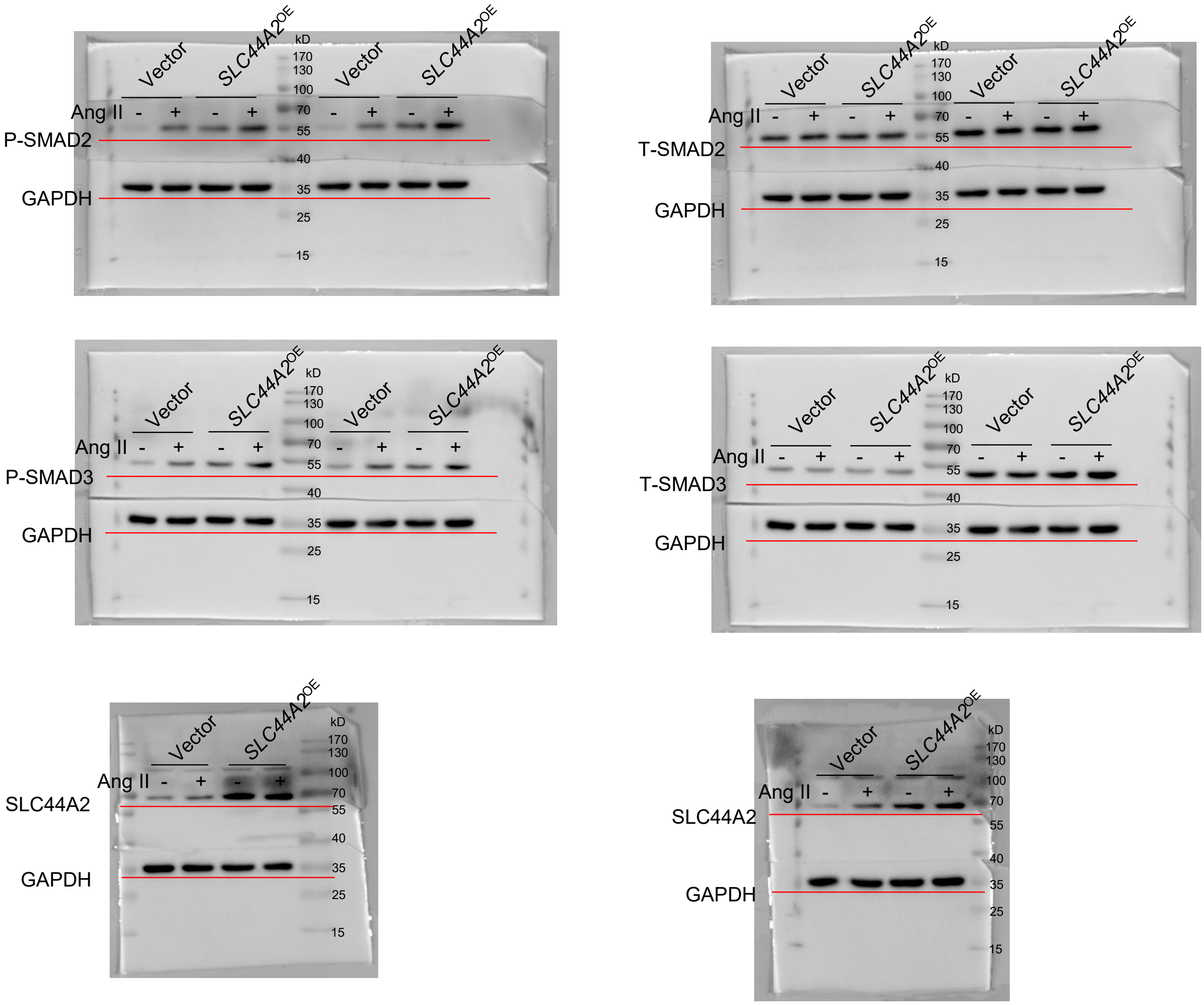

Full unedited gel for Supplemental Figure 6B N=5

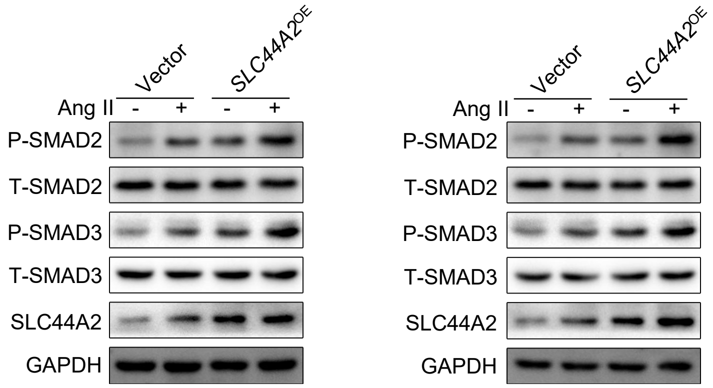

No. 3-4

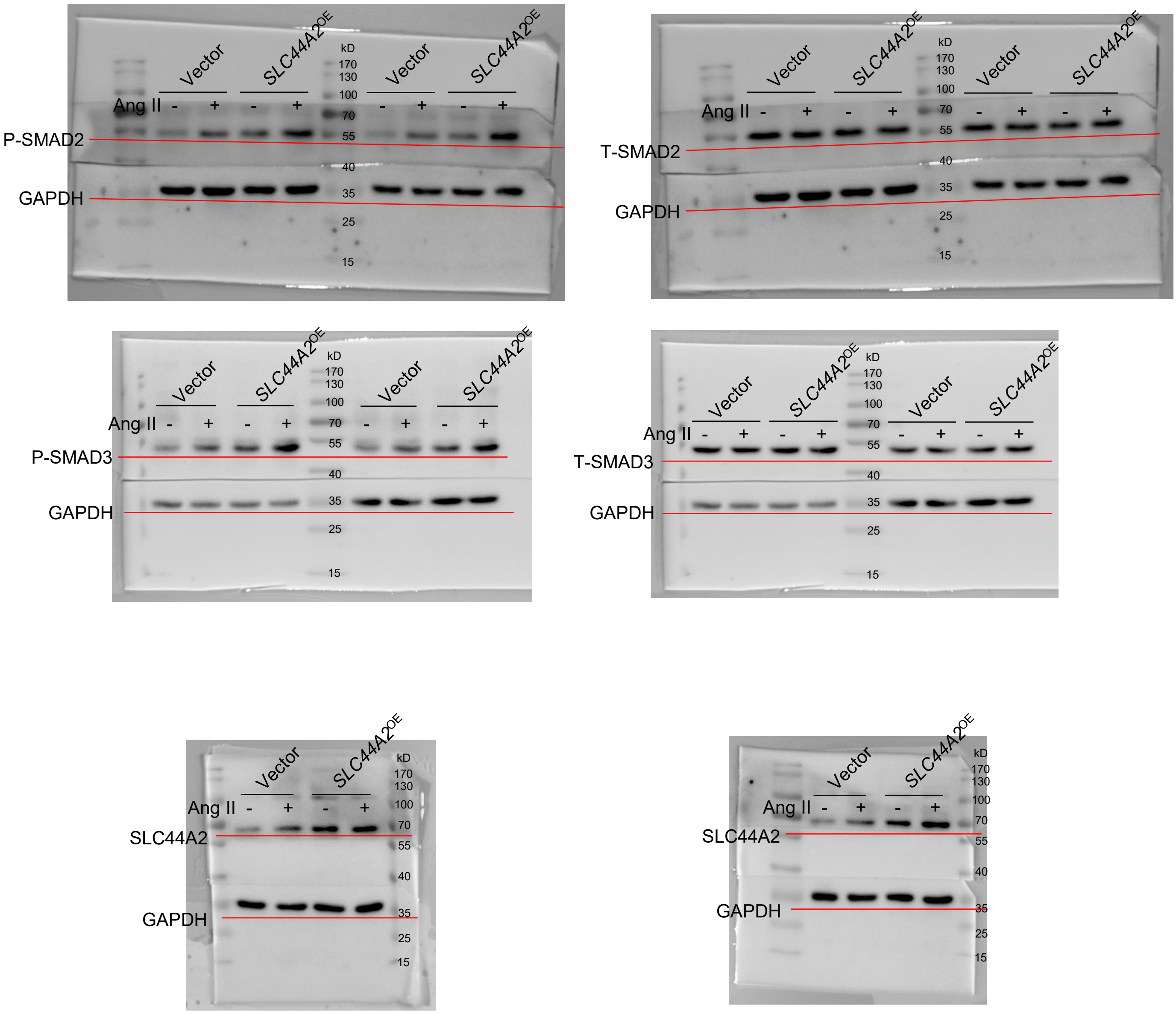

Full unedited gel for Supplemental Figure 6B N=5

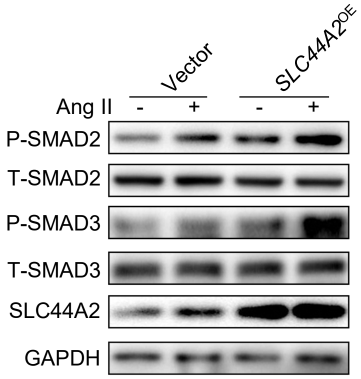

No. 5

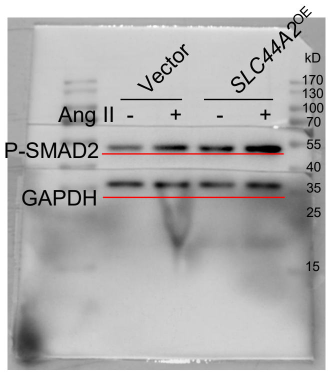

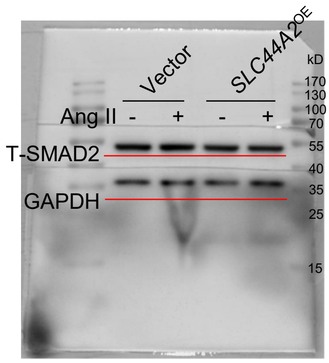

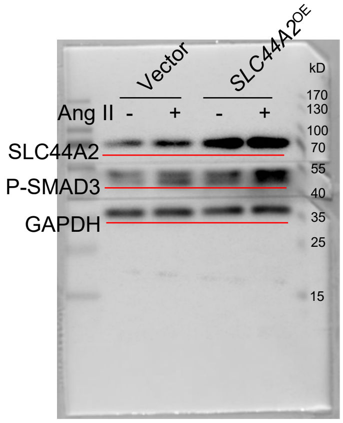

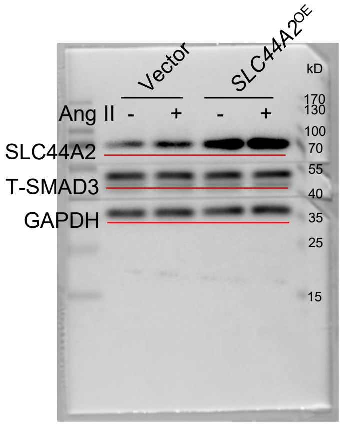

Full unedited gel for Supplement Figure 6D N=5

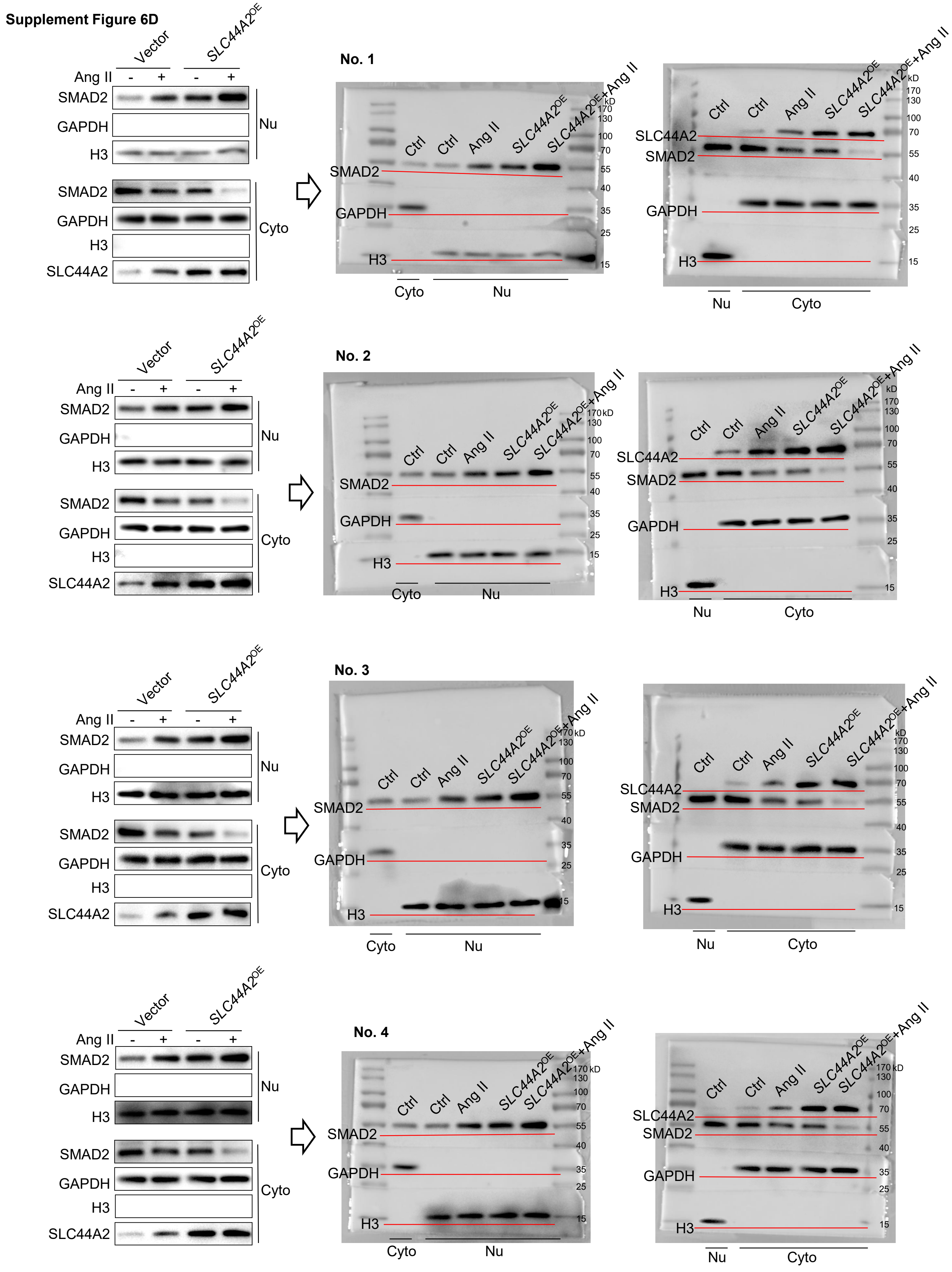

Full unedited gel for Supplement Figure 6D N=5

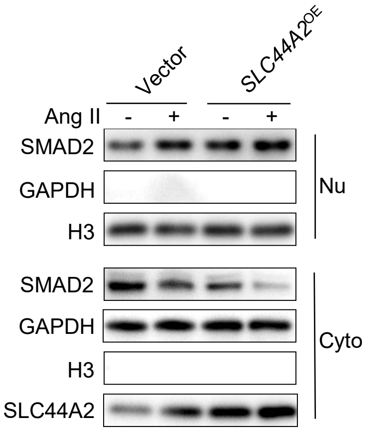

No. 5

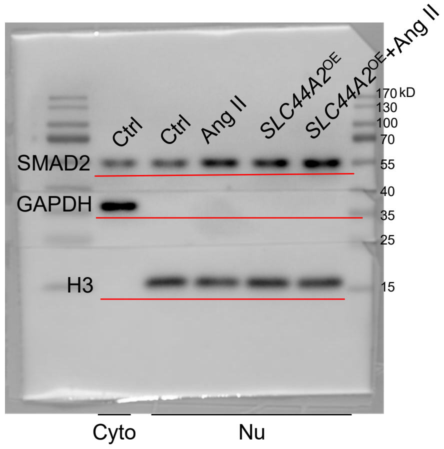

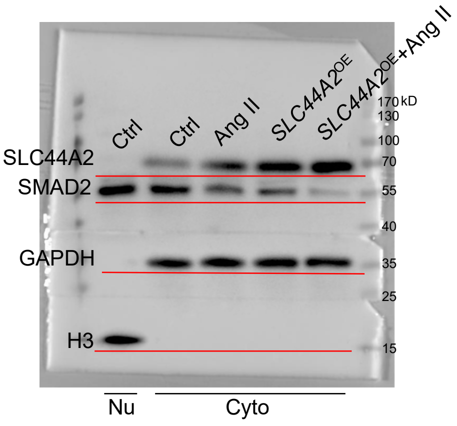

Full unedited gel for Supplement Figure 6F N=6

Supplement Figure 6F

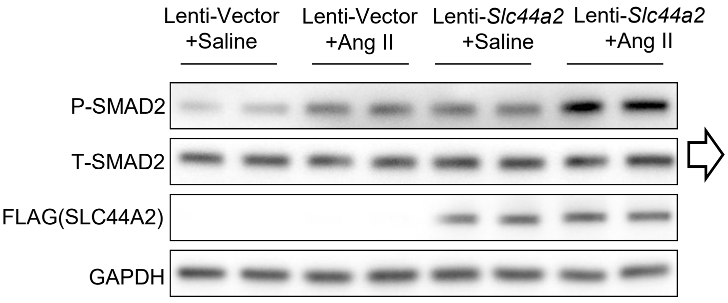

No. 1-2

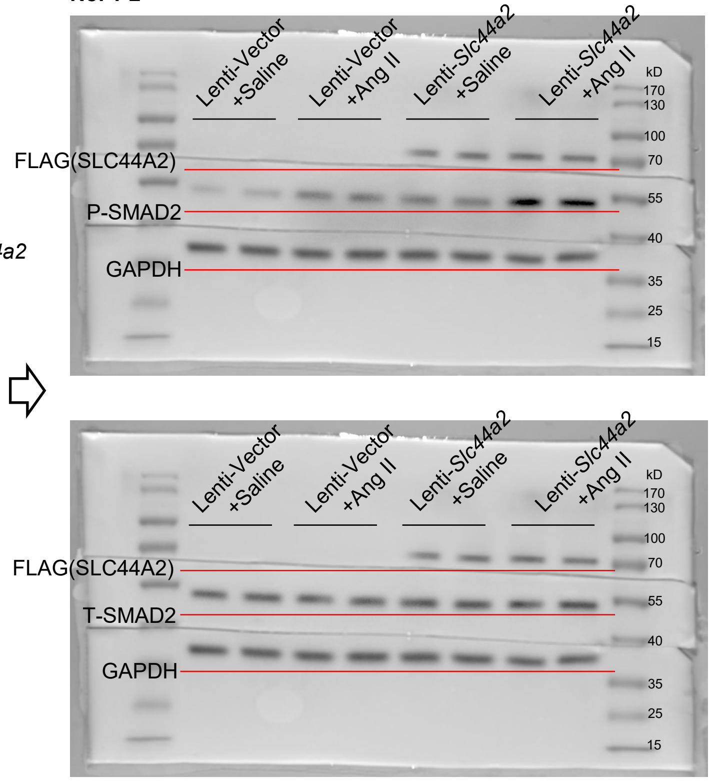

No. 3-4

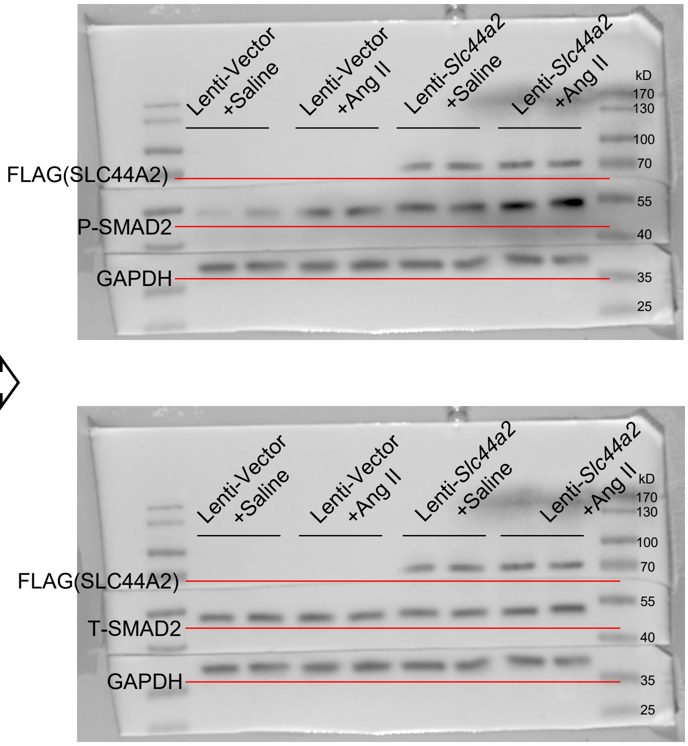

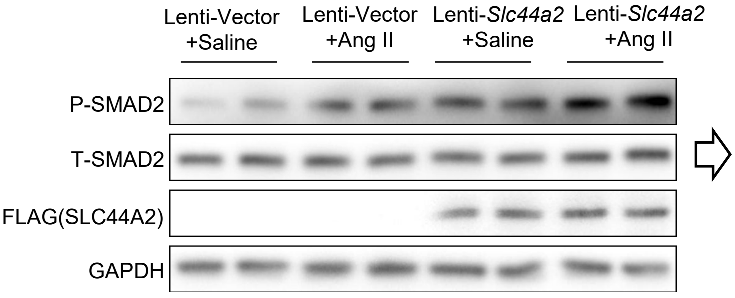

Full unedited gel for Supplement Figure 6F   N=6

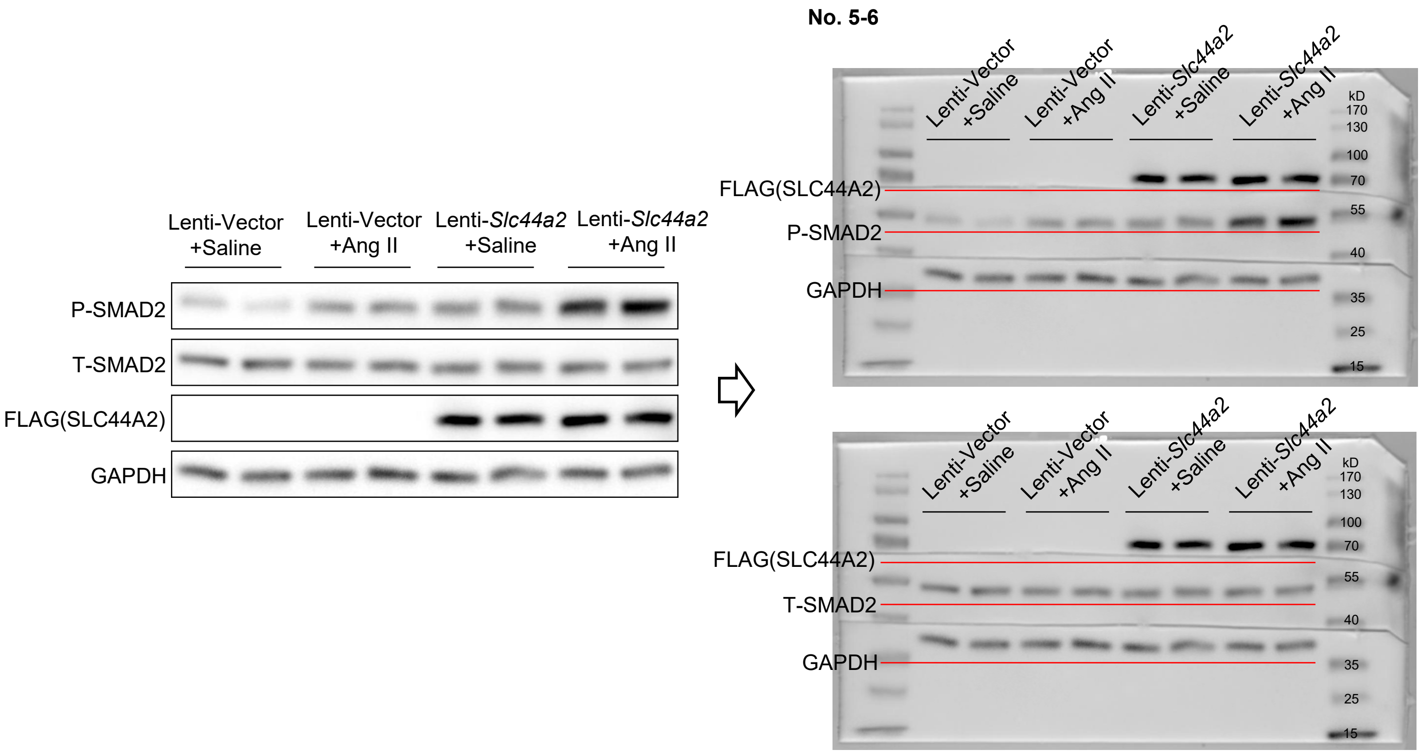

Full unedited gel for Supplement Figure 6H N=5

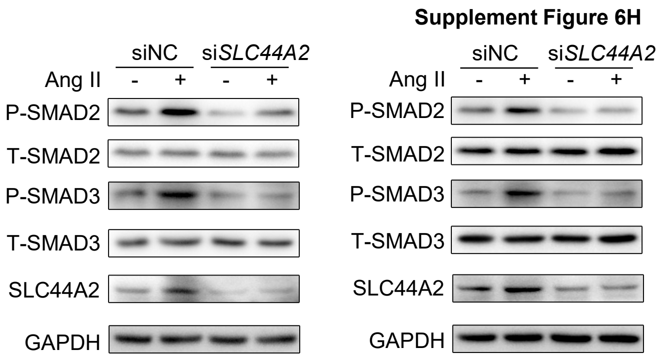

No. 1-2

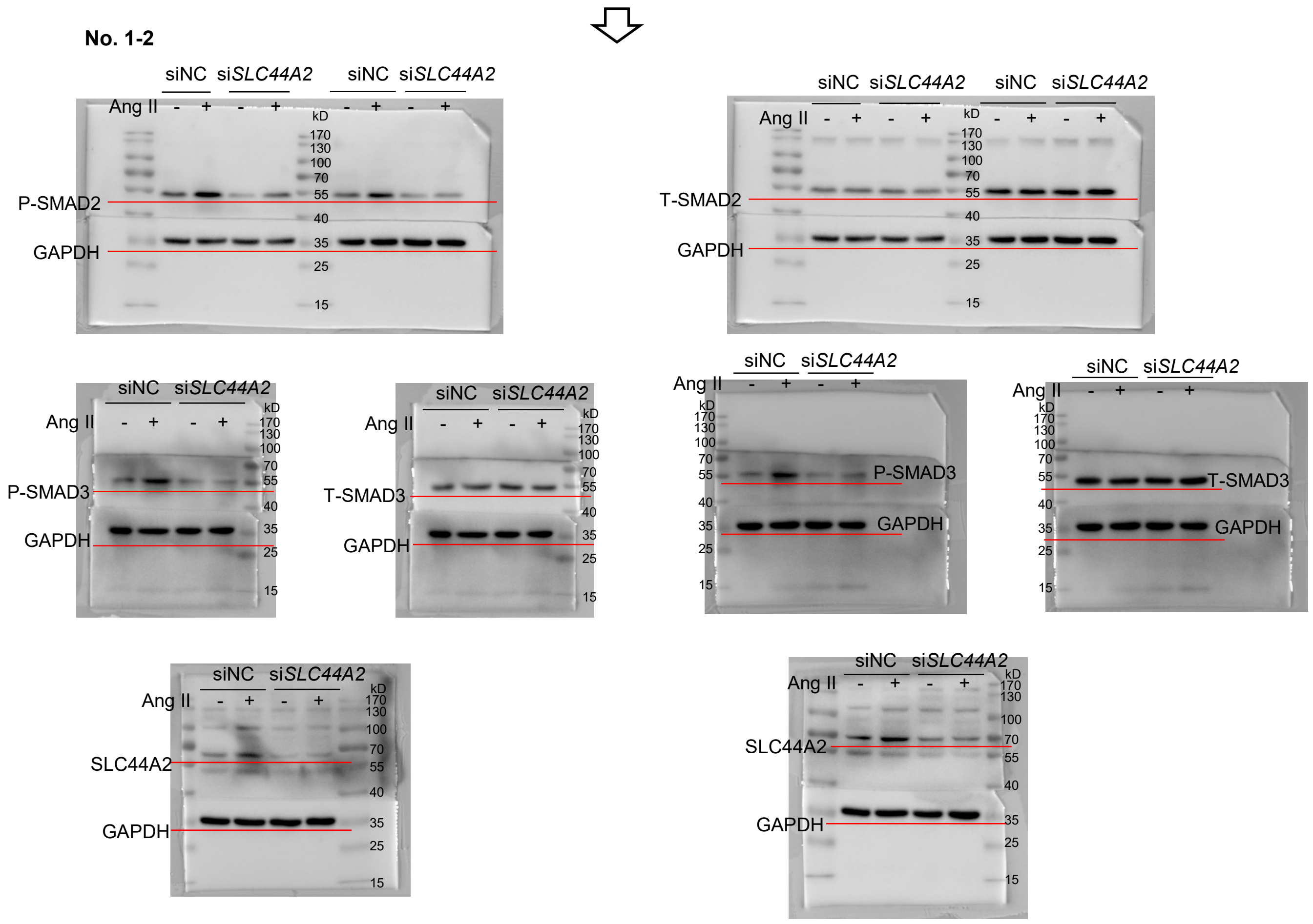

Full unedited gel for Supplement Figure 6H N=5

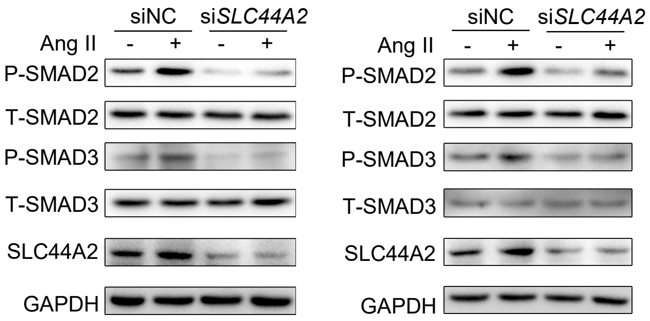

No. 3-4

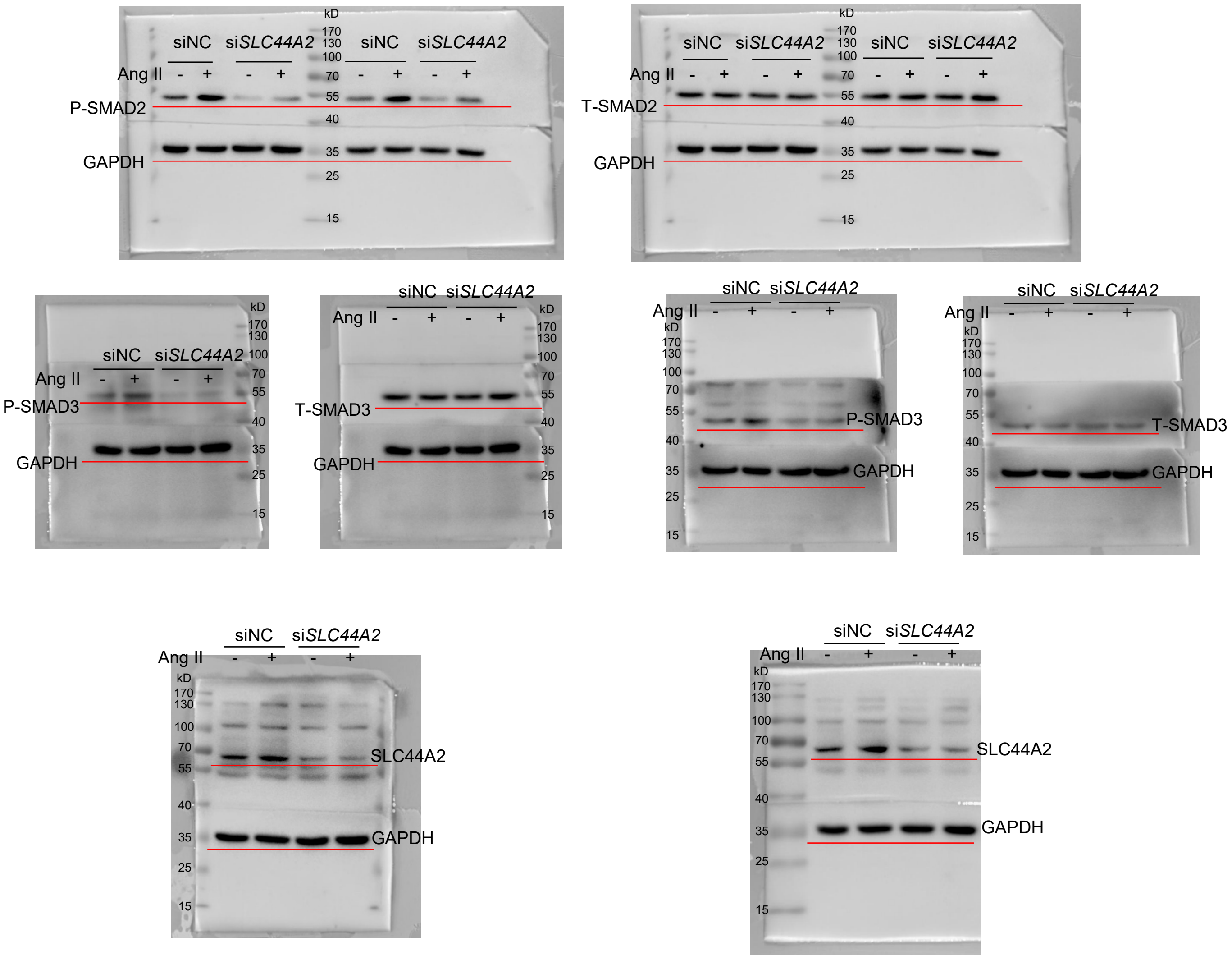

Full unedited gel for Supplement Figure 6H   N=5

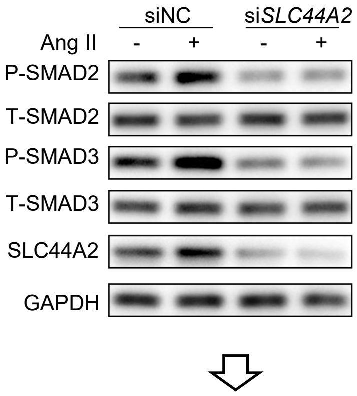

No. 5

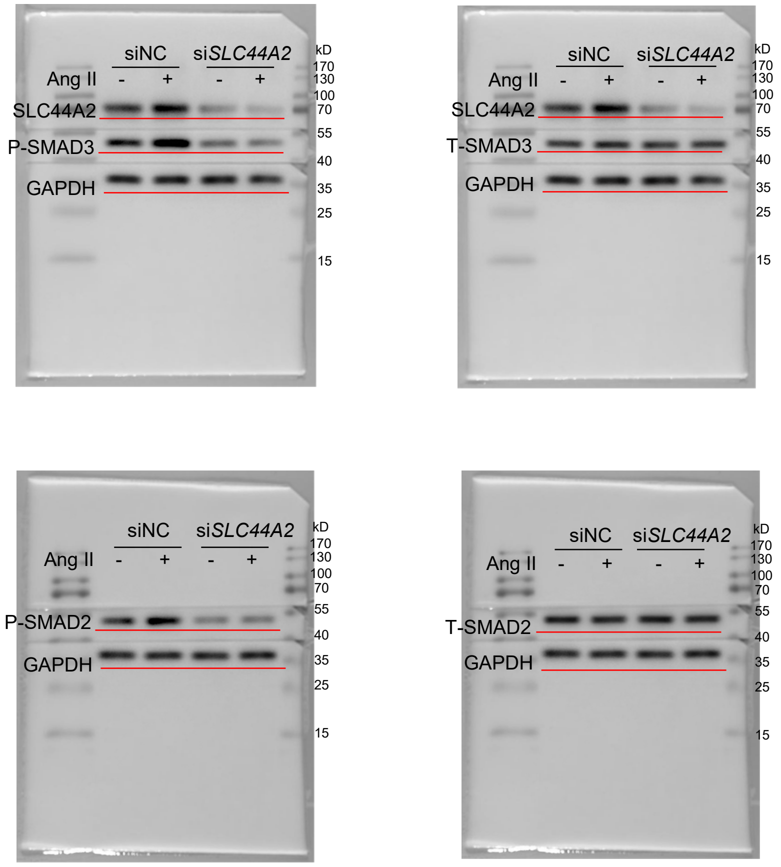

Full unedited gel for Supplement Figure 7A N=3

Supplement Figure 7A

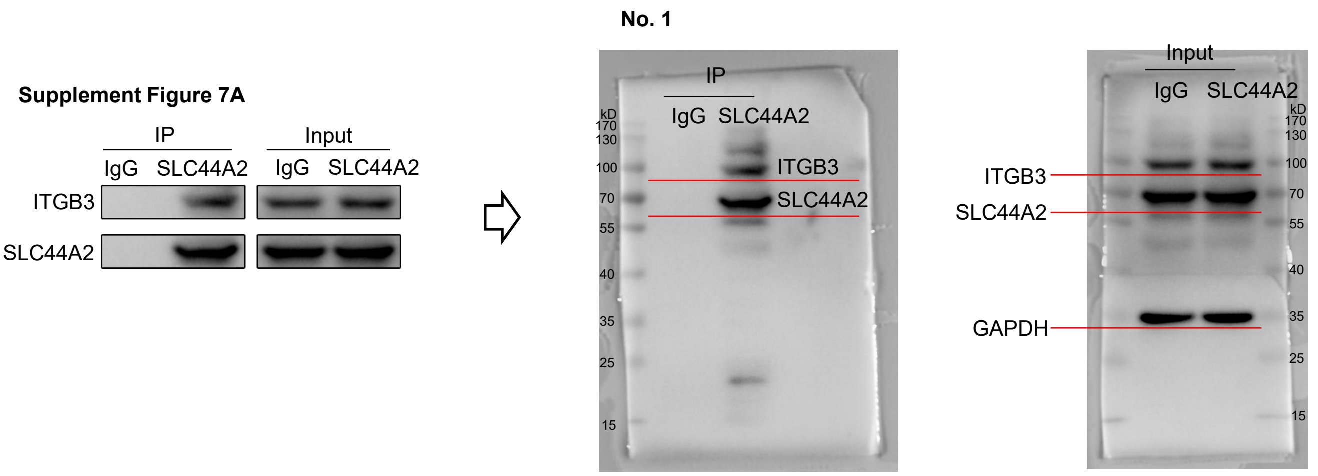

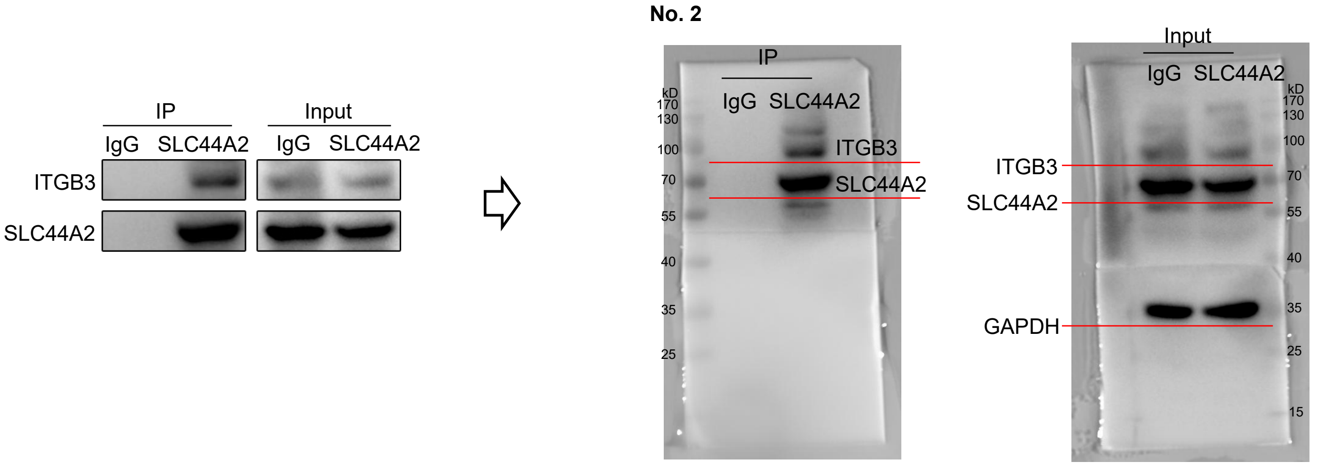

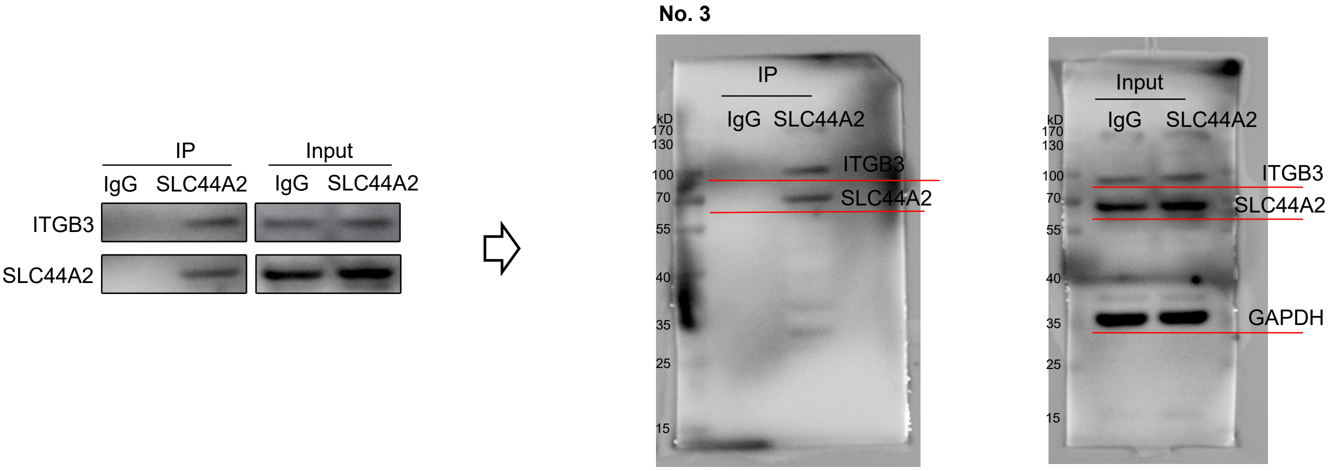

Full unedited gel for Supplement Figure 7B   N=4

Supplement Figure 7B

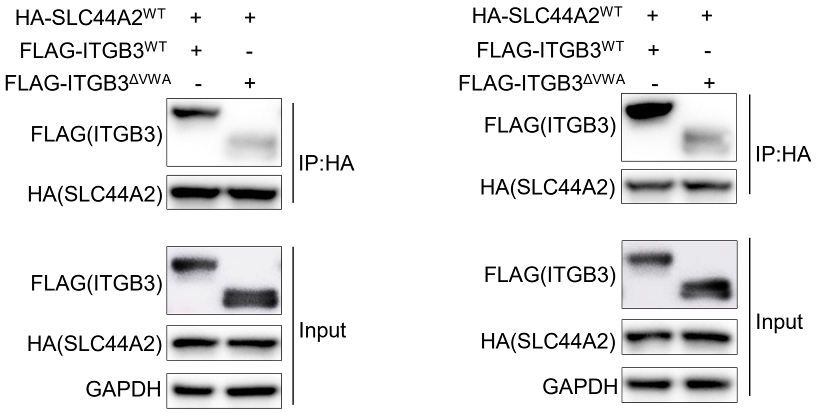

No. 1-2

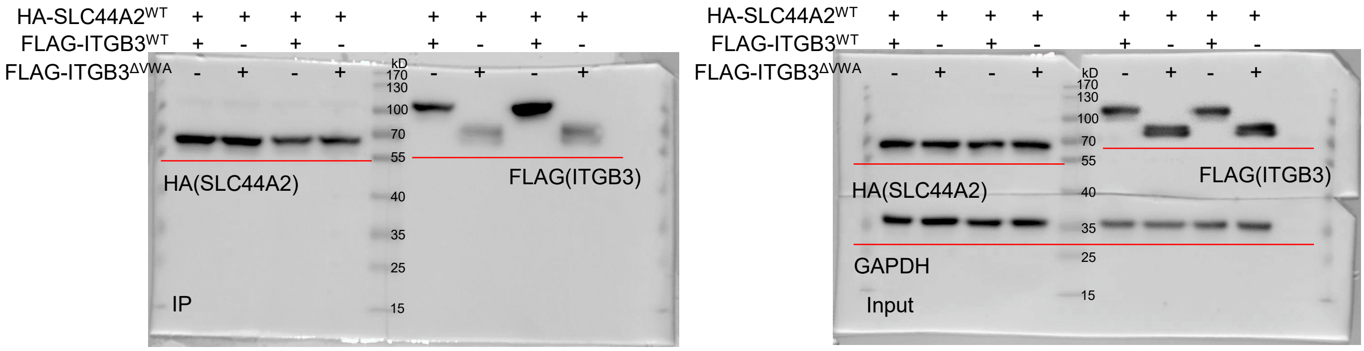

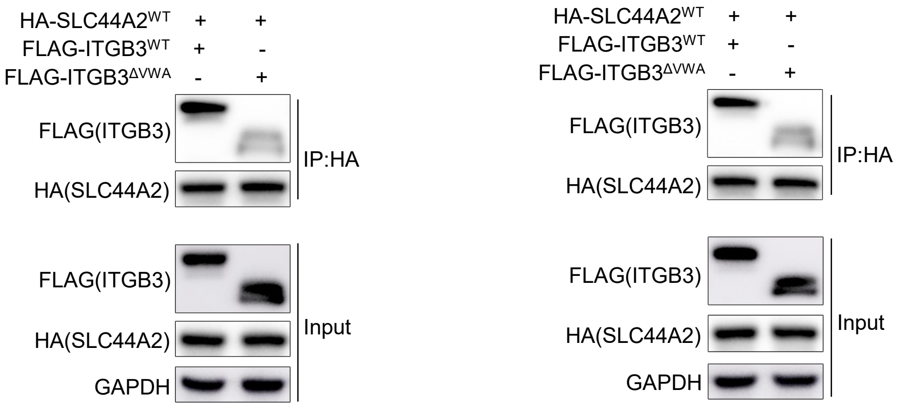

No. 3-4

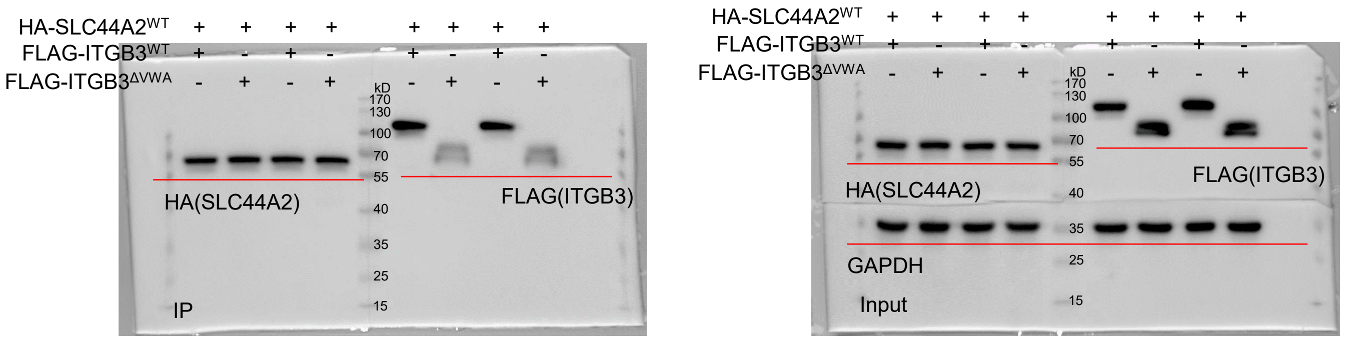

Full unedited gel for Supplement Figure 7E   N=3

Supplement Figure 7E

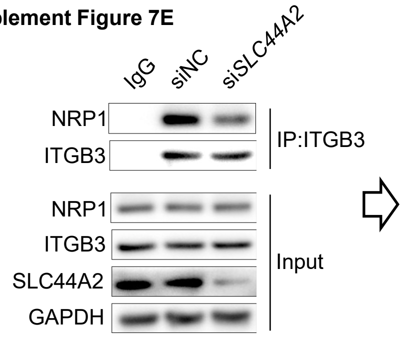

No. 1

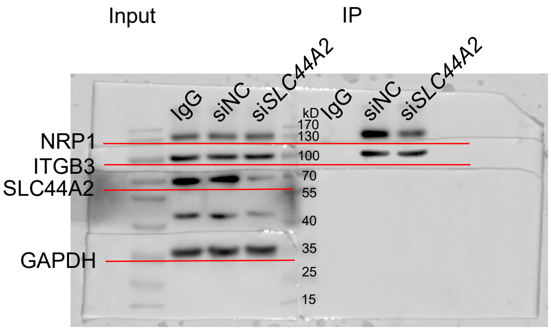

No. 2

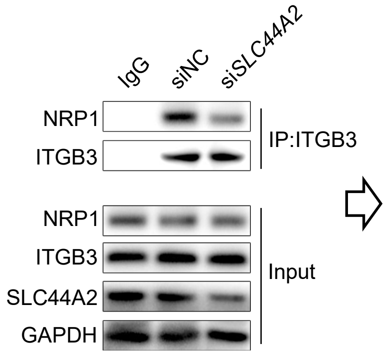

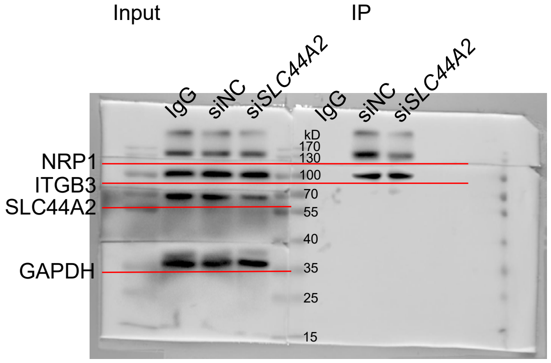

No. 3

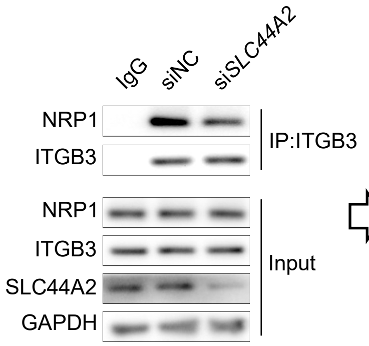

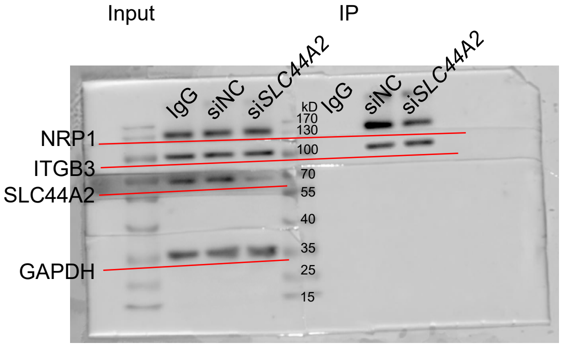

Full unedited gel for Supplement Figure 7F N=3

No. 1

Supplement Figure 7F

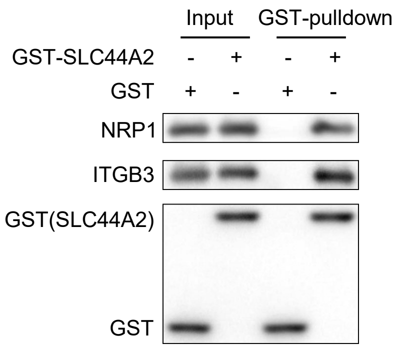

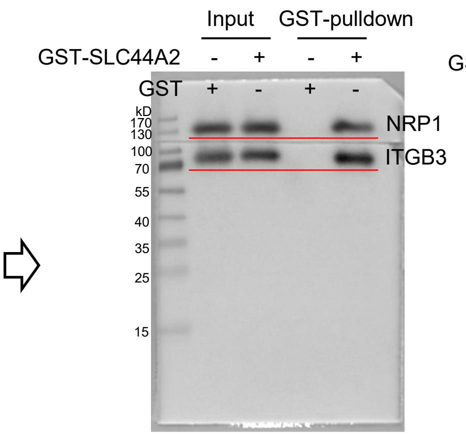

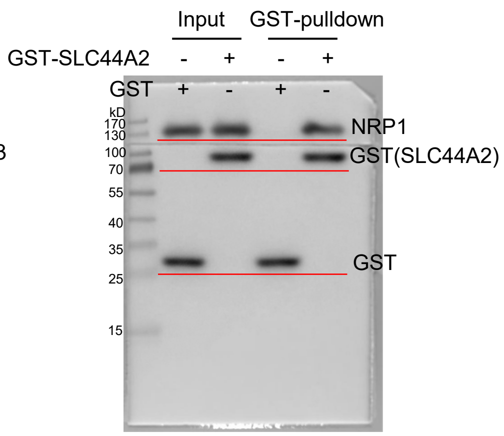

No. 2

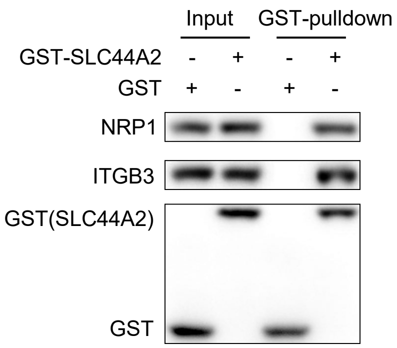

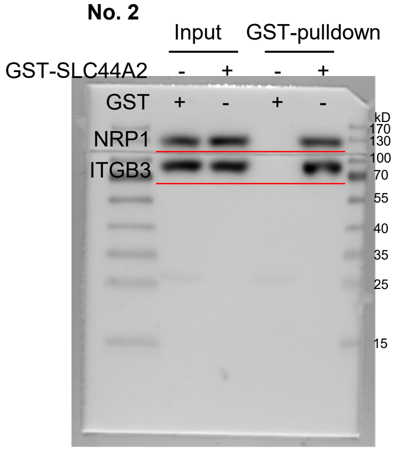

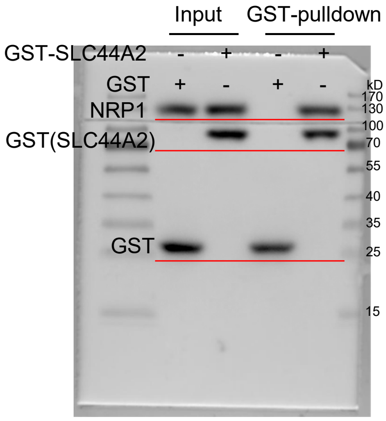

No. 3

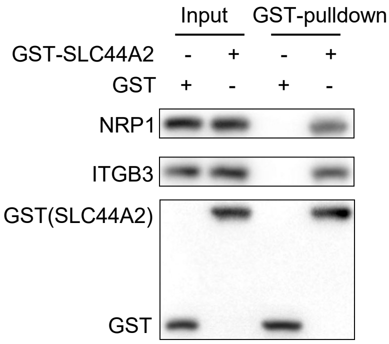

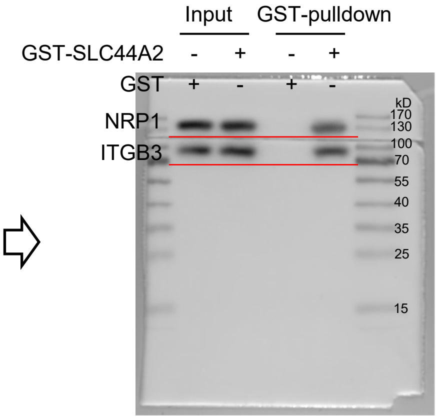

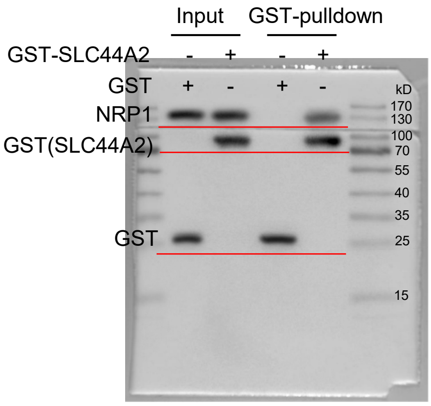

Full unedited gel for Supplement Figure 7G N=3

Supplement Figure 7G

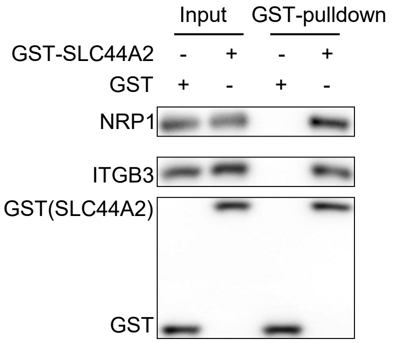

No. 1

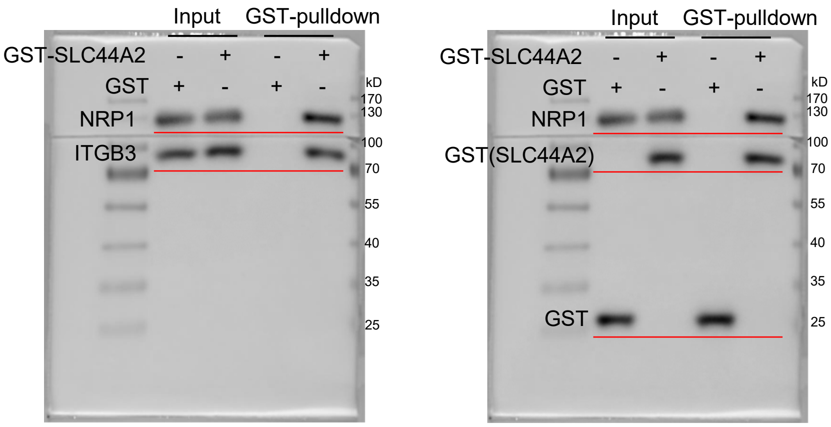

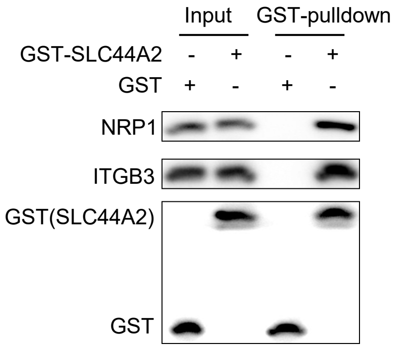

No. 2

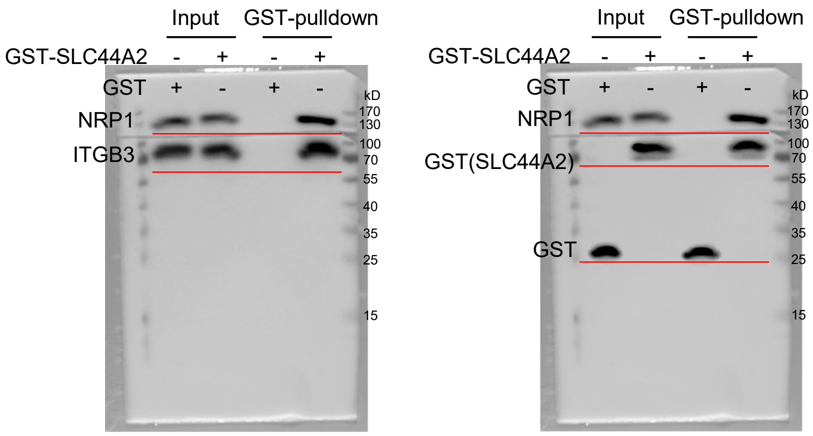

No. 3

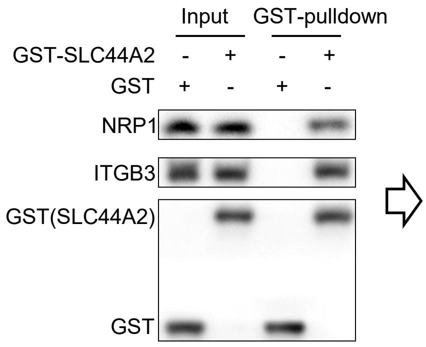

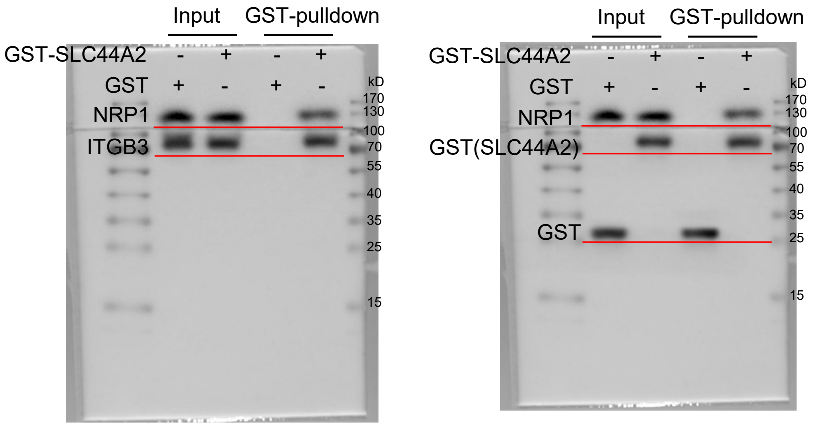

Full unedited gel for Supplement Figure 9A   N=3

Supplement Figure 9A

No. 1

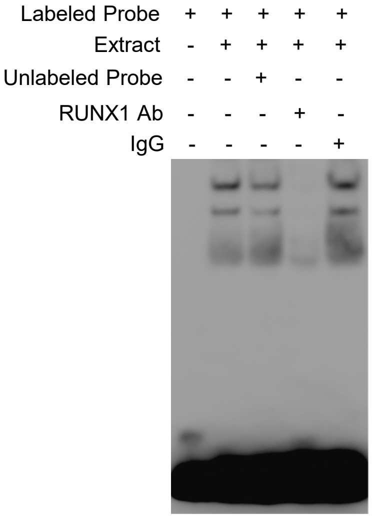

No. 2

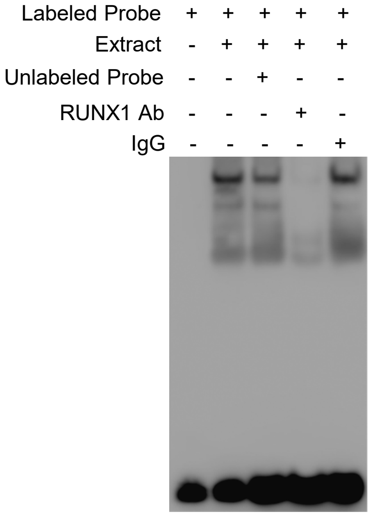

No. 3

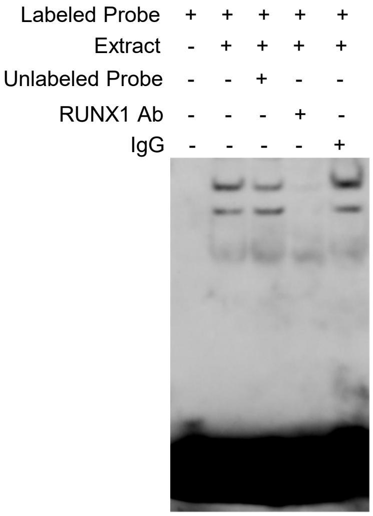

Full unedited gel for Supplement Figure 9C N=5

Supplement Figure 9C

No. 1

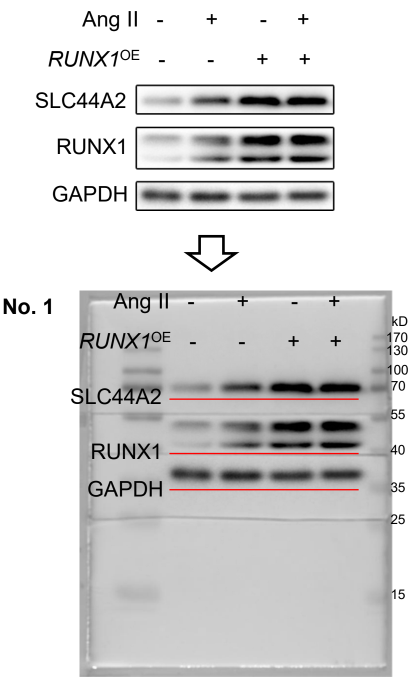

No. 2

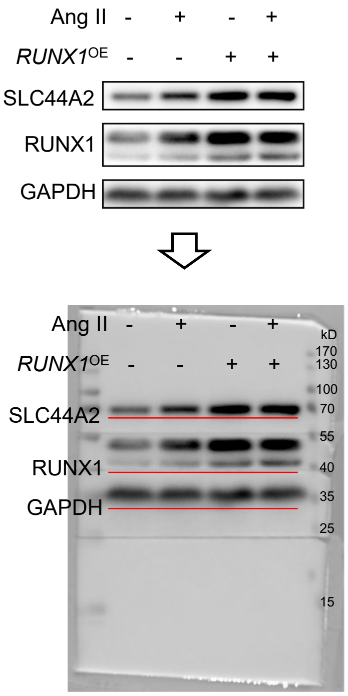

No. 3

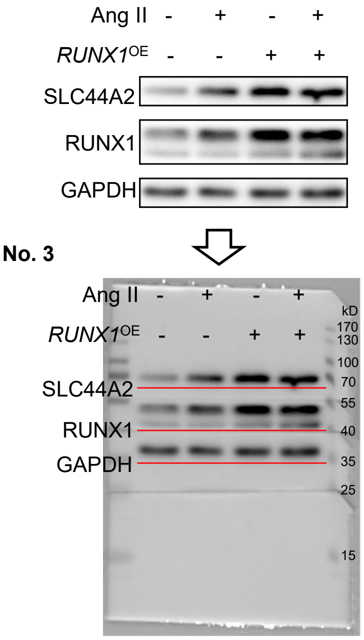

No. 4

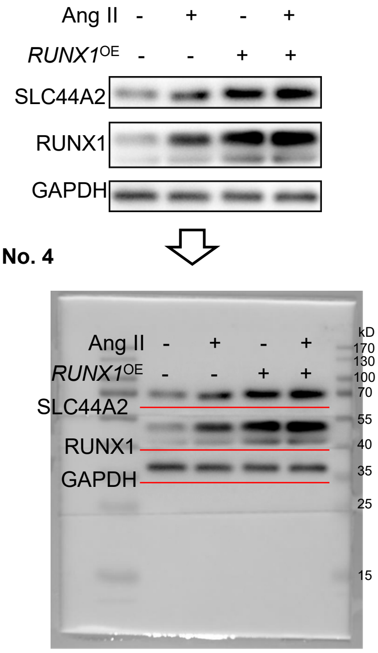

No. 5

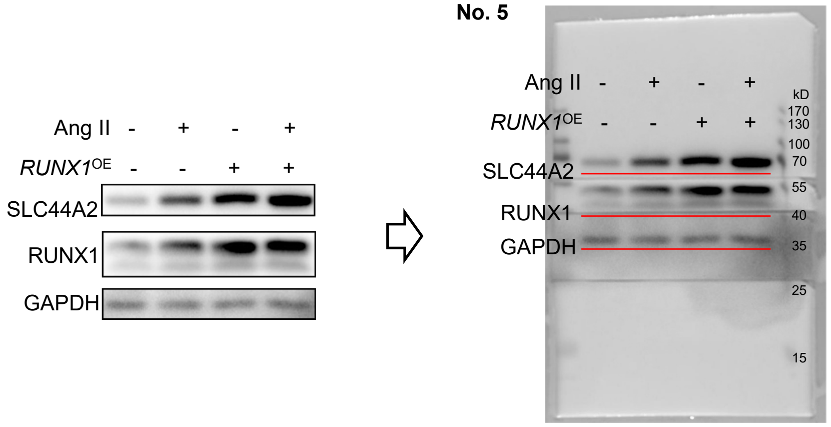

Full unedited gel for Supplement Figure 9D N=3

Supplement Figure 9D

No. 1

Labeled Probe	+	+	+	+	+
Extract	-	+	+	+	+
Unlabeled Probe	-	-	+	-	-
RUNX1 Ab	-	-	-	+	-
IgG	-	-	-	-	+

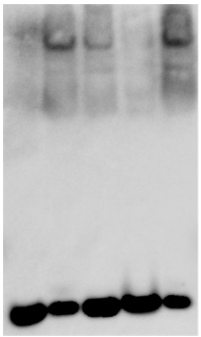

No. 2

Labeled Probe	+	+	+	+	+
Extract	-	+	+	+	+
Unlabeled Probe	-	-	+	-	-
RUNX1 Ab	-	-	-	+	-
IgG	-	-	-	-	+

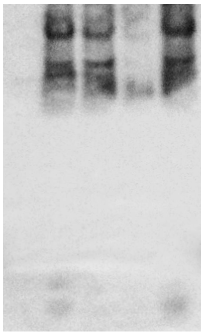

No. 3

Labeled Probe	+	+	+	+	+
Extract	-	+	+	+	+
Unlabeled Probe	-	-	+	-	-
RUNX1 Ab	-	-	-	+	-
IgG	-	-	-	-	+

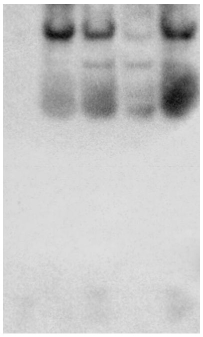

Full unedited gel for Supplement Figure 9F N=5

Supplement Figure 9F

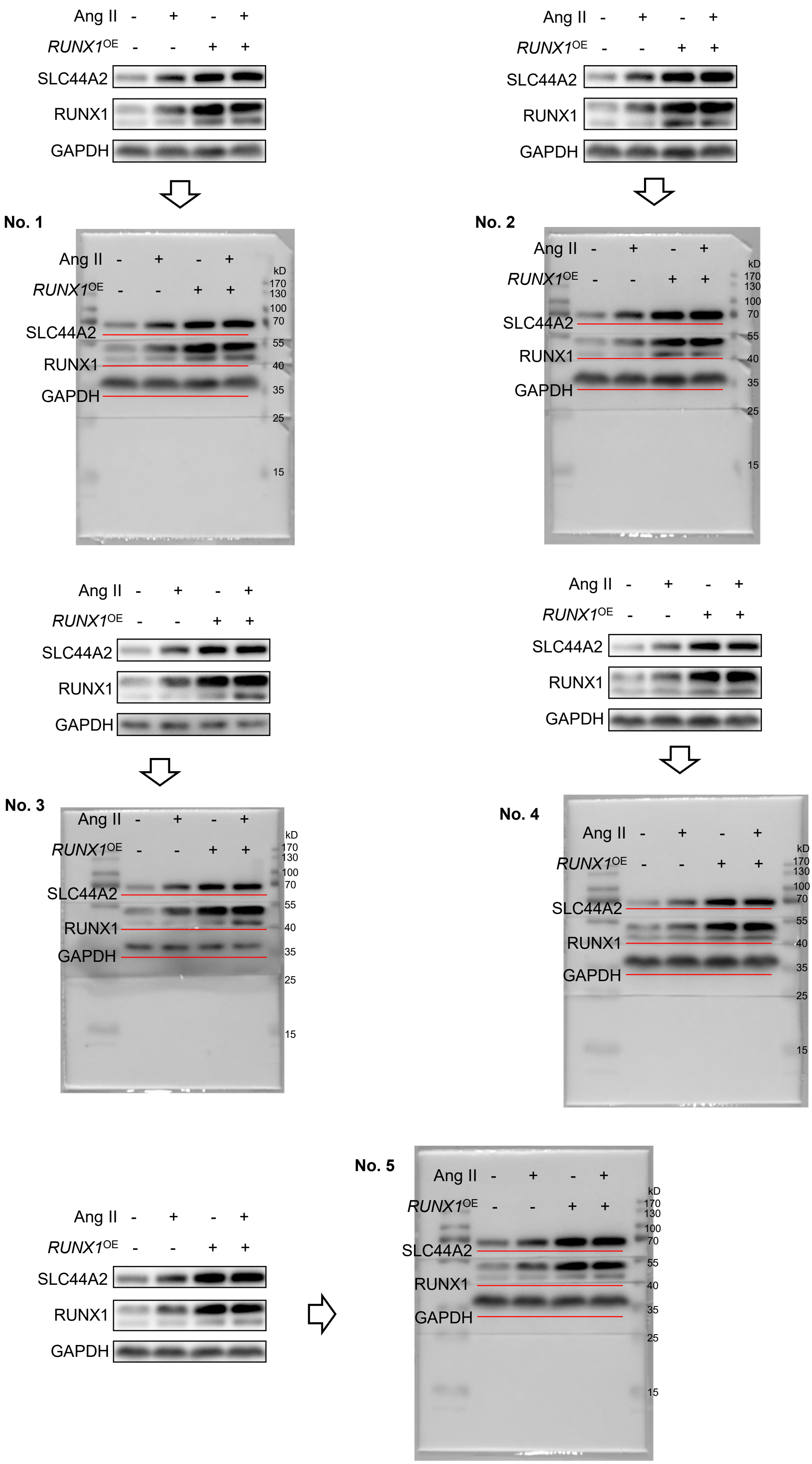

Full unedited gel for Supplement Figure 11A   N=5

Supplement Figure 11A

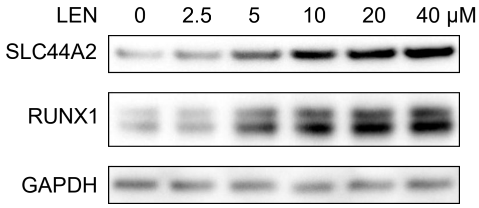

No. 1

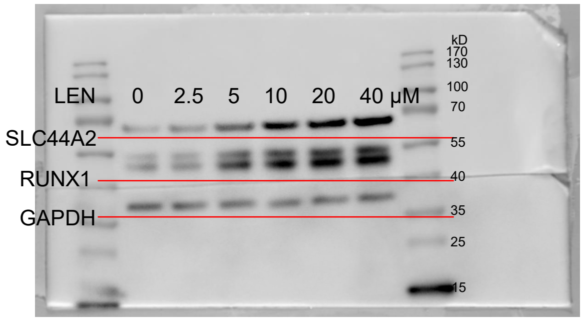

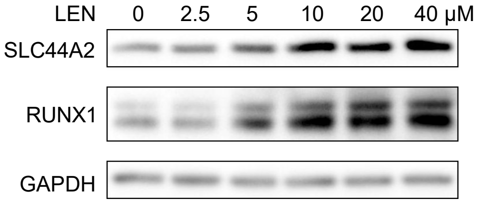

No. 2

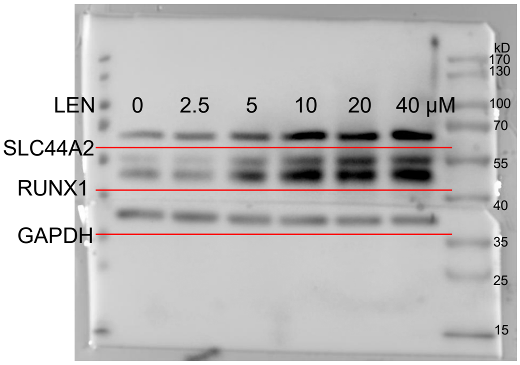

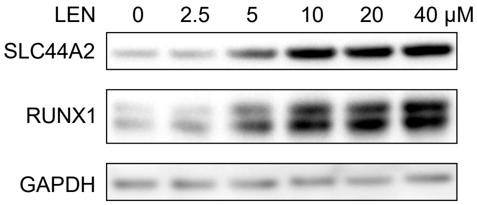

No. 3

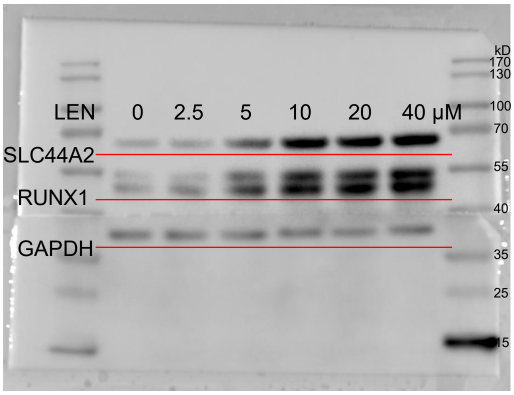

Full unedited gel for Supplement Figure 11A   N=5

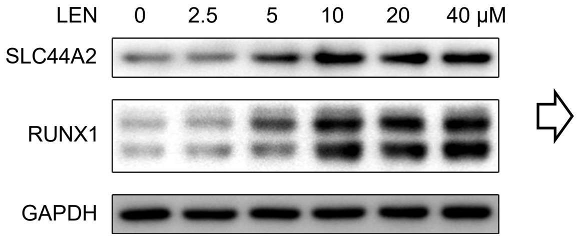

No. 4

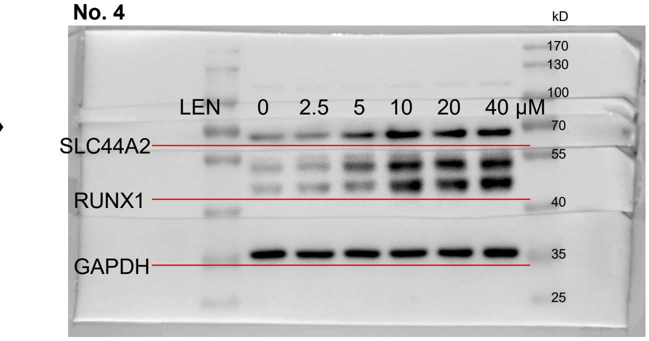

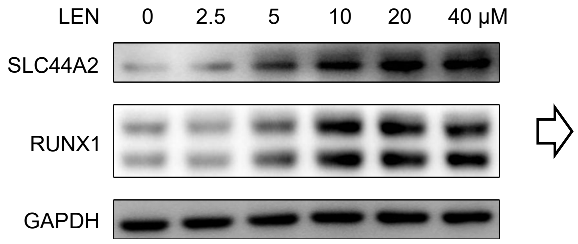

No. 5

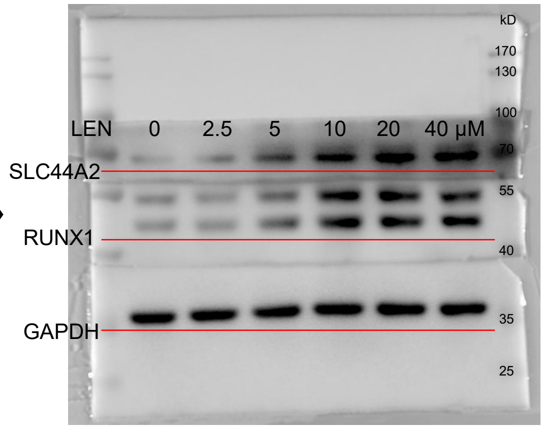

Full unedited gel for Supplement Figure 11B N=5

Supplement Figure 11B

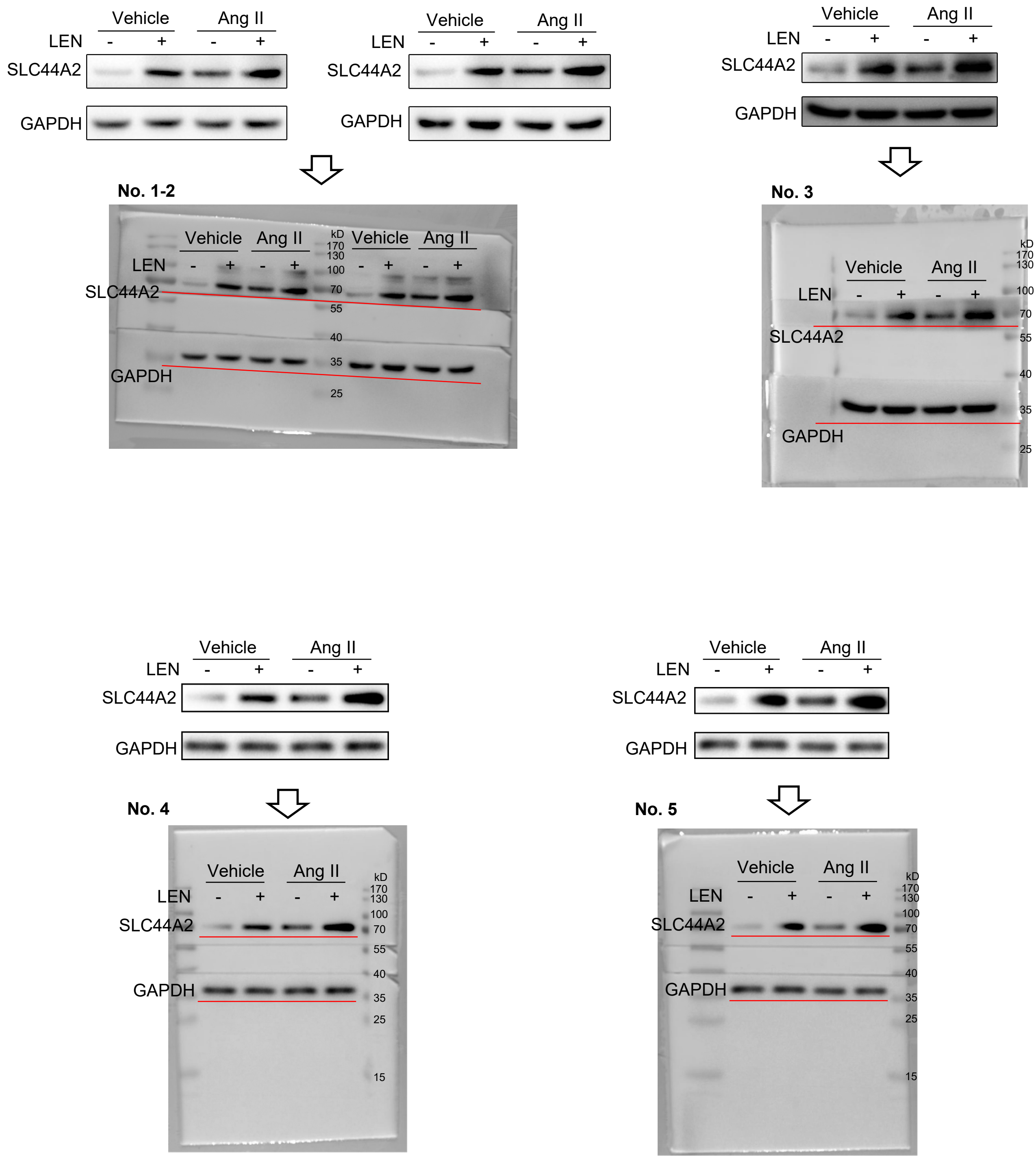

Full unedited gel for Supplement Figure 11C   N=5

Supplement Figure 11C

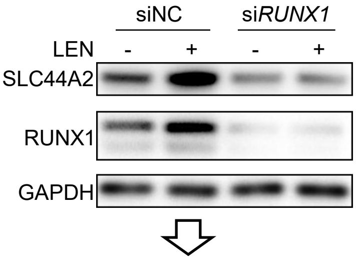

No. 1

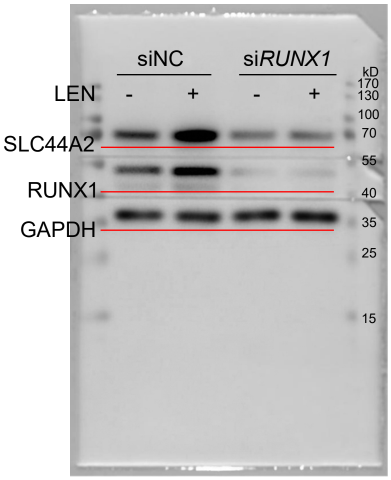

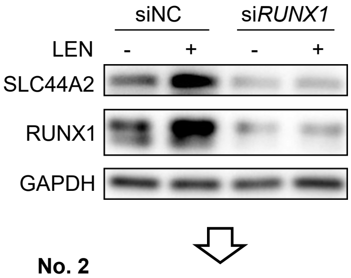

No. 2

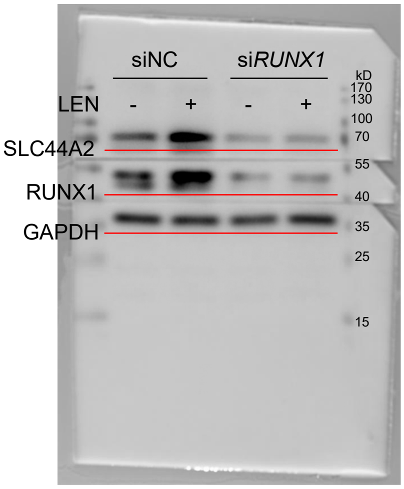

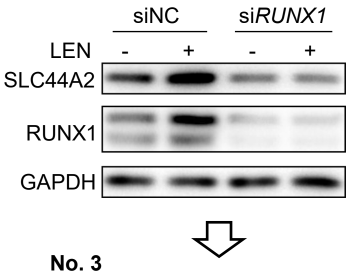

No. 3

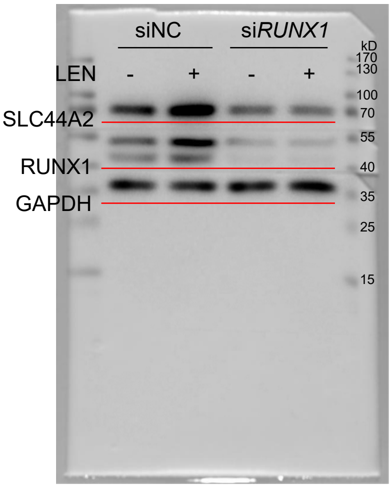

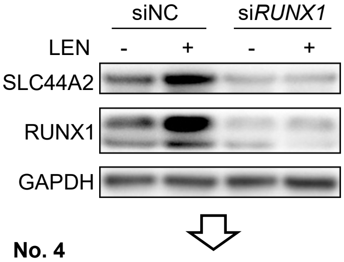

No. 4

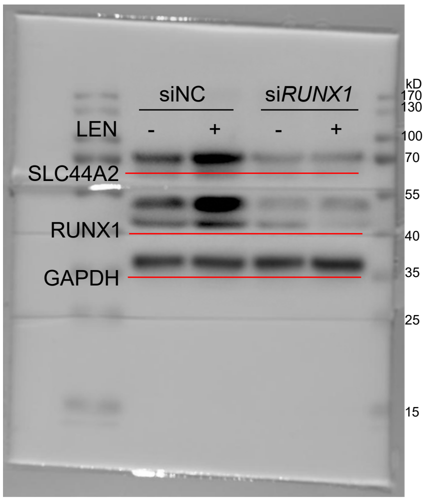

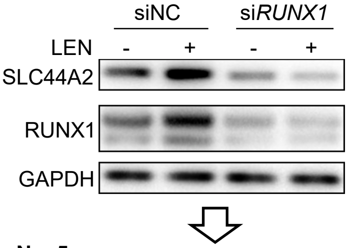

No. 5

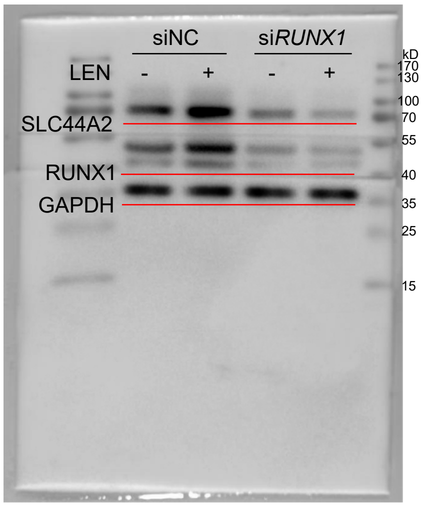

Full unedited gel for Supplement Figure 12I N=5

No. 1

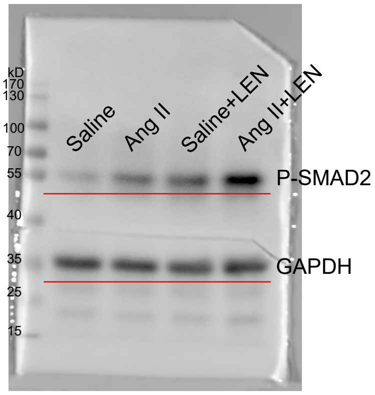

Supplement Figure 12I

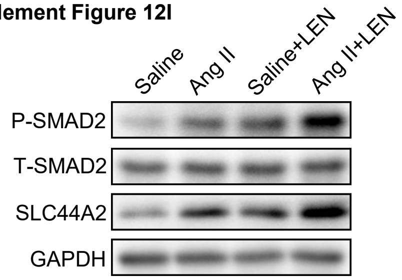

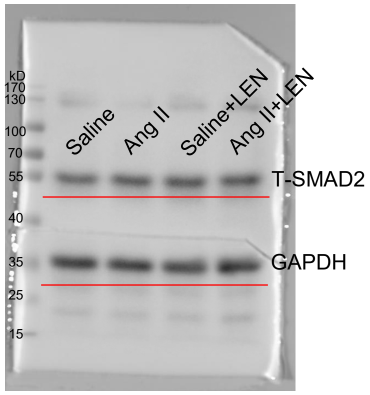

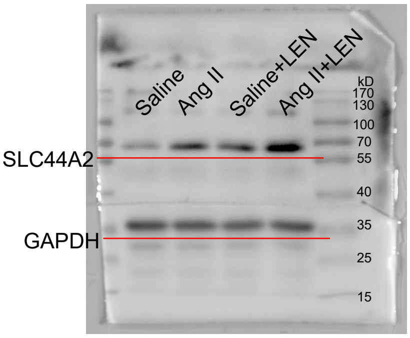

Full unedited gel for Supplement Figure 12I   N=5

No. 2-3

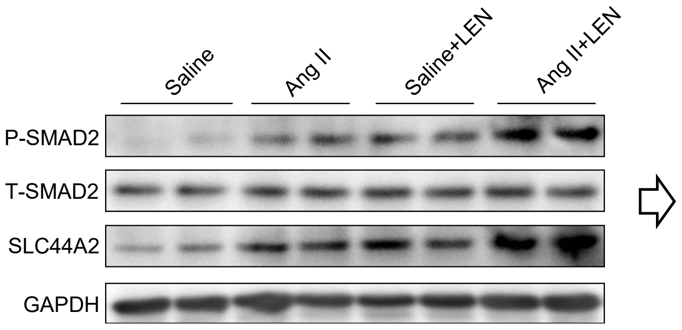

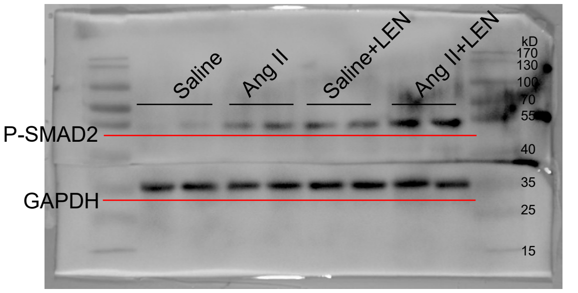

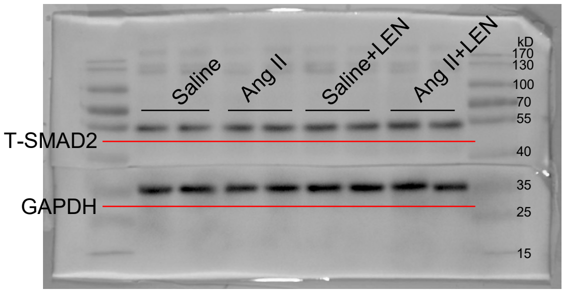

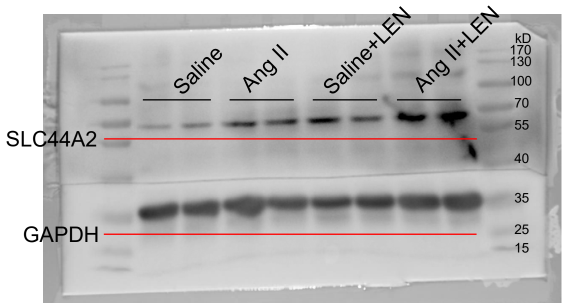

Full unedited gel for Supplement Figure 12I N=5

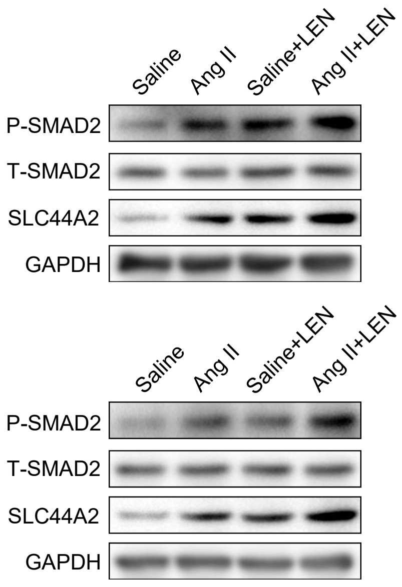

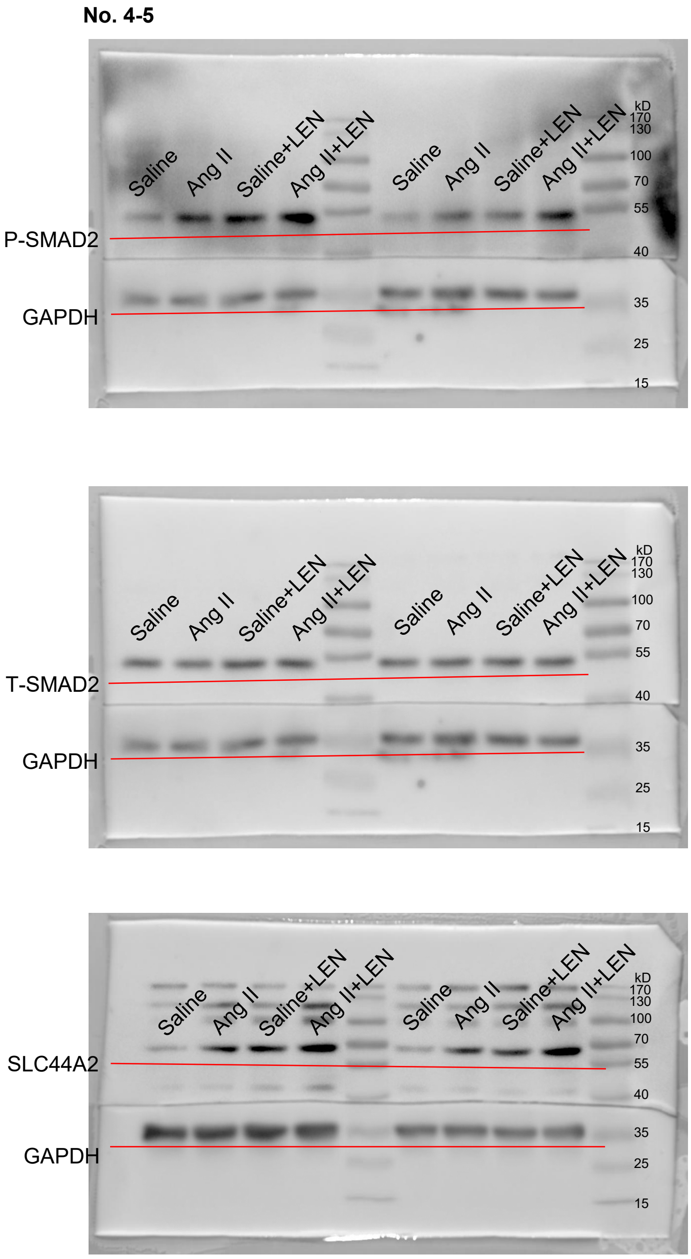

Supplement: Unedited blot and gel images [file jci-134-173690-s013.pdf]
